# Development and validation of a physiology-based model for the prediction of pharmacokinetics/toxicokinetics in rabbits

**DOI:** 10.1371/journal.pone.0194294

**Published:** 2018-03-21

**Authors:** Panteleimon D. Mavroudis, Helen E. Hermes, Donato Teutonico, Thomas G. Preuss, Sebastian Schneckener

**Affiliations:** 1 Bayer AG, Engineering & Technology- Systems Pharmacology, Leverkusen, Germany; 2 Bayer Crop Science, Environmental Safety, Monheim, Germany; University of Kentucky, UNITED STATES

## Abstract

The environmental fates of pharmaceuticals and the effects of crop protection products on non-target species are subjects that are undergoing intense review. Since measuring the concentrations and effects of xenobiotics on all affected species under all conceivable scenarios is not feasible, standard laboratory animals such as rabbits are tested, and the observed adverse effects are translated to focal species for environmental risk assessments. In that respect, mathematical modelling is becoming increasingly important for evaluating the consequences of pesticides in untested scenarios. In particular, physiologically based pharmacokinetic/toxicokinetic (PBPK/TK) modelling is a well-established methodology used to predict tissue concentrations based on the absorption, distribution, metabolism and excretion of drugs and toxicants. In the present work, a rabbit PBPK/TK model is developed and evaluated with data available from the literature. The model predictions include scenarios of both intravenous (i.v.) and oral (p.o.) administration of small and large compounds. The presented rabbit PBPK/TK model predicts the pharmacokinetics (Cmax, AUC) of the tested compounds with an average 1.7-fold error. This result indicates a good predictive capacity of the model, which enables its use for risk assessment modelling and simulations.

## Introduction

Environmental and human risk assessments are based on standardized biotests that are conducted on different species. To minimize the use of animals, the 3R principle (i.e., reduction, replacement, and refinement) requires that animal experiments are substituted with appropriate alternative test methods whenever possible [1–4]. This principle is already a current practice in REACH [5, 6], which is a binding European Union regulation regarding the production and use of chemical substances. Additionally, in the European pesticide regulation 1107/2009, it is clearly stated that the use of non-animal test methods and other risk assessment strategies should be promoted, and animal testing should be minimized as tests on vertebrates should be undertaken as a last resort. However, despite the regulations in place, the use of alternative methods in pesticide risk assessments is limited. Since pesticides are designed to act on biological systems, they frequently have specific modes of actions. For these specific modes of action, reliable *in silico* methods are much harder to establish than for chemicals for which the baseline toxicity is assumed. In this regard, mathematical modelling can be used for for translations between different exposure routes and species. This method reduces animal testing, which is otherwise conducted to understand the effect under multiple exposure or species situations.

Physiologically based pharmacokinetic (PBPK) modelling is a technique used to simulate the pharmacokinetics (PK) of a substance in different animals and tissues [7–10]. In the case of toxicant risk assessments, the term physiologically based toxicokinetic (PBTK) models is preferably used to emphasize the application of the models to compounds that cause toxic responses [11]. In PBPK/TK models, the tissues of organisms are simulated as a group of physiologically based compartments of different volumes, blood flows, and other tissue composition elements. These compartments provide a mechanistic framework for evaluating the absorption, distribution, metabolism, and excretion (ADME) of a drug or toxicant in the body. Compared to other type of models, PBPK/TK models separate the information on the physicochemistry of a compound from the characteristics of an organism [12]. Therefore, the physiological model parameters are independent of the compound-specific parameters. Consequently, the physiological parameters can be used across different compounds and the compound parameters can be used across species [13]. Active processes involve parts of the physiology and properties of the compound. Hence the process itself may be translated (for homologues pathways), but the actual kinetic parameterisation may be very different.

PBPK models are standard tools in pharmacology and are frequently used to support the prediction of clinical pharmacokinetics, drug-drug interactions, dose scaling for children, and formulation/adsorption development [14]. Regulatory agencies such as the European Medicines Agency (EMA) and the Food and Drug Administration (FDA) encourage the use of PBPK models, and these models are often part of the submission of new medical entities [15, 16]. Also, World Health Organization (WHO) and the United States Environmental Protection Agency (US-EPA) underlines the necessity to establish common principles for their application in chemical hazard and risk assessment [17]. Despite that the use of PBPK/TK models is encouraged in risk assessment by several recent reports and articles [18], their utilization is limited. This limited use is surprising given the potential for translating the information from standard laboratory species such as rats, rabbits, and mice to physiologically related small (wild) animals.

The aim of this work is to develop and validate a rabbit PBPK/TK model. The rabbit is a common laboratory animal as well as a relevant species for environmental risk assessment. Hence, more information on rabbit physiology is available than for other relevant wild species (e.g., vole or wood mouse). An extensive literature search was performed, and the gathered physiology information was integrated into a PBPK/TK model. Next, a set of compounds with increasing complexities regarding administration and clearance profiles was used for validation. The validation set included experiments with intravenous injection and oral administration, compounds with different excretion and metabolisation profiles (renal and hepatic clearance), and measurements of compound concentrations in different tissues. By considering a diverse set of compounds, different aspects of the model were validated, such as general physiology, renal versus hepatic (i.e., metabolic) clearance, and properties of the gastrointestinal tract (GIT). The model was validated by comparing the model predictions to measured data. The discrepancies between observations and the simulation were resolved by optimizing the parameters or retrieving independent information from the literature.

## Materials and methods

### Model structure and parameterization

**Fig 1:**
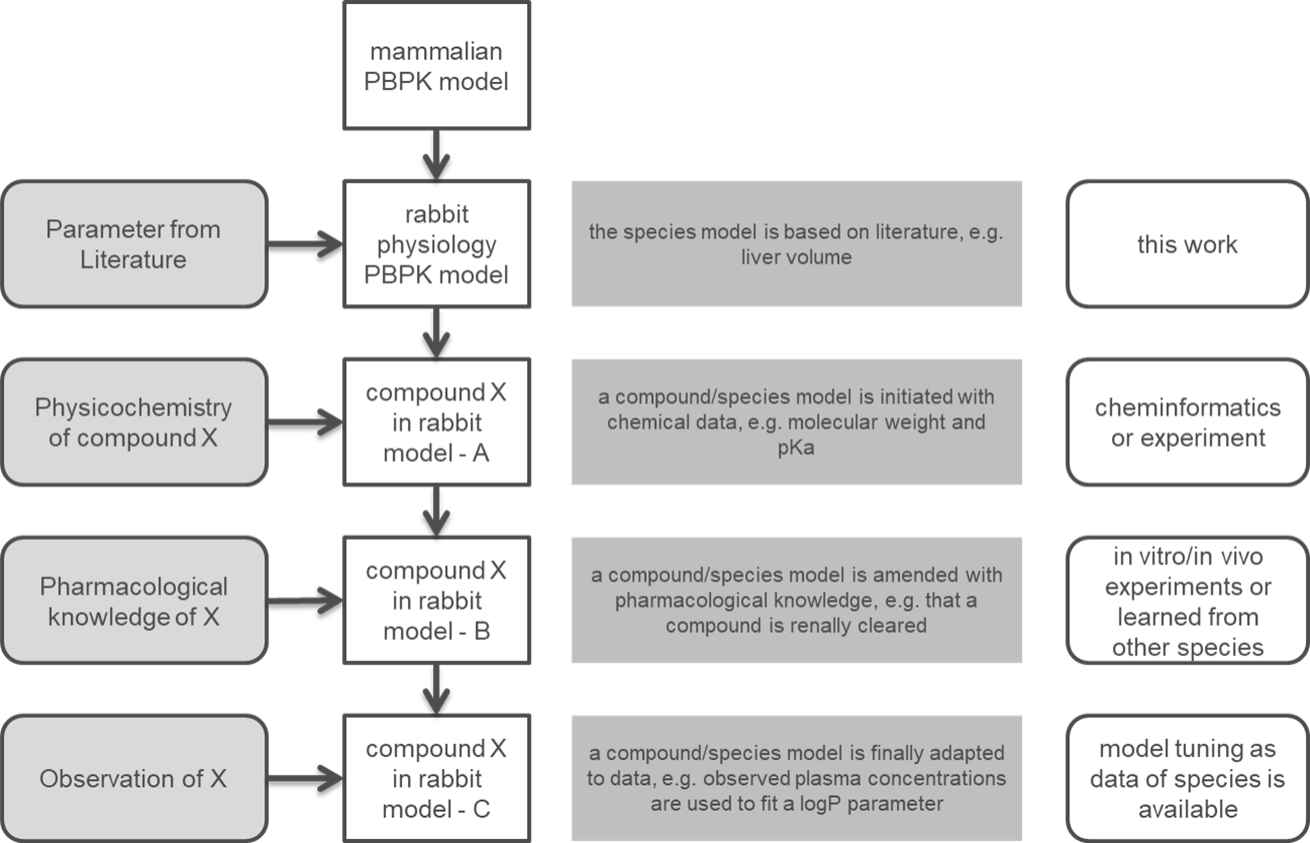
Workflow of the rabbit PBPK/TK model development. Starting with a generic mammalian PBPK model on top, rabbit specific physiology parameters are added (first level). Next, physicochemistry from a compound (second level), followed by information regarding active processes (third level) are introduced from literature, in-house experiments or transferred from other species. Lastly, PK data are used to identify additional parameter values of the model (fourth level).

For a comprehensive PBTK model as described here, a stepwise approach is typically used to inform the parameters. The first level retrieves the parameter values from the literature to describe the general properties of the species and is independent of the compound to be modelled. Second, the modelling of a specific compound starts by informing the model with the physicochemical properties of the molecule. This information is readily available or can be calculated independently [19]. Third, the active processes (e.g., transport and metabolism) are parameterized based on the information of *in vitro* or *in vivo* assays. Information from other species can be also used when there is no information for the species of interest. Lastly, if exposure data for a compound in the target species is available, this can be used to fine-tune the selected parameters. A schematic workflow of the model establishment is shown in Fig 1. In our model, physiological parameters of rabbit were introduced in the PK-Sim^®^ framework in order to represent rabbit physiology (first level). The parameters identified from the literature are shown in supporting information Tables A-J in S1 Appendix. Furthermore, a complete list of the rabbit PBPK model parameters is shown in Table O in S1 Appendix. If parameters of rabbit physiology were not available in the literature, the corresponding parameters of a pre-existing PBPK model were used. For this, the model of the taxonomically related mouse was used. Compound related information (second level) and information for respective active processes (third level) were then adopted from online databases and in house experiments and are shown in Tables K-L in S1 Appendix. Finally, fine-tuning (fourth level) was performed by fitting the rabbit model to available experimental data (S1–S10 Figs).

**Fig 2:**
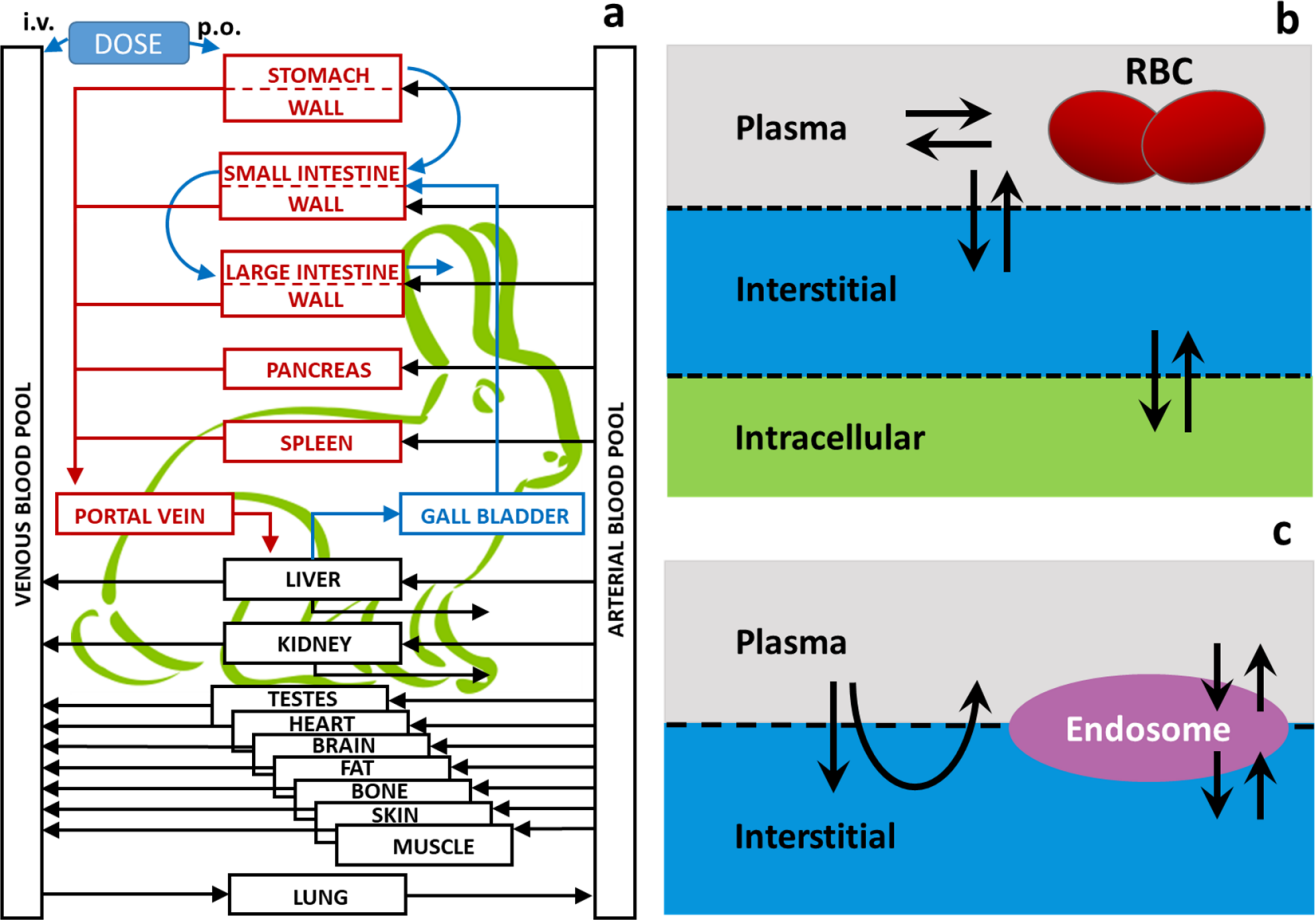
Rabbit PBPK model structure. a: Generic model structure implemented in the PK-Sim^®^ software suite. b: The sub-compartments of the tissues and the distribution scheme for the case of small molecules (RBC: red blood cells), c: The sub-compartments of the tissues and the distribution scheme for the case of large molecules.

The rabbit model is based on the mammalian physiological framework implemented in the PK-Sim^®^ software platform [20]. This whole-body physiology-based pharmacokinetic framework provides a detailed mathematical representation of ADME processes in humans and several common laboratory animals. In essence, the mammalian body is divided into compartments representing the relevant organs or tissues as well as the arterial and venous blood pools that connect the different organs via blood flow (Fig 2A). Organs are further divided into sub- compartments that describe the vascular space (divided into plasma and red blood cells) as well as the avascular space (divided into interstitial and cellular space) (Fig 2B and 2C). The characteristics of the administered compound (small or large molecules such as proteins) determine its distribution by either diffusion through membranes (Fig 2B) or by pores and endosomal clearance (Fig 2C). A substantial amount of experimental information has been used to inform and calibrate the biological and physiological processes included in the model. The model has successfully been used to describe the uptake and distribution of a wide range of compounds in the species included to date [10, 21–26].

Based on the current animal models implemented in the PK-Sim^®^ software suite, the model for a 2.5 kg rabbit was built by adjusting the values of organ volumes (Table A in S1 Appendix), haematocrit (Table B in S1 Appendix), specific blood flow rates (Table C in S1 Appendix), GIT compartment pH (Table D in S1 Appendix), GIT compartment dimensions (Tables E-F in S1 Appendix), GIT compartment transit/emptying times (Table G in S1 Appendix), and the effective surface areas of the GIT-small intestine compartments (Table I in S1 Appendix) according to rabbit physiology values found on literature. For most of the parameters, there was more than one value available from the literature. In most cases, the relative differences among the different sources were not large. The sources that provided information for as many tissues as possible were selected to achieve a consistent representation. Therefore, most organ volumes/specific blood flow rates were taken from [27], GIT-pH values were taken from [28], and GIT-proximal/distal radius and length values were taken from [29]. In the case of gastric emptying time, since the reported values in the literature had large deviations (i.e., from 20 min to 8 hrs), different values were chosen based on the experiment. Similarly, for the large/small intestinal transit rates, the full range of values was considered to set up the model (see Supporting information-GIT-transit emptying times, Tables G-H in S1 Appendix). For the parameter values not found in the literature, the mouse PBPK model parameters were transferred and used, as it is the closest animal species to the rabbit among the species available in the physiology database (e.g., mouse retains a gallbladder unlike the rat).

### Validation process

The rabbit model was informed, tested and verified using published data for a range of compounds in which the clearance pathways were known from either the literature or from in-house data (Table K in S1 Appendix). The choice of compounds took a hierarchical approach beginning with compounds cleared solely by either the kidney or the liver, which are the two main excretory organs, followed by the compounds cleared by both organs (i.v. administration). Subsequently, after assessing the physiology of the kidney and the liver, increasingly more complex clearance processes and administration protocols (p.o. administration) were simulated. For each simulation, the model prediction was first compared with the available data. In the case of deviations, either those parameters are known to vary among species or the parameters with no prior information were optimized. For all simulations, the partition coefficients and the cellular permeabilities were calculated based on the PK-Sim^®^ Standard distribution model [22, 30]. In short, partition coefficients describe the partition of a compound between water and lipids or water and proteins. They are calculated using a mechanistic formula similar to that described in [31]. The product of organ surface area and permeability controls the rate of permeation across lipid cell membranes from plasma into organs. Calculation of the permeabilities was based on the semi-empirical formula first published in [32] that accounts for transcellular as well as paracellular passive transport for small molecules. For large molecules, the two pores transport model is used [33] without accounting for FcRn binding.

### Sensitivity analysis

To evaluate the sensitivity of our model to parameter perturbations, a local sensitivity analysis was performed. At every step of the sensitivity analysis, one parameter *j* of the model was varied by 10% of its nominal value while the remaining model parameters were kept constant. Next, the sensitivity coefficients (s_i,j_) for the AUC or C_max_ outputs were calculated as (Eq 1):

$$s_{i,j}=\frac{{\partial f}_{i}}{\partial x_{j}}$$ (1)

where ∂*f_i_* is difference in PK parameter (AUC or C_max_) between perturbed and un-perturbed simulation and ∂*x_j_* is the difference between varied and nominal parameter value. PK parameters of the sensitivity analysis were constrained to C_max_ and AUC as the most relevant PK parameters for evaluating drug toxicity/efficiency. The sensitivity analysis was restricted to parameters of the rabbit model while parameters specific for compounds were not considered.

### Availability of rabbit PBPK/TK model

The rabbit PBPK/TK model presented in this work and the software PK-Sim^®^ are available in the Open Systems Pharmacology Suite, which is now developed as an open source platform (https://github.com/Open-Systems-Pharmacology/Suite/releases/tag/v7.1.0). The data used to calibrate and validate the model were collected from the literature and referenced where used.

## Results

### Model validation

First, a rabbit PBPK/TK model was developed, based on the pre-existing mammalian model structure and relative parameters retrieved from the literature or transferred from other mammalian PBPK/TK models. The rabbit PBPK/TK model structure with all considered organs and interactions is shown in Fig 2A. The details of the model, the structure and the parameters are listed in the supporting information. Second, to assess the reliability of the model, six different compounds (i.e., inulin, caffeine, ofloxacin, theophylline, paracetamol, and acyclovir) were modelled. Since inulin is solely filtered by the glomerulus of the kidney but is not secreted or reabsorbed by the tubulus or metabolized, it is well suited to validate the renal glomerular filtration rate (GFR) in the rabbit. To simulate renal excretion, the GFR was set to 100%, a value that is expected for renally cleared molecules. After determining the renal function of the rabbit, caffeine was tested since it is predominantly metabolized by the liver (S2 Fig). The metabolic processes were always localized in the liver as hepatic clearance, and the respective reaction kinetics were optimized based on the available data. Next, more complex compounds such as ofloxacin and theophylline were used (S3 and S4 Figs). To assess the rabbit GIT, oral administration was further added to test the model. The validation of oral administration was based on the compounds paracetamol, theophylline, and acyclovir. In the following sections, the detailed results for the compounds inulin and paracetamol are shown as they provide information for multiple tissues (inulin) and multiple oral formulations (paracetamol).The model results for the additional compounds are described in the supporting information and are only summarized here.

### Inulin case study

**Fig 3:**
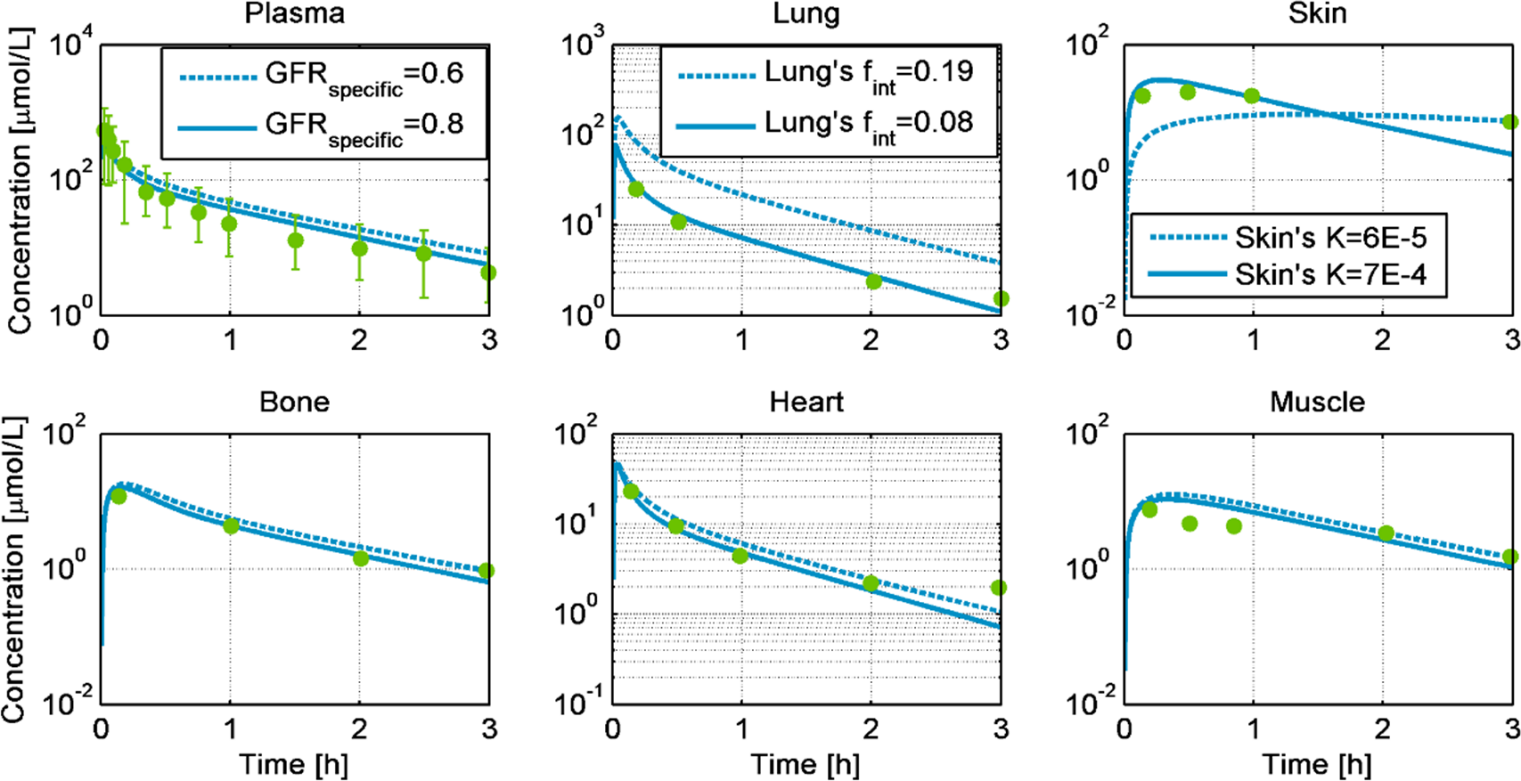
Simulations of the concentration profiles of inulin in venous blood plasma, heart, muscle, bone, lung, and skin, for 200 mg/kg intravenous administration in New Zealand white rabbits weighing 2.5 kg. The dotted lines represent the predicted profiles, while the solid lines show the simulated profiles after the GFR_specific_, skin’s hydraulic conductivity (*K*), and lung’s fraction interstitial (f_int_) were adjusted. The green dots are experimental observations from [34].

Fig 3 compares the observed PK-data from 200 mg/kg inulin after i.v. administration in rabbits weighing 2.5 kg [34] to the PBPK/TK model simulations in different tissues. The dotted lines represent the rabbit model simulations with no parameter calibration. The rabbit PBPK model predicts the PK profiles very close to the experimental data for the muscle, heart, and bone tissues without any further parameter adjustments (Fig 3 dotted lines and Table 1). For the plasma, skin and lung, the model was adjusted to better agree with the observed data. Here, the specific glomerular filtration rate (GFR_specific_) was increased from 0.6 to 0.8 [l/min/kg-kidney-weight] [35] to better explain the observed exposure in the plasma. Similarly, the hydraulic conductivity of the skin was increased in accordance with the inulin simulations performed in rats [21, 36]. Finally, to describe the PK profile in the lungs, the interstitial fraction of the lung was increased. These adaptions to the model are all in accordance with the literature. The simulation with the three adjusted parameters indicates a correct distribution of the compound among the different tissues of the rabbit (Fig 3-solid lines).

**Table 1:** Comparison of experimentally observed AUC, predicted AUC, and AUC after parameter optimization for available compartments, along with the relative errors, administration details and references. Inulin simulation efficiently described the data adopted from [37] and as such no further optimization was performed.

Compounds	AUC observed	AUC Predicted	AUC Optimized	% AUC Error (Predicted)	% AUC Error (Optimized)	Tissue/Administration	Literature
**Inulin**	842.36	994.63	-	15.31	-	Venous Blood (40 mg/kg)/i.v.	[37]
	1345.61	1491.95	-	9.81	-	Venous Blood (60 mg/kg) i.v. i.v.	[37]
	6901.24	9383.03	7564.91	26.45	8.77	Plasma/i.v.	[34]
	1071.31	3643.40	1186.67	70.60	9.72	Lung/i.v.	[34]
	2438.59	1435.94	2242.76	69.83	8.73	Skin/i.v.	[34]
	660.24	941.45	760.29	29.87	13.16	Bone /i.v.	[34]
	873.24	1045.38	819.07	16.47	6.61	Heart/i.v.	[34]
	620.97	1090.23	884.54	43.04	29.80	Muscle/i.v.	[34]
**Caffeine**	5432.82	7747.60	5739.40	29.88	5.34	Venous Blood/i.v.	[39]
**Theophylline**	23871.43	63755.93	27257.72	62.56	12.42	Venous Blood/i.v.	[40]
	54651.84	57080.23	54084.85	4.25	1.05	Venous Blood/i.v.	[41]
**Theophylline (PO)**	217832.06	86553.63	192065.78	151.67	13.42	Venous Blood/Conventional Tablet	[41]
**Paracetamol (PO)**	2279.80	1836.71	2372.61	24.12	3.91	Venous Blood/Solution	[38]
	1712.76	1958.72	1581.04	12.56	8.33	Venous Blood/Rapid Tablet	[38]
	2225.68	401.05	1335.17	454.96	66.70	Venous Blood/Conventional Tablet	[38]
**Acyclovir (PO)**	6469.00	9089.22	7630.12	28.83	15.22	Venous Blood/Solution	[42]

The results from two additional inulin i.v. doses simulating the experiment of [37] are shown in S1 Fig. These simulations indicate that the rabbit PBPK model explains the available observations without parameter calibration.

### Paracetamol case study

**Fig 4:**
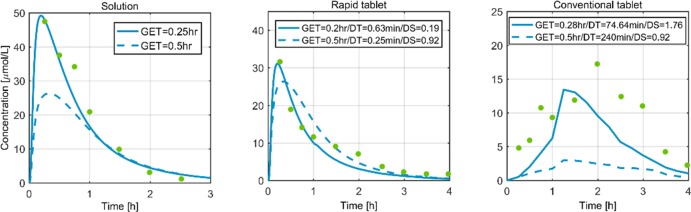
Plasma concentration profiles of three different formulations of 50 mg paracetamol in rabbit. The green dots are data from [38], the dotted lines are the model predictions, and the solid lines represent simulations after parameter adaption. GET: Gastric emptying time (for solution/rapidly disintegrating tablet/conventional tablet). DT: dissolution time (for rapid tablet/conventional tablet). DS: dissolution shape (for rapid tablet/conventional tablet).

For paracetamol, the work of [38] reports three oral formulations: 50 mg of paracetamol administered as an oral solution, a rapidly disintegrating tablet, and a conventional tablet. Fig 4 compares the observed plasma concentration data for the three different oral formulations with the simulation profiles. The dotted lines represent the model predictions based on the typical gastric emptying time (GET = 0.5 hr) of a rabbit and the dissolution shapes (DS). Regarding dissolution time (DT), the rapidly disintegrating tablet model predictions incorporated the values reported in the respective publication [38] (0.25 min). A typical DT (240 min.) was hypothesized for a conventional tablet since no other information was available. For the solution and the rapidly disintegrating tablet, the model predictions (dotted lines, Fig 4 and Table 1) are in close accordance with the observed data. Furthermore, we optimised our model by calibrating the GET, DT, and DS based on the available experimental evidence (solid line, Fig 4). The simulations after parameter calibration explain the data for a solution and a rapidly disintegrating tablet. For the case of a conventional tablet, the data point towards a slower dissolution.

### Predictive performance

Table 1 summarizes the model performance for a) a prediction based on the rabbit model solely informed by literature based values of rabbit physiology and compound related processes, as well as active transport parameters transferred from other species, and b) the simulations after the appropriate parameters were optimized given the observed data. The endpoint was the area under the curve (AUC) of the tissue concentration-time profiles. The table shows the observed, predicted and optimized AUC values along with the respective prediction errors. The mean fold error of the predicted AUC was 1.7, and with the exception of the tablet formulations, the AUC was always below 2. Similar results were obtained when the C_max_ (maximum concentration) predictions and observations were compared (Table N in S1 Appendix).

### Sensitivity analysis

**Fig 5:**
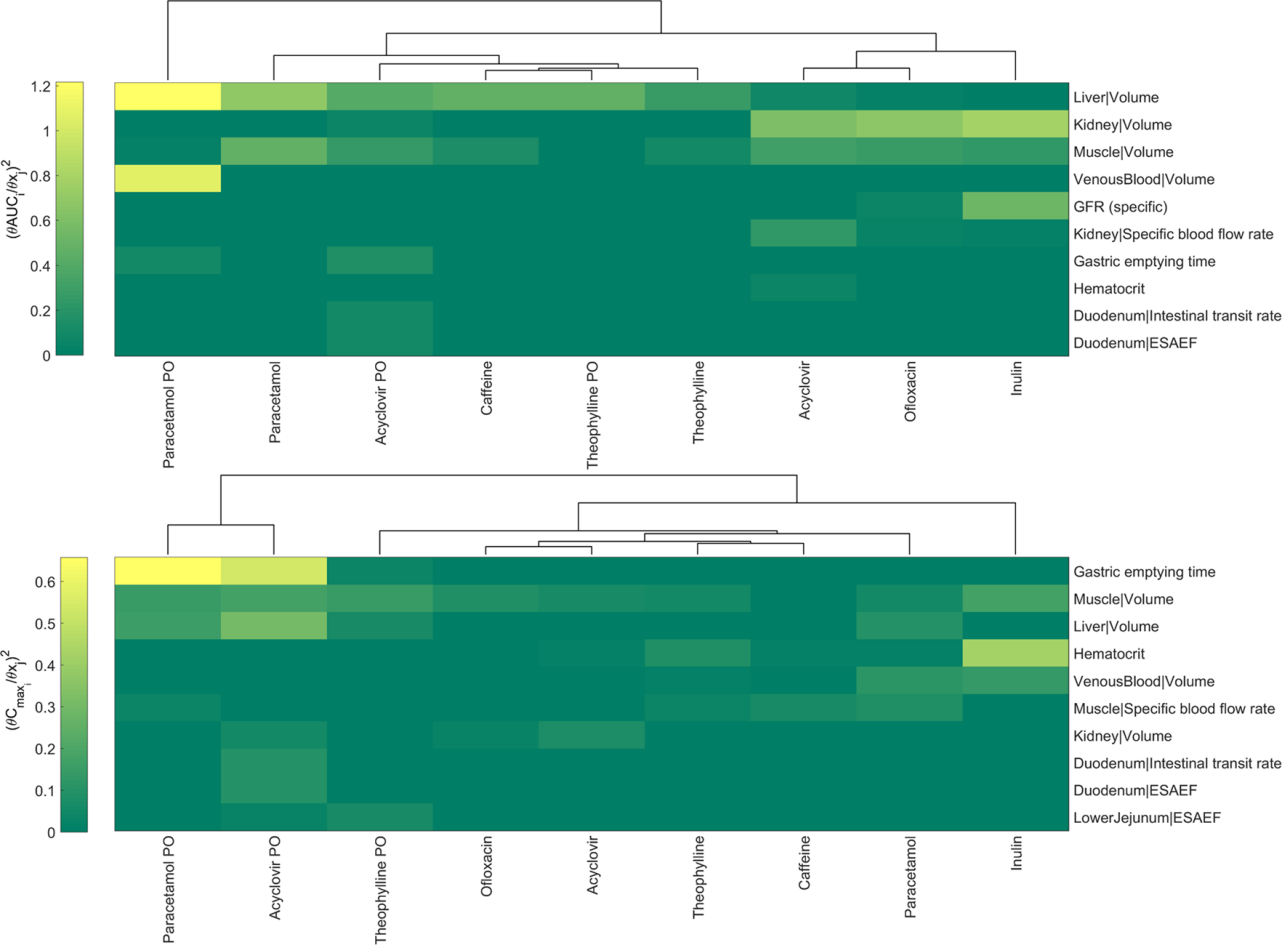
Local sensitivity analysis showing the ten most sensitive parameters for i.v. and p.o. (PO) administration regarding the change in AUC and C_max_. ESAEF stands for Effective Surface Area Enhancement Factor. Upper panel shows sensitivity indices values for AUC variation, and the lower panel sensitivity indices values for C_max_ variation.

A sensitivity analysis was performed to further investigate the relationship between the parameter values and the model output for different compounds and administration strategies (i.v. and p.o. administration). Fig 5 illustrates the relative change in the AUC and C_max_ when the ten most sensitive parameters for each are changed. From green to yellow, the square of the sensitivity coefficient (Eq 1) increases denoting a higher sensitivity of the model output (AUC, C_max_) to the respective parameter. The parameters listed are related to the distribution, clearance or uptake of the substance. For example, inulin is solely cleared by the GFR in the kidney and therefore, the kidney volume and the GFR (specific) are the most sensitive model parameters regarding inulin’s AUC. Similarly, the AUC of caffeine, which is cleared by the liver, remains highly sensitive to the liver volume. On the other hand, C_max_ appears to be more sensitive to variation of parameters that are related to either the route of administration or the distribution of the drugs. For example C_max_ in paracetamol and acyclovir that are administered orally is more sensitive to gastric emptying time whereas in theophylline muscle volume plays a significant role, possibly as relevant volume of distribution. The sensitivity analysis was focused on rabbit-specific parameters and omitted compound specific parameters (e.g. V_max_, K_m_ of active processes) as it is aimed to understand the impact of choosing alternative literature values of the rabbit physiology. An extended version of the sensitivity maps is shown in S11 and S12 Figs.

## Discussion

PBPK/TK modelling is progressively gaining acceptance in risk assessment as it allows for the simulation of tissue-specific compound concentrations and as it is a suitable framework to translate *in vitro* experiments to *in vivo* exposures when considering ADME processes for relevant species [43–45]. In this work, a PBPK/TK model was developed for rabbits, and its predictive capacity was tested based on data from the current literature.

There are a number of reports, opinions and reviews that stress the significance of PBPK/TK modelling in risk assessment and propose the best modelling practices [46–53]. Although there may be deviations in model development strategies based on the questions of interest, the first critical step in PBPK modelling is the review of existing datasets for the relevant species. The results from the rabbit physiology literature search are shown in Tables A-J in S1 Appendix, and the results include information about rabbit organ volumes, blood flow rates, GIT-compartments length/pH/radius/transit times and effective surface areas enhancement factors that represent the various folds, villi and microvilli that ultimately determine the absorption area in the GIT. After informing the model parameter describing the rabbit physiology, the model was validated by testing its predictive capacity on PK data from selected compounds. The validation was conducted to gain more knowledge by either re-estimating the relevant parameters and observing how the predictions improved or by evaluating the sensitivities of the physiological parameters to certain administration scenarios. The rabbit physiology was validated by compounds of gradually increasing complexity regarding clearance, so that any model inconsistency between the simulation and observations could be traced back to the clearance mechanism. For the compounds tested in this work, there were no evidence for different metabolic/transport pathways between rabbit and other species. However, in a PBPK setting, inter-species differences regarding active processes (e.g. an extra transporter) can be considered by re-evaluating compound-related parameters.

The first compound tested was inulin, which is a “gold standard” for measuring GFR [54] and thereby renal function. In our PBPK/TK model, the filtrated amount depends on the fraction unbound (fu) of the compound, the weight of the kidney, and GFR_specific_, which is the volume of the compound filtered per time per weight of the kidney (i.e., l/min/kg organ). These parameters in the rabbit PBPK/TK model were retrieved from the literature and included the deviation of the filtering capacity (GFR_specific_) between individual animals [35]. Fig 3 shows the prediction of our model in dotted lines for six relevant rabbit tissues namely, plasma, lung, skin, bone, heart, and muscle. Interestingly, our model has an exceptionally high predictive capacity even before the experimental data are used to further optimize the model parameters. As Table 1 indicates, the % error of the PBPK/TK model predictions for this experiment is less than 70% (the mean value for all tissues is approximately 35%). To further inform the rabbit PBPK/TK model, additional data from the literature were considered, and the skin’s hydraulic conductivity was recalibrated in accordance with the values observed in rats [36]. Finally, the interstitial fraction of the lungs (i.e., the interstitial volume of the lung over whole tissue volume) was re-estimated to better explain the data. In our panel of compounds, only concentration levels of inulin were observed in other tissues than blood plasma. This data was utilised to adjust parameters of the skin and the lung. As more data in those organs with other compounds become available, those parameters need to be re-evaluated.

In pharmacokinetics/toxicokinetics, there is no *a priori* threshold on an acceptable model error, but PBPK/TK models are generally accepted and considered useful when the prediction error on AUC or C_max_ is in the 2-fold or 3-fold range [55–57]. In the rabbit model presented in this study, a mean fold error of 1.7 was calculated for the AUC among all compounds tested and simulated (Table 1). This result illustrates the high predictive power of our PBPK/TK model in i.v. and p.o. administration scenarios. Specifically, the validation included the kidney function through inulin i.v. administration (Fig 3, S1 Fig), liver function through caffeine i.v. administration (S2 Fig), and simultaneous kidney and liver function through ofloxacin and theophylline i.v. administration (S3–S5 Figs). Additionally, the model was tested on an oral administration of paracetamol through solution, rapidly disintegrating tablet, and conventional tablet formulations. The model predictions for the three formulations are shown in dotted lines in Fig 4. The predictions for the solution and the rapidly disintegrating tablet are in close accordance with the observed data, whereas the ‘conventional’ tablet prediction suggests a significantly different dissolution time (DT). This result is reasonable considering the available experimental data. For the case of solutions, the model assumes an instantaneous availability of the drug in the stomach compartment of the GIT, whereas for both formulations of tablet, a Weibull function was used to empirically describe the tablet dissolution process. For the case of the rapidly disintegrating tablet, the information for the dissolution time found in the published literature (i.e., 0.25 min) [38] was included. However, the dissolution time for the ‘conventional’ tablet formulation of paracetamol was not reported. In practice, dissolution times are experimentally accessible *ex vivo* and could be used in modelling, as done for the rapidly disintegrating tablet. However, the parameters were fit to the data in this study.

As a final step in the PBPK/TK model development, a local sensitivity analysis was performed. A sensitivity analysis is described as a crucial step in the best practices for modelling in risk assessment [47] and is recently used increasingly in numerous mechanism-based modelling efforts in PK/PD [58, 59]. A sensitivity analysis not only evaluates the robustness of the PBPK/TK models but in a PBPK/TK setting where several literature values are available for certain physiologically-based parameters, it reveals whether the choice of alternative values would significantly impact the observed behaviour. The results of the sensitivity analysis output parameters are shown in Fig 5 and S11 Fig for AUC, and Fig 5 and S12 Fig for C_max_. As determined by the change in AUC, the most sensitive parameter was the liver volume followed by the kidney and venous blood volumes. This result is expected since most of the compounds tested were partially or totally cleared through metabolism in the liver. Therefore, a relative change in the liver mass or volume would highly affect the amount of drug cleared and as such the AUC. Similarly, for the compounds cleared renally, either through GFR (inulin) or tubular secretion and GFR (acyclovir), the parameters highly affecting the AUC are kidney volume and filtration capacity (GFR_specific_). The parameters such as gastric emptying time, or GIT-length and the effective surface area are sensitive only in scenarios involving oral administration (i.e., paracetamol PO, acyclovir PO). In contrast, gastric emptying time appears to be the most sensitive parameter regarding C_max_ variation on compounds involving oral administration such as paracetamol and acyclovir. Furthermore, the muscle volume is the largest organ of the rabbit and the most important regarding distribution of drugs in the body hence it appears to be one of the most sensitive parameters. Overall, our analysis reveals sensitivities that are consistent with the biology and clearance pathways of each compound.

The workflow for using the rabbit PBTK model in risk assessment starts with identifying the physicochemical properties of a new active ingredient and its formulation. This step is followed by the use of *in vitro* assays to understand the active transport and metabolism. Ideally, before informing an initial PBTK model, TK data are generated in common species such as mice or rats in addition to the *in vitro* experiments. The translation of the toxicokinetics to the relevant species, such as rabbits and other wild animals, is then an exchange of the physiology-related parameters to the relevant physiology. The method can be used to predict the toxicokinetics of a new substance for different tissues and organs in rabbits. Based on our work, an average fold error of 1.7 can be expected for a compound of typical complexity.

In the present study, we demonstrated the development of a rabbit PBTK model based on data from the available literature. Specifically, a generic rabbit model based on well verified mammalian physiology was further informed by data from the available rabbit-related literature. The model yielded good TK predictions for different compounds with different exposure and clearance routes. By comparing predictions to measured toxicokinetic data, further species-specific parameters could be refined. Overall, this work presents a rabbit PBPK/TK model and its successful validation, and can additionally serve as a blueprint for the development of PBPK/TK models for additional species.

## Supporting information

S1 Fig Simulation of two intravenous administrations of inulin (40 mg/kg blue, 60 mg/kg green) in rabbits weighing 2.85 kg. The solid lines show the simulated venous blood concentration profile and the dots represent the experimental data [37]. No parameter optimization was performed in order to capture inulin’s pharmacokinetic profiles in both doses.
(TIF)

S2 Fig Simulations of venous blood plasma for 4 mg/kg intravenous administration of caffeine in rabbits weighing 3.5 kg. The dotted line shows the simulated profile when no parameters were changed. The solid line shows the simulated profile when the fraction unbound (fu) and Km were adjusted to 0.8 (from 0.7) and 300 (from 400) μmol/L respectively, and the green dots are data adopted from the work of [39].
(TIF)

S3 Fig Simulations of 20 and 40 mg/kg i.v. administration of ofloxacin in rabbits. Venous blood plasma vs. time. The solid lines are the simulations of 20 mg/kg (green) and 40 mg/kg (blue). The dark grey line depicts the fraction excreted in the urine for the small dose. The dots are observed data [60]. Hepatic clearance and tubular secretion were calibrated to 0.5 and 0.1 L/min respectively in order to capture the experimentally observed fraction of ofloxacin excreted in the urine (grey line, 70–90%). The concentration vs time data were then simulated (blue, green lines).
(TIF)

S4 Fig Simulation of venous blood plasma for 12 mg/kg intravenous administration of theophylline in rabbits weighing 2.5 kg. The green dots are the observed data [40]. In order to capture the PK data, fraction unbound was calibrated from 0.8 to 0.5, and liver clearance from 0.07 to 0.04 l/min.
(TIF)

S5 Fig Simulation of venous blood plasma for 15 mg/kg intravenous administration of theophylline in New Zealand white rabbits weighing 3.15 kg and with increasing hepatic clearances from green to grey. The dots are the observed data from the work of [41] for four individual rabbits.
(TIF)

S6 Fig Simulation of venous blood plasma for 200 mg oral administration of theophylline in New Zealand white rabbits weighing 3.15 kg. The dots represent the observed data from the work of [41]. To describe the experimental data the gastric emptying time (GET) was set to 1.66 hr and dissolution time to 10 min which are values inside the ranges found in literature (Table G in S1 Appendix).
(TIF)

S7 Fig Simulation of the venous blood plasma for 35 mg/kg intravenous administration of paracetamol in rabbits weighing 3.17 kg. The green dots are the data adopted from the work of [61]. CYP protein mediated hepatic clearance was parameterised with Vmax = 40 μmol/L/min, Km = 10 μmol/L and considering a liver concentration of the enzyme of 1μmol/L.
(TIF)

S8 Fig Simulation of venous blood plasma for 35 mg/kg intravenous administration of paracetamol in rabbits weighing 3.61 kg. Green dots: observed data [62]. Similar to previous experiment shown in S7 Fig, CYP protein mediated hepatic clearance was parameterised with Vmax = 40 μmol/L/min, Km = 10 μmol/L and considering a liver concentration of the enzyme of 1μmol/L.
(TIF)

S9 Fig PK-Sim® simulation (blue line) and the observed data [42] (green dots) of venous blood plasma profiles of acyclovir, 60 mg/kg i.v. The grey line is the simulated fraction excreted in urine. The relative weights of the renal and hepatic clearances were adjusted to 0.3 L/min to describe the fraction excreted in the urine of 70%. The concentration vs time profile (blue line) was then simulated.
(TIF)

S10 Fig PK-Sim® simulation of venous blood plasma profiles for 300 mg/kg oral administration of acyclovir in rabbits weighing 3.6 kg. The green dots are data adopted from the work of [42]. To describe the data, the gastric emptying time was increased to 4.91 hr from 0.5 hr, which is inside the bounds observed in the literature (Table F in S1 Appendix).
(TIF)

S11 Fig Sensitivity analysis for i.v. and p.o. (PO) administrations regarding the change in AUC. The sensitivity index is defined in Eq 1 of the Materials and Methods section in the main text. ESAEF stands for Effective Surface Area Enhancement Factor.
(TIF)

S12 Fig Sensitivity analysis for i.v. and p.o. (PO) administrations regarding the change in Cmax. The sensitivity index is defined in Eq 1 of the Materials and Methods section in the main text. ESAEF stands for Effective Surface Area Enhancement Factor.
(TIF)

S1 Appendix Details of the rabbit physiology from the literature and model parameter. Rabbit PBPK model validation with Inulin, Caffeine, Ofloxacin, Theophylline, Paracetamol, and Acyclovir.
(DOCX)

## References

[1] Russell WMS, Burch RL. The principles of humane experimental technique. The principles of humane experimental technique. 1959.

[2] Hartung T. Food for thought… on animal tests. ALTEX. 2008;25(1):3–16. . 18360722 10.14573/altex.2008.1.3

[3] Leist M, Hartung T, Nicotera P. The dawning of a new age of toxicology. ALTEX. 2008;25(2):103–14. . 18551234

[4] Scholz S, Sela E, Blaha L, Braunbeck T, Galay-Burgos M, Garcia-Franco M, et al A European perspective on alternatives to animal testing for environmental hazard identification and risk assessment. Regul Toxicol Pharmacol. 2013;67(3):506–30. doi: 10.1016/j.yrtph.2013.10.003 . 24161465 10.1016/j.yrtph.2013.10.003

[5] Lilienblum W, Dekant W, Foth H, Gebel T, Hengstler JG, Kahl R, et al Alternative methods to safety studies in experimental animals: role in the risk assessment of chemicals under the new European Chemicals Legislation (REACH). Arch Toxicol. 2008;82(4):211–36. doi: 10.1007/s00204-008-0279-9 . 18322675 10.1007/s00204-008-0279-9

[6] ECHA. European Chemicals Agency. The Use of Alternatives to Testing on Animals for the REACH Regulation 2011. Helsinki,Finland.: 2011.

[7] Eissing T, Kuepfer L, Becker C, Block M, Coboeken K, Gaub T, et al A computational systems biology software platform for multiscale modeling and simulation: integrating whole-body physiology, disease biology, and molecular reaction networks. Frontiers in physiology. 2011;2:4 doi: 10.3389/fphys.2011.00004 ; PubMed Central PMCID: PMC3070480. 21483730 10.3389/fphys.2011.00004 PMC3070480

[8] Jones HM, Chen Y, Gibson C, Heimbach T, Parrott N, Peters SA, et al Physiologically based pharmacokinetic modeling in drug discovery and development: a pharmaceutical industry perspective. Clin Pharmacol Ther. 2015;97(3):247–62. doi: 10.1002/cpt.37 . 25670209 10.1002/cpt.37

[9] Reddy M, Yang R, Andersen ME, Clewell HJ III III. Physiologically based pharmacokinetic modeling: science and applications: John Wiley & Sons; 2005.

[10] Edginton AN, Theil FP, Schmitt W, Willmann S. Whole body physiologically-based pharmacokinetic models: their use in clinical drug development. Expert opinion on drug metabolism & toxicology. 2008;4(9):1143–52. doi: 10.1517/17425255.4.9.1143 . 18721109 10.1517/17425255.4.9.1143

[11] Andersen ME. Toxicokinetic modeling and its applications in chemical risk assessment. Toxicology letters. 2003;138(1–2):9–27. . 12559690 10.1016/s0378-4274(02)00375-2

[12] Jusko WJ. Moving from basic toward systems pharmacodynamic models. J Pharm Sci. 2013;102(9):2930–40. doi: 10.1002/jps.23590 ; PubMed Central PMCID: PMC3743951. 23681608 10.1002/jps.23590 PMC3743951

[13] Thiel C, Schneckener S, Krauss M, Ghallab A, Hofmann U, Kanacher T, et al A systematic evaluation of the use of physiologically based pharmacokinetic modeling for cross-species extrapolation. J Pharm Sci. 2015;104(1):191–206. doi: 10.1002/jps.24214 . 25393841 10.1002/jps.24214

[14] Sager JE, Yu J, Ragueneau-Majlessi I, Isoherranen N. Physiologically Based Pharmacokinetic (PBPK) Modeling and Simulation Approaches: A Systematic Review of Published Models, Applications, and Model Verification. Drug Metab Dispos. 2015;43(11):1823–37. doi: 10.1124/dmd.115.065920 ; PubMed Central PMCID: PMC4613950. 26296709 10.1124/dmd.115.065920 PMC4613950

[15] Zhuang X, Lu C. PBPK modeling and simulation in drug research and development. Acta Pharm Sin B. 2016;6(5):430–40. doi: 10.1016/j.apsb.2016.04.004 ; PubMed Central PMCID: PMC5125732. 27909650 10.1016/j.apsb.2016.04.004 PMC5125732

[16] Zhao P, Zhang L, Grillo JA, Liu Q, Bullock JM, Moon YJ, et al Applications of physiologically based pharmacokinetic (PBPK) modeling and simulation during regulatory review. Clin Pharmacol Ther. 2011;89(2):259–67. doi: 10.1038/clpt.2010.298 . 21191381 10.1038/clpt.2010.298

[17] European Food Safety A. Modern methodologies and tools for human hazard assessment of chemicals. EFSA Journal. 2014;12(4):3638-n/a. doi: 10.2903/j.efsa.2014.3638

[18] Meek ME, Barton HA, Bessems JG, Lipscomb JC, Krishnan K. Case study illustrating the WHO IPCS guidance on characterization and application of physiologically based pharmacokinetic models in risk assessment. Regul Toxicol Pharmacol. 2013;66(1):116–29. doi: 10.1016/j.yrtph.2013.03.005 . 23535119 10.1016/j.yrtph.2013.03.005

[19] Hillisch A, Heinrich N, Wild H. Computational Chemistry in the Pharmaceutical Industry: From Childhood to Adolescence. ChemMedChem. 2015;10(12):1958–62. doi: 10.1002/cmdc.201500346 . 26358802 10.1002/cmdc.201500346

[20] Willmann S, Lippert J, Sevestre M, Solodenko J, Fois F, Schmitt W. PK-Sim®: a physiologically based pharmacokinetic ‘whole-body’ model. BIOSILICO. 2003;1(4):121–4. http://dx.doi.org/10.1016/S1478-5382(03)02342-4.

[21] Willmann S, Schmitt W, Keldenich J, Dressman JB. A physiologic model for simulating gastrointestinal flow and drug absorption in rats. Pharm Res. 2003;20(11):1766–71. . 14661920 10.1023/b:pham.0000003373.72652.c0

[22] Willmann S, Schmitt W, Keldenich J, Lippert J, Dressman JB. A physiological model for the estimation of the fraction dose absorbed in humans. J Med Chem. 2004;47(16):4022–31. doi: 10.1021/jm030999b . 15267240 10.1021/jm030999b

[23] Willmann S, Lippert J, Schmitt W. From physicochemistry to absorption and distribution: predictive mechanistic modelling and computational tools. Expert opinion on drug metabolism & toxicology. 2005;1(1):159–68. doi: 10.1517/17425255.1.1.159 . 16922658 10.1517/17425255.1.1.159

[24] Willmann S, Hohn K, Edginton A, Sevestre M, Solodenko J, Weiss W, et al Development of a physiology-based whole-body population model for assessing the influence of individual variability on the pharmacokinetics of drugs. J Pharmacokinet Pharmacodyn. 2007;34(3):401–31. Epub 2007/04/14. doi: 10.1007/s10928-007-9053-5 . 17431751 10.1007/s10928-007-9053-5

[25] Thelen K, Coboeken K, Willmann S, Burghaus R, Dressman JB, Lippert J. Evolution of a detailed physiological model to simulate the gastrointestinal transit and absorption process in humans, part 1: oral solutions. J Pharm Sci. 2011;100(12):5324–45. Epub 2011/10/14. doi: 10.1002/jps.22726 . 21993815 10.1002/jps.22726

[26] Schmitt W, Willmann S. Physiology-based pharmacokinetic modeling: ready to be used. Drug discovery today Technologies. 2005;2(1):125–32. doi: 10.1016/j.ddtec.2005.01.001 . 24981765 10.1016/j.ddtec.2005.01.001

[27] Davies B, Morris T. Physiological parameters in laboratory animals and humans. Pharm Res. 1993;10(7):1093–5. . 8378254 10.1023/a:1018943613122

[28] Kararli TT. Comparison of the gastrointestinal anatomy, physiology, and biochemistry of humans and commonly used laboratory animals. Biopharm Drug Dispos. 1995;16(5):351–80. . 8527686 10.1002/bdd.2510160502

[29] Lebas F, Coudert P, de Rochambeau H, Thébault R. The Rabbit: Husbandry, health and production FAO Animal Production and Health Series No 21. Rome: Food and agriculture organization of the United Nations; 1997.

[30] Willmann S, Lippert J, Sevestre M, Solodenko J, Fois F, Schmitt W. PK-Sim®: a physiologically based pharmacokinetic ‘whole-body’model. Biosilico. 2003;1(4):121–4.

[31] Poulin P, Theil FP. A priori prediction of tissue:plasma partition coefficients of drugs to facilitate the use of physiologically-based pharmacokinetic models in drug discovery. J Pharm Sci. 2000;89(1):16–35. doi: 10.1002/(SICI)1520-6017(200001)89:1<16::AID-JPS3>3.0.CO;2-E . 10664535 10.1002/(SICI)1520-6017(200001)89:1<16::AID-JPS3>3.0.CO;2-E

[32] Prescott LF, Nimmo WS. Novel drug delivery and its therapeutic application: Wiley; 1989.

[33] Ferl GZ, Wu AM, DiStefano JJ, 3rd. A predictive model of therapeutic monoclonal antibody dynamics and regulation by the neonatal Fc receptor (FcRn). Ann Biomed Eng. 2005;33(11):1640–52. doi: 10.1007/s10439-005-7410-3 . 16341929 10.1007/s10439-005-7410-3

[34] Tsuji A, Nishide K, Minami H, Nakashima E, Terasaki T, Yamana T. Physiologically based pharmacokinetic model for cefazolin in rabbits and its preliminary extrapolation to man. Drug metabolism and disposition: the biological fate of chemicals. 1985;13(6):729–39. . 2867880

[35] Michigoshi Y, Katayama R, Yamagishi N, Kato M, Saito J, Satoh H, et al Estimation of glomerular filtration rate in rabbits by a single-sample method using iodixanol. Lab Anim. 2012;46(4):341–4. doi: 10.1258/la.2012.011065 . 23097569 10.1258/la.2012.011065

[36] Renkin EM, Gustafson-Sgro M, Sibley L. Coupling of albumin flux to volume flow in skin and muscles of anesthetized rats. The American journal of physiology. 1988;255(3 Pt 2):H458–66. doi: 10.1152/ajpheart.1988.255.3.H458 . 3414813 10.1152/ajpheart.1988.255.3.H458

[37] Michigoshi Y, Yamagishi N, Satoh H, Kato M, Furuhama K. Using a single blood sample and inulin to estimate glomerular filtration rate in rabbits. Journal of the American Association for Laboratory Animal Science: JAALAS. 2011;50(5):702–7. ; PubMed Central PMCID: PMC3189675. 22330718 PMC3189675

[38] Ishikawa T, Koizumi N, Mukai B, Utoguchi N, Fujii M, Matsumoto M, et al Pharmacokinetics of acetaminophen from rapidly disintegrating compressed tablet prepared using microcrystalline cellulose (PH-M-06) and spherical sugar granules. Chemical & pharmaceutical bulletin. 2001;49(2):230–2. . 11217114 10.1248/cpb.49.230

[39] Beach CA, Mays DC, Sterman BM, Gerber N. Metabolism, distribution, seminal excretion and pharmacokinetics of caffeine in the rabbit. The Journal of pharmacology and experimental therapeutics. 1985;233(1):18–23. . 3981454

[40] Celardo A, Traina GL, Jankowski A, Bonati M. Pharmacokinetics of theophylline and its metabolites in rabbits. European journal of drug metabolism and pharmacokinetics. 1985;10(4):279–88. . 3830715 10.1007/BF03189755

[41] El-Yazigi A, Sawchuk RJ. Theophylline absorption and disposition in rabbits: oral, intravenous, and concentration-dependent kinetic studies. J Pharm Sci. 1981;70(4):452–6. . 7229966 10.1002/jps.2600700429

[42] van Jaarsveld MF, Walubo A, du Plessis JB. Interaction between valproic acid and acyclovir after intravenous and oral administration in a rabbit model. Basic & clinical pharmacology & toxicology. 2007;101(6):434–40. doi: 10.1111/j.1742-7843.2007.00134.x . 18028106 10.1111/j.1742-7843.2007.00134.x

[43] Wiltse JA, Dellarco VL. U.S. Environmental Protection Agency's revised guidelines for carcinogen risk assessment: evaluating a postulated mode of carcinogenic action in guiding dose-response extrapolation. Mutation research. 2000;464(1):105–15. . 10633182 10.1016/s1383-5718(99)00171-0

[44] Chiu WA, Barton HA, DeWoskin RS, Schlosser P, Thompson CM, Sonawane B, et al Evaluation of physiologically based pharmacokinetic models for use in risk assessment. J Appl Toxicol. 2007;27(3):218–37. Epub 2007/02/15. doi: 10.1002/jat.1225 . 17299829 10.1002/jat.1225

[45] Bessems JG, Loizou G, Krishnan K, Clewell HJ, 3rd, Bernasconi C, Bois F, et al PBTK modelling platforms and parameter estimation tools to enable animal-free risk assessment: recommendations from a joint EPAA—EURL ECVAM ADME workshop. Regulatory toxicology and pharmacology: RTP. 2014;68(1):119–39. doi: 10.1016/j.yrtph.2013.11.008 . 24287156 10.1016/j.yrtph.2013.11.008

[46] Lipscomb JC, Haddad S, Poet T, Krishnan K. Physiologically-based pharmacokinetic (PBPK) models in toxicity testing and risk assessment. Advances in experimental medicine and biology. 2012;745:76–95. doi: 10.1007/978-1-4614-3055-1_6 . 22437814 10.1007/978-1-4614-3055-1_6

[47] Loizou G, Spendiff M, Barton HA, Bessems J, Bois FY, d'Yvoire MB, et al Development of good modelling practice for physiologically based pharmacokinetic models for use in risk assessment: the first steps. Regulatory toxicology and pharmacology: RTP. 2008;50(3):400–11. doi: 10.1016/j.yrtph.2008.01.011 . 18331772 10.1016/j.yrtph.2008.01.011

[48] Louisse J, Bosgra S, Blaauboer BJ, Rietjens IM, Verwei M. Prediction of in vivo developmental toxicity of all-trans-retinoic acid based on in vitro toxicity data and in silico physiologically based kinetic modeling. Archives of toxicology. 2015;89(7):1135–48. doi: 10.1007/s00204-014-1289-4 . 24935252 10.1007/s00204-014-1289-4

[49] Mumtaz M, Fisher J, Blount B, Ruiz P. Application of physiologically based pharmacokinetic models in chemical risk assessment. Journal of toxicology. 2012;2012:904603 doi: 10.1155/2012/904603 ; PubMed Central PMCID: PMC3317240. 22523493 10.1155/2012/904603 PMC3317240

[50] Hartmanshenn C, Scherholz M, Androulakis IP. Physiologically-based pharmacokinetic models: approaches for enabling personalized medicine. J Pharmacokinet Pharmacodyn. 2016;43(5):481–504. doi: 10.1007/s10928-016-9492-y . 27647273 10.1007/s10928-016-9492-y PMC5204363

[51] Androulakis IP. Quantitative Systems Pharmacology: A Framework for Context. Curr Pharmacol Rep. 2016;2(3):152–60. doi: 10.1007/s40495-016-0058-x ; PubMed Central PMCID: PMCPMC4996481. 27570730 10.1007/s40495-016-0058-x PMC4996481

[52] Block M. Physiologically based pharmacokinetic and pharmacodynamic modeling in cancer drug development: status, potential and gaps. Expert Opin Drug Metab Toxicol. 2015;11(5):743–56. doi: 10.1517/17425255.2015.1037276 . 25940026 10.1517/17425255.2015.1037276

[53] Mager DE, Jusko WJ. Development of translational pharmacokinetic-pharmacodynamic models. Clin Pharmacol Ther. 2008;83(6):909–12. doi: 10.1038/clpt.2008.52 ; PubMed Central PMCID: PMCPMC2671003. 18388873 10.1038/clpt.2008.52 PMC2671003

[54] Jones GRD, Lim E-M. The National Kidney Foundation Guideline on Estimation of the Glomerular Filtration Rate. The Clinical Biochemist Reviews. 2003;24(3):95–8. PMC1853341.

[55] Maharaj AR, Edginton AN. Physiologically based pharmacokinetic modeling and simulation in pediatric drug development. CPT Pharmacometrics Syst Pharmacol. 2014;3:e150 doi: 10.1038/psp.2014.45 ; PubMed Central PMCID: PMC4260000. 25353188 10.1038/psp.2014.45 PMC4260000

[56] Poulin P, Jones RD, Jones HM, Gibson CR, Rowland M, Chien JY, et al PHRMA CPCDC initiative on predictive models of human pharmacokinetics, part 5: prediction of plasma concentration-time profiles in human by using the physiologically-based pharmacokinetic modeling approach. J Pharm Sci. 2011;100(10):4127–57. doi: 10.1002/jps.22550 . 21541937 10.1002/jps.22550

[57] De Buck SS, Sinha VK, Fenu LA, Nijsen MJ, Mackie CE, Gilissen RA. Prediction of human pharmacokinetics using physiologically based modeling: a retrospective analysis of 26 clinically tested drugs. Drug metabolism and disposition: the biological fate of chemicals. 2007;35(10):1766–80. doi: 10.1124/dmd.107.015644 . 17620347 10.1124/dmd.107.015644

[58] Hartmann S, Biliouris K, Lesko LJ, Nowak-Gottl U, Trame MN. Quantitative Systems Pharmacology Model to Predict the Effects of Commonly Used Anticoagulants on the Human Coagulation Network. CPT Pharmacometrics Syst Pharmacol. 2016;5(10):554–64. doi: 10.1002/psp4.12111 ; PubMed Central PMCID: PMCPMC5080651. 27647667 10.1002/psp4.12111 PMC5080651

[59] Zhang L, Mager DE. Physiologically-based pharmacokinetic modeling of target-mediated drug disposition of bortezomib in mice. J Pharmacokinet Pharmacodyn. 2015;42(5):541–52. doi: 10.1007/s10928-015-9445-x ; PubMed Central PMCID: PMCPMC4620045. 26391023 10.1007/s10928-015-9445-x PMC4620045

[60] Marangos MN, Zhu Z, Nicolau DP, Klepser ME, Nightingale CH. Disposition of ofloxacin in female New Zealand white rabbits. Journal of veterinary pharmacology and therapeutics. 1997;20(1):17–20. . 9049944 10.1046/j.1365-2885.1997.00812.x

[61] Karbownik A, Szalek E, Sobanska K, Polom W, Grabowski T, Biczysko-Murawa A, et al The effect of sunitinib on the plasma exposure of intravenous paracetamol and its major metabolite: paracetamol glucuronide. European journal of drug metabolism and pharmacokinetics. 2015;40(2):163–70. doi: 10.1007/s13318-014-0191-z ; PubMed Central PMCID: PMC4426134. 24676873 10.1007/s13318-014-0191-z PMC4426134

[62] Bienert A, Kaminska A, Olszewski J, Gracz J, Grabowski T, Wolc A, et al Pharmacokinetics and ocular disposition of paracetamol and paracetamol glucuronide in rabbits with diabetes mellitus induced by alloxan. Pharmacological reports: PR. 2012;64(2):421–7. . 22661194 10.1016/s1734-1140(12)70783-1

