# Supplementary figures and images for: Development and validation of a physiology-based model for the prediction of pharmacokinetics/toxicokinetics in rabbits
Source: PLoS One. 2018 Mar 21;13(3):e0194294. doi: 10.1371/journal.pone.0194294 (PMC5862475; doi:10.1371/journal.pone.0194294)

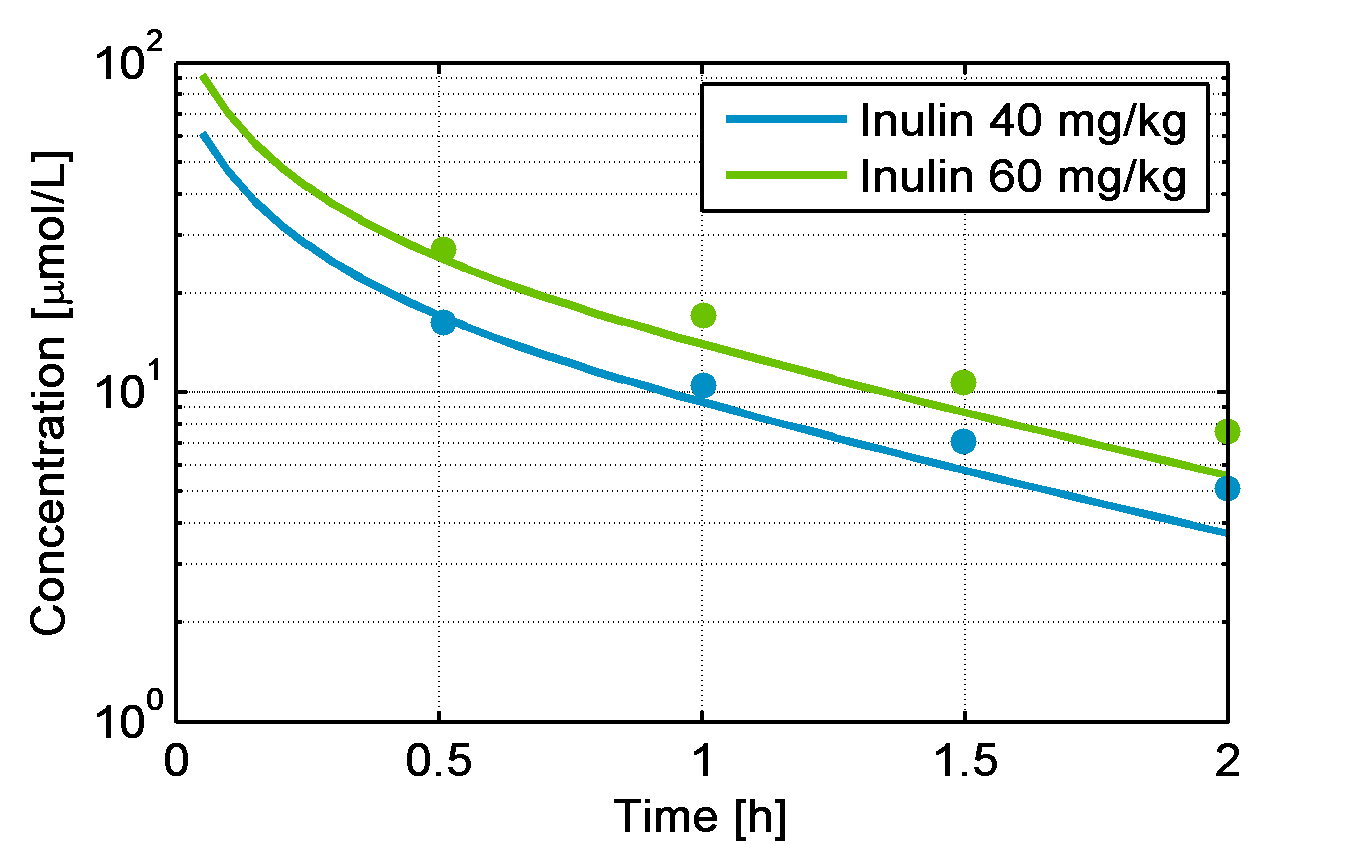

Supplement: S1 Fig — The solid lines show the simulated venous blood concentration profile and the dots represent the experimental data [37]. No parameter optimization was performed in order to capture inulin’s pharmacokinetic profiles in both doses. (TIF) [file pone.0194294.s001.tif]

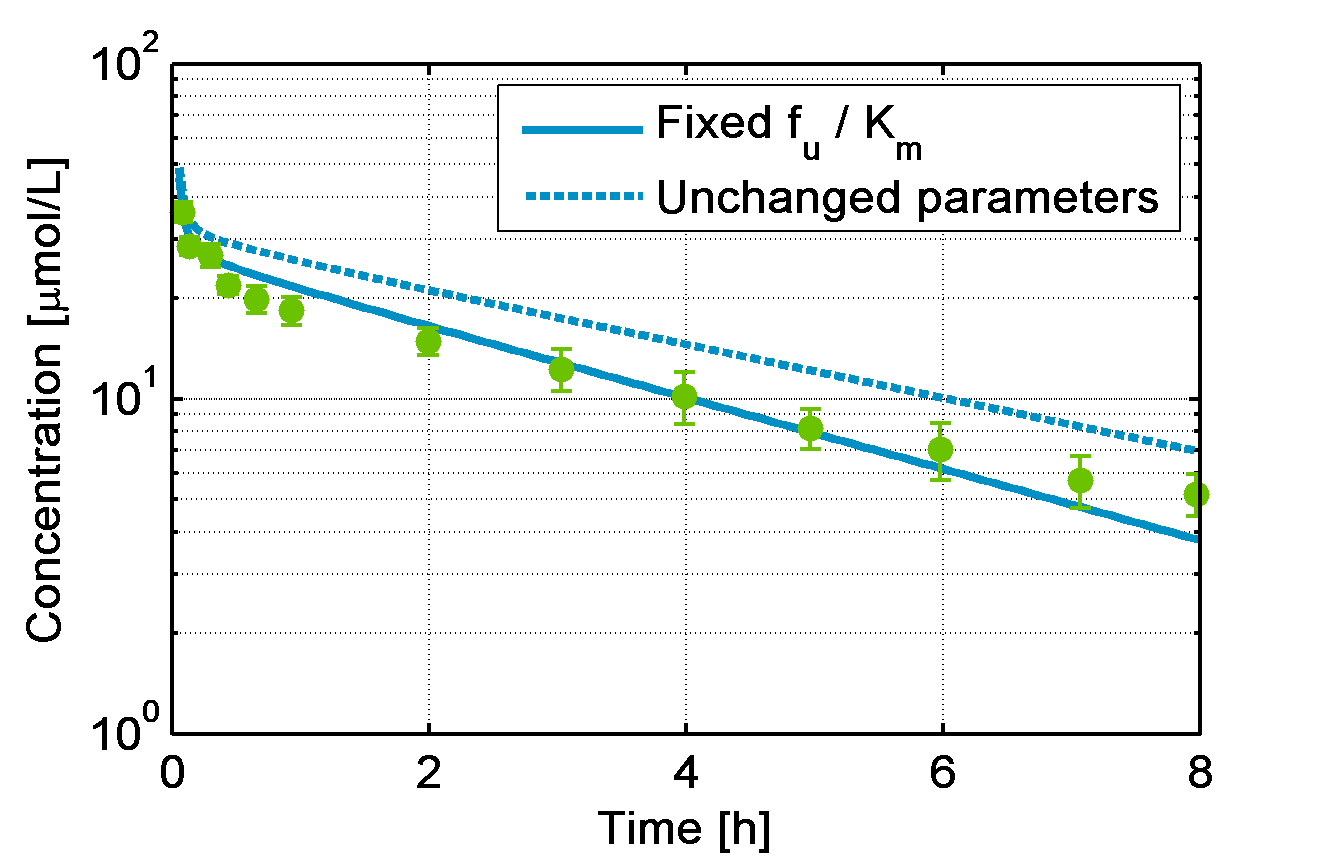

Supplement: S2 Fig — The dotted line shows the simulated profile when no parameters were changed. The solid line shows the simulated profile when the fraction unbound (fu) and Km were adjusted to 0.8 (from 0.7) and 300 (from 400) μmol/L respectively, and the green dots are data adopted from the work of [39]. (TIF) [file pone.0194294.s002.tif]

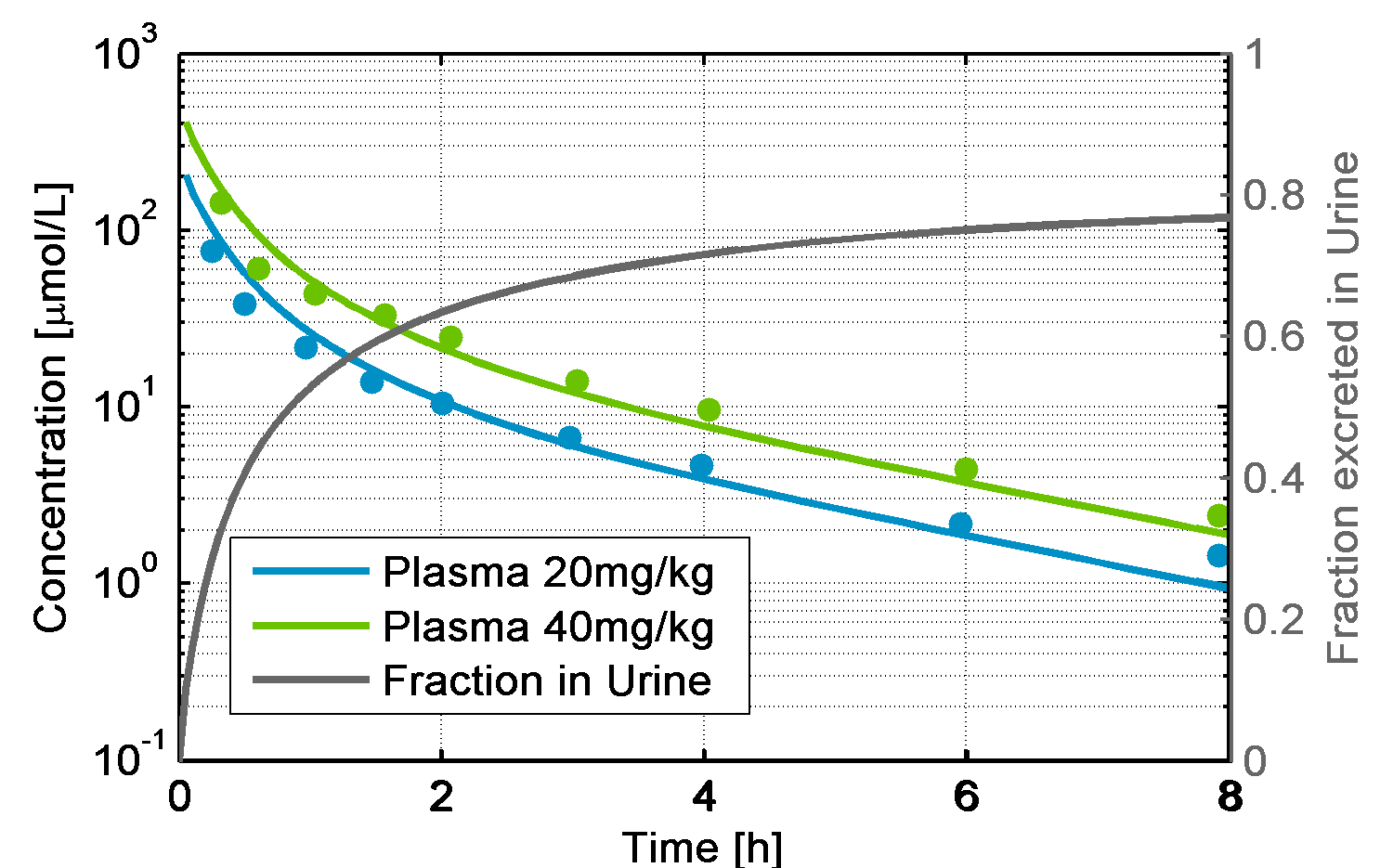

Supplement: S3 Fig — Venous blood plasma vs. time. The solid lines are the simulations of 20 mg/kg (green) and 40 mg/kg (blue). The dark grey line depicts the fraction excreted in the urine for the small dose. The dots are observed data [60]. Hepatic clearance and tubular secretion were calibrated to 0.5 and 0.1 L/min respectively in order to capture the experimentally observed fraction of ofloxacin excreted in the urine (grey line, 70–90%). The concentration vs time data were then simulated (blue, green lines). (TIF) [file pone.0194294.s003.tif]

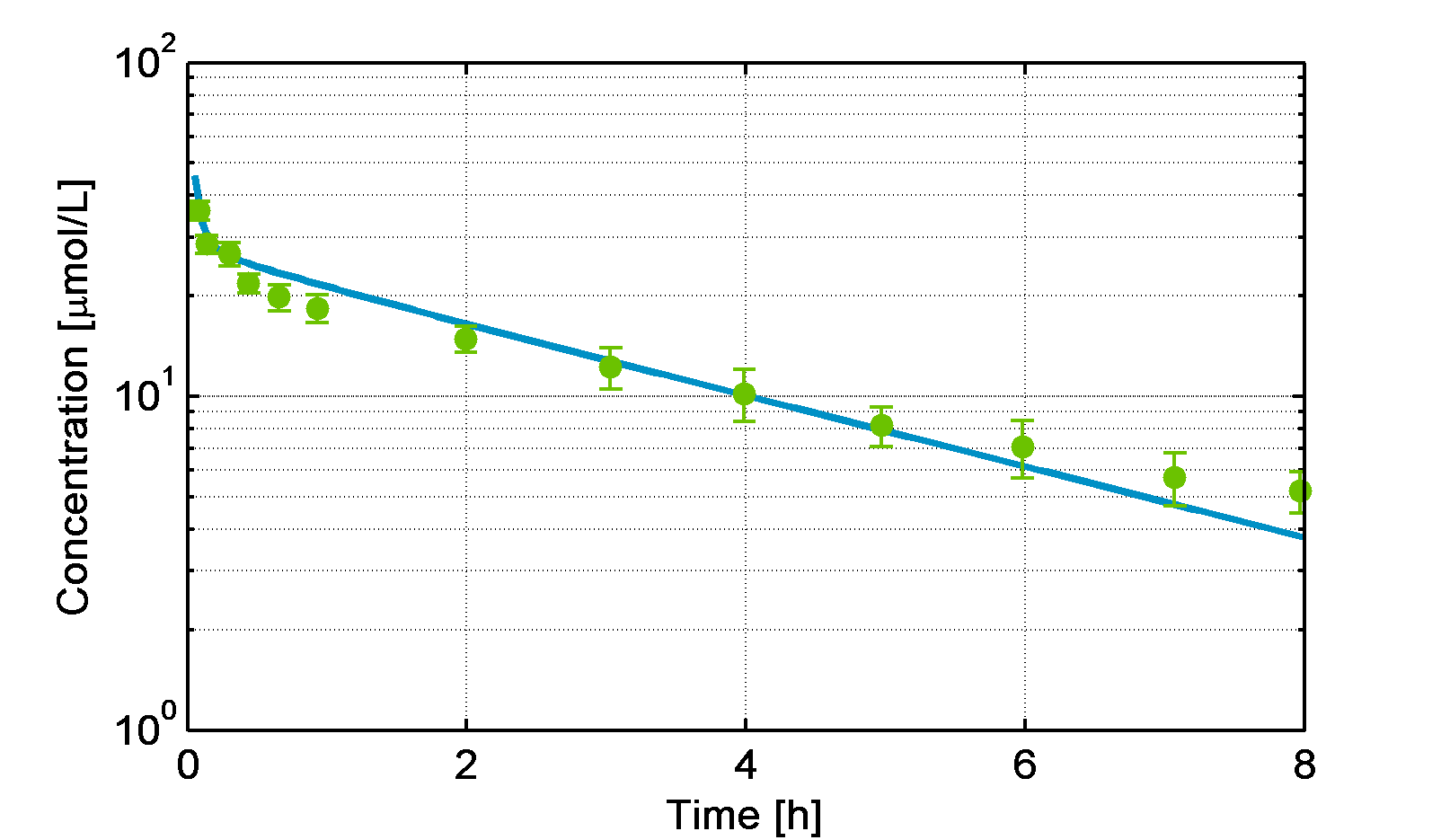

Supplement: S4 Fig — The green dots are the observed data [40]. In order to capture the PK data, fraction unbound was calibrated from 0.8 to 0.5, and liver clearance from 0.07 to 0.04 l/min. (TIF) [file pone.0194294.s004.tif]

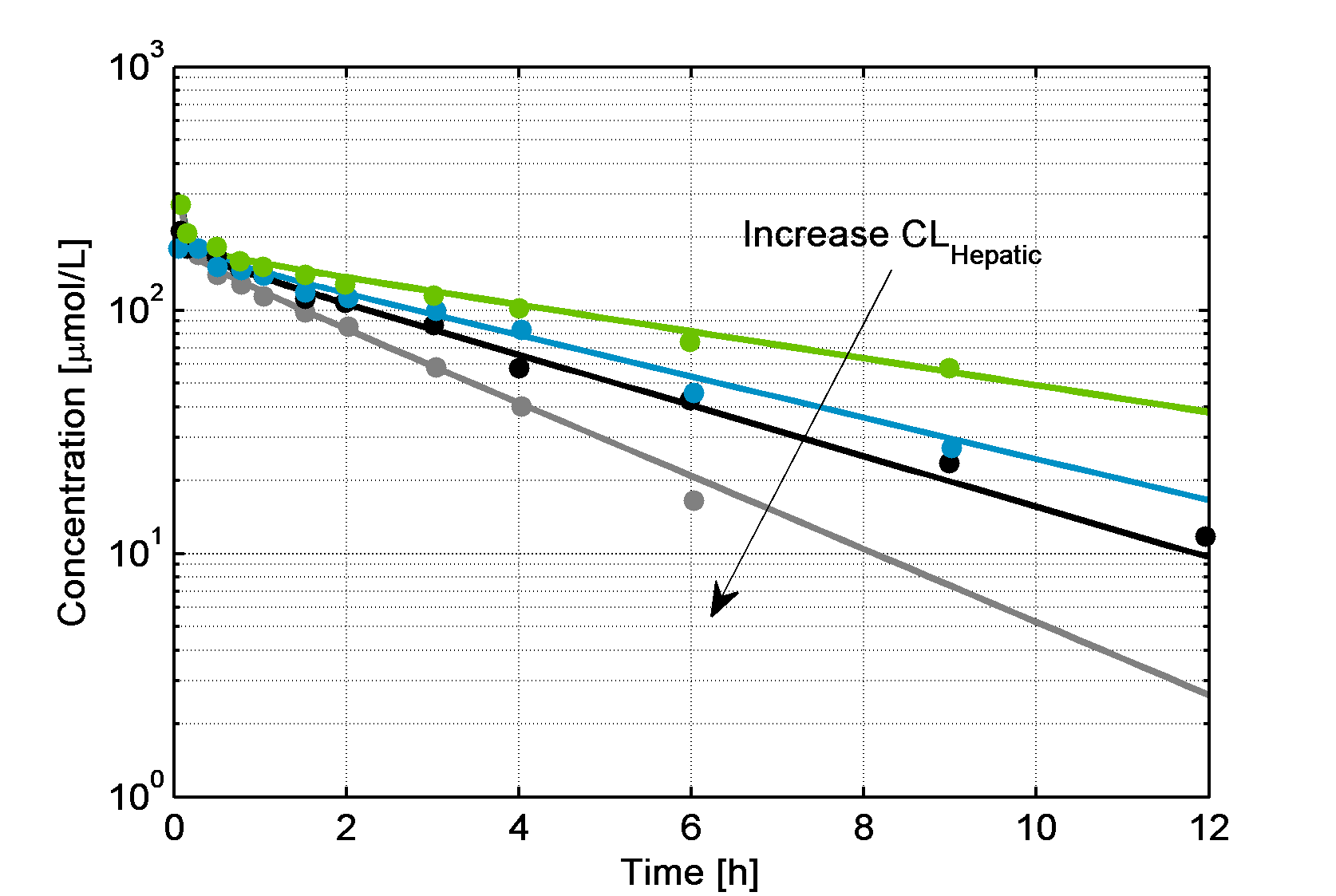

Supplement: S5 Fig — The dots are the observed data from the work of [41] for four individual rabbits. (TIF) [file pone.0194294.s005.tif]

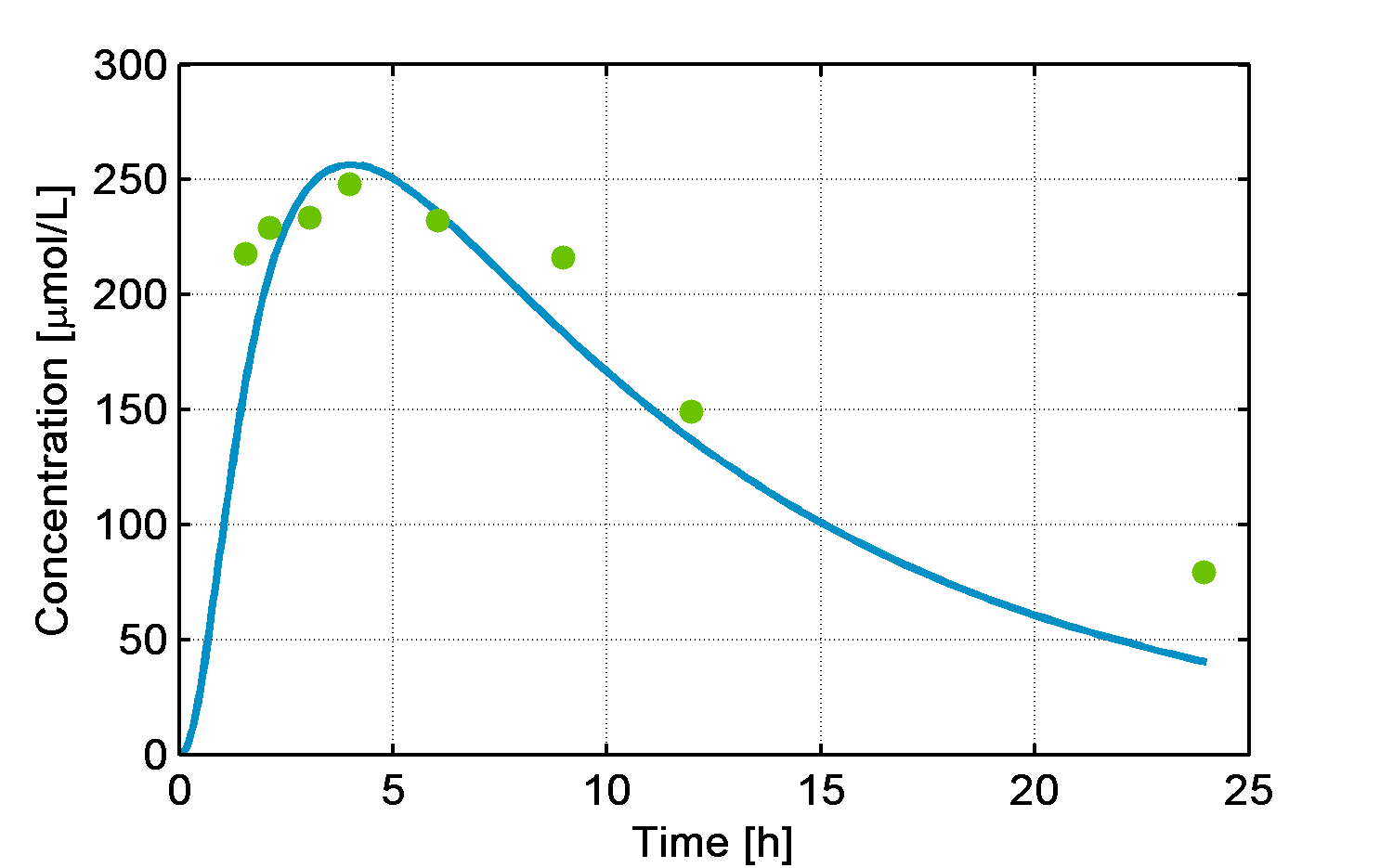

Supplement: S6 Fig — The dots represent the observed data from the work of [41]. To describe the experimental data the gastric emptying time (GET) was set to 1.66 hr and dissolution time to 10 min which are values inside the ranges found in literature (Table G in S1 Appendix). (TIF) [file pone.0194294.s006.tif]

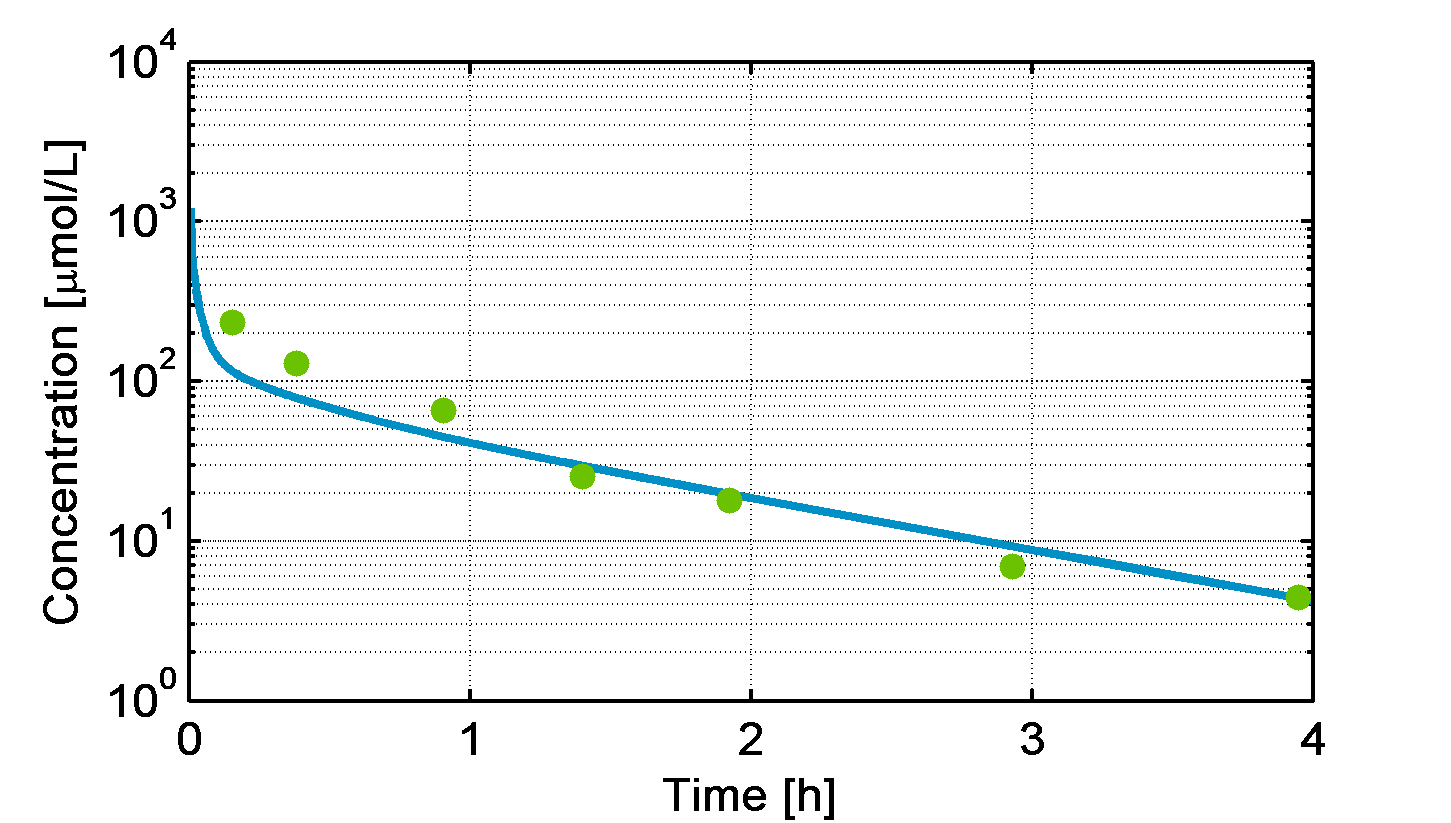

Supplement: S7 Fig — The green dots are the data adopted from the work of [61]. CYP protein mediated hepatic clearance was parameterised with Vmax = 40 μmol/L/min, Km = 10 μmol/L and considering a liver concentration of the enzyme of 1μmol/L. (TIF) [file pone.0194294.s007.tif]

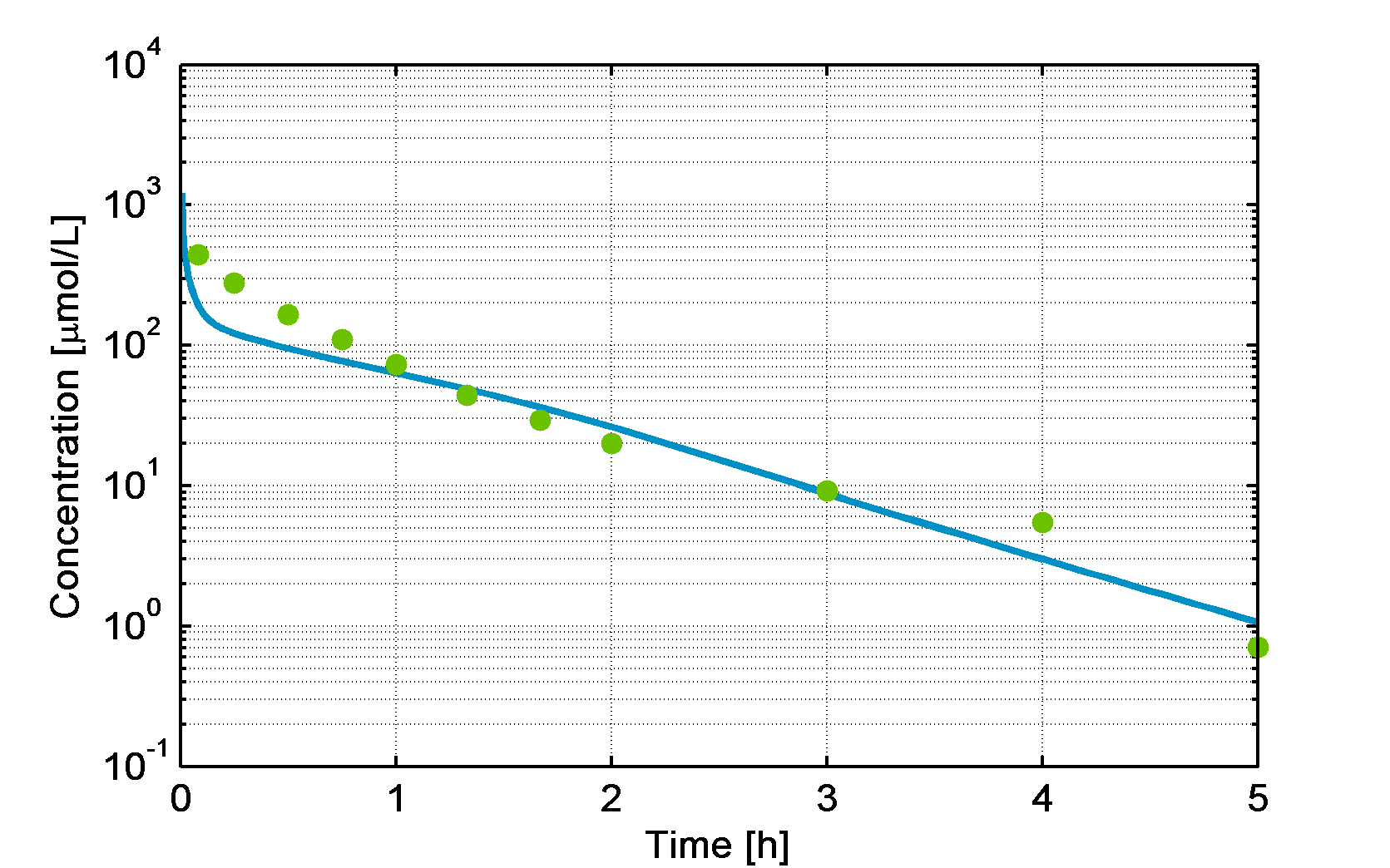

Supplement: S8 Fig — Green dots: observed data [62]. Similar to previous experiment shown in S7 Fig, CYP protein mediated hepatic clearance was parameterised with Vmax = 40 μmol/L/min, Km = 10 μmol/L and considering a liver concentration of the enzyme of 1μmol/L. (TIF) [file pone.0194294.s008.tif]

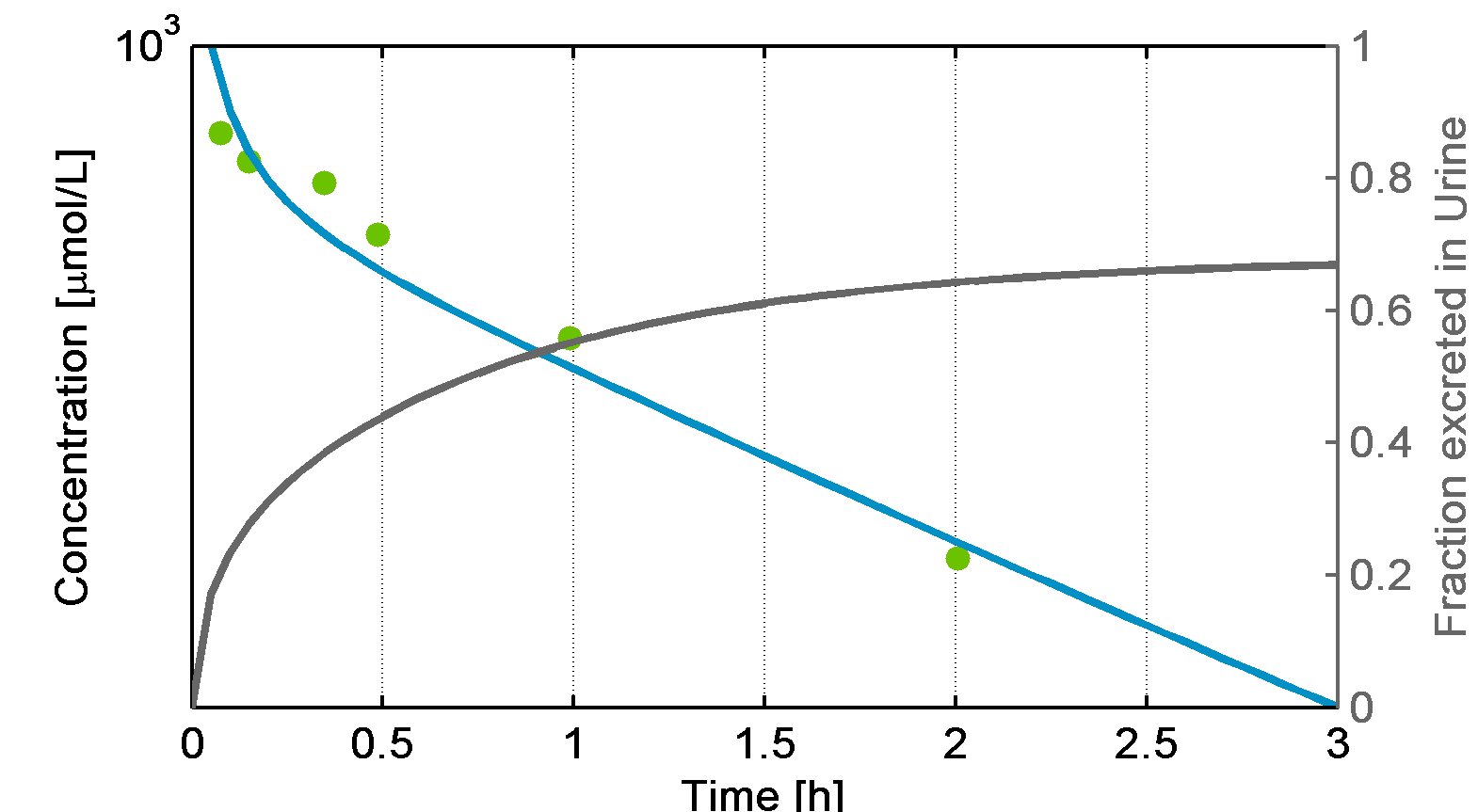

Supplement: S9 Fig — The grey line is the simulated fraction excreted in urine. The relative weights of the renal and hepatic clearances were adjusted to 0.3 L/min to describe the fraction excreted in the urine of 70%. The concentration vs time profile (blue line) was then simulated. (TIF) [file pone.0194294.s009.tif]

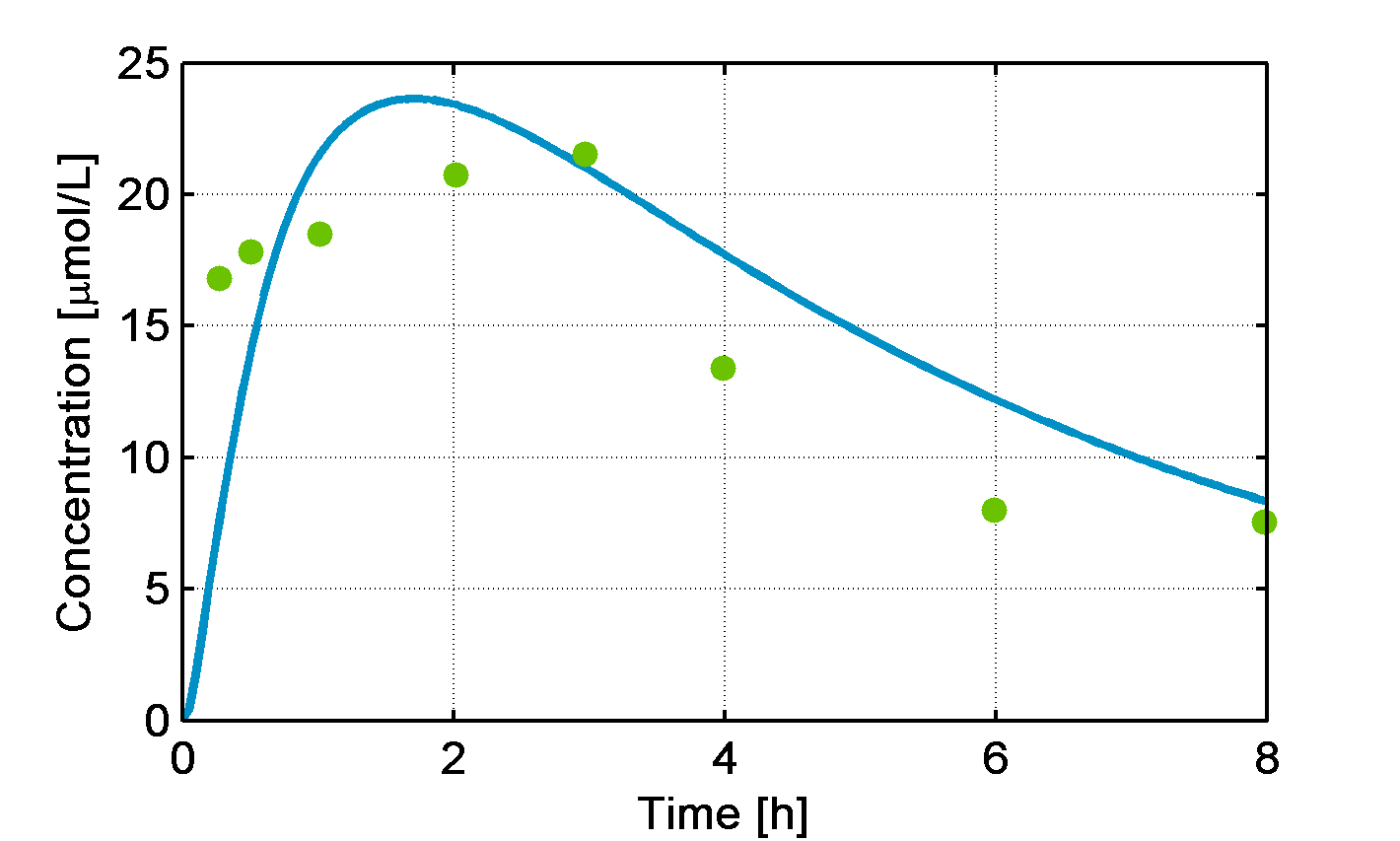

Supplement: S10 Fig — The green dots are data adopted from the work of [42]. To describe the data, the gastric emptying time was increased to 4.91 hr from 0.5 hr, which is inside the bounds observed in the literature (Table F in S1 Appendix). (TIF) [file pone.0194294.s010.tif]

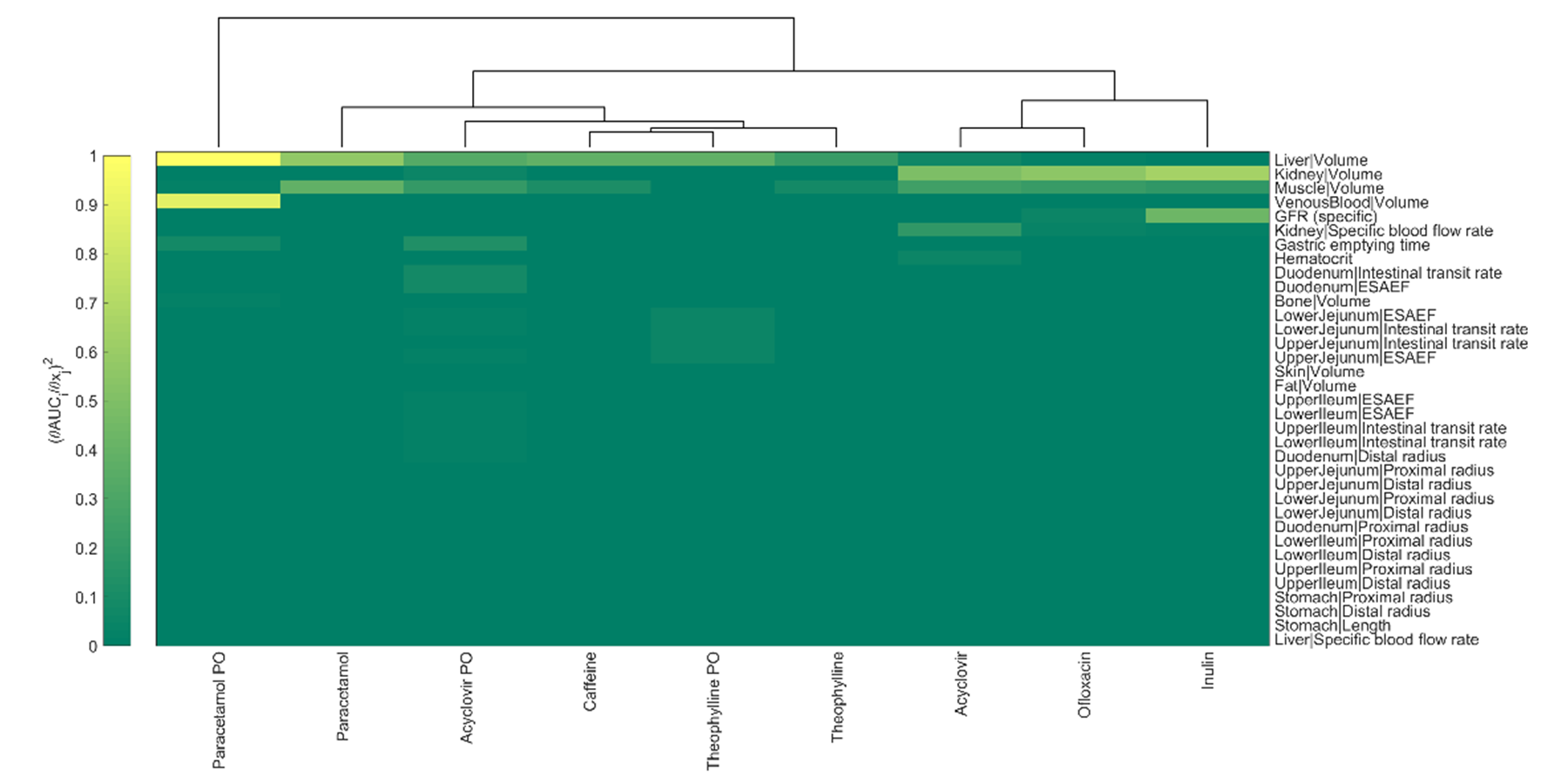

Supplement: S11 Fig — The sensitivity index is defined in Eq 1 of the Materials and Methods section in the main text. ESAEF stands for Effective Surface Area Enhancement Factor. (TIF) [file pone.0194294.s011.tif]

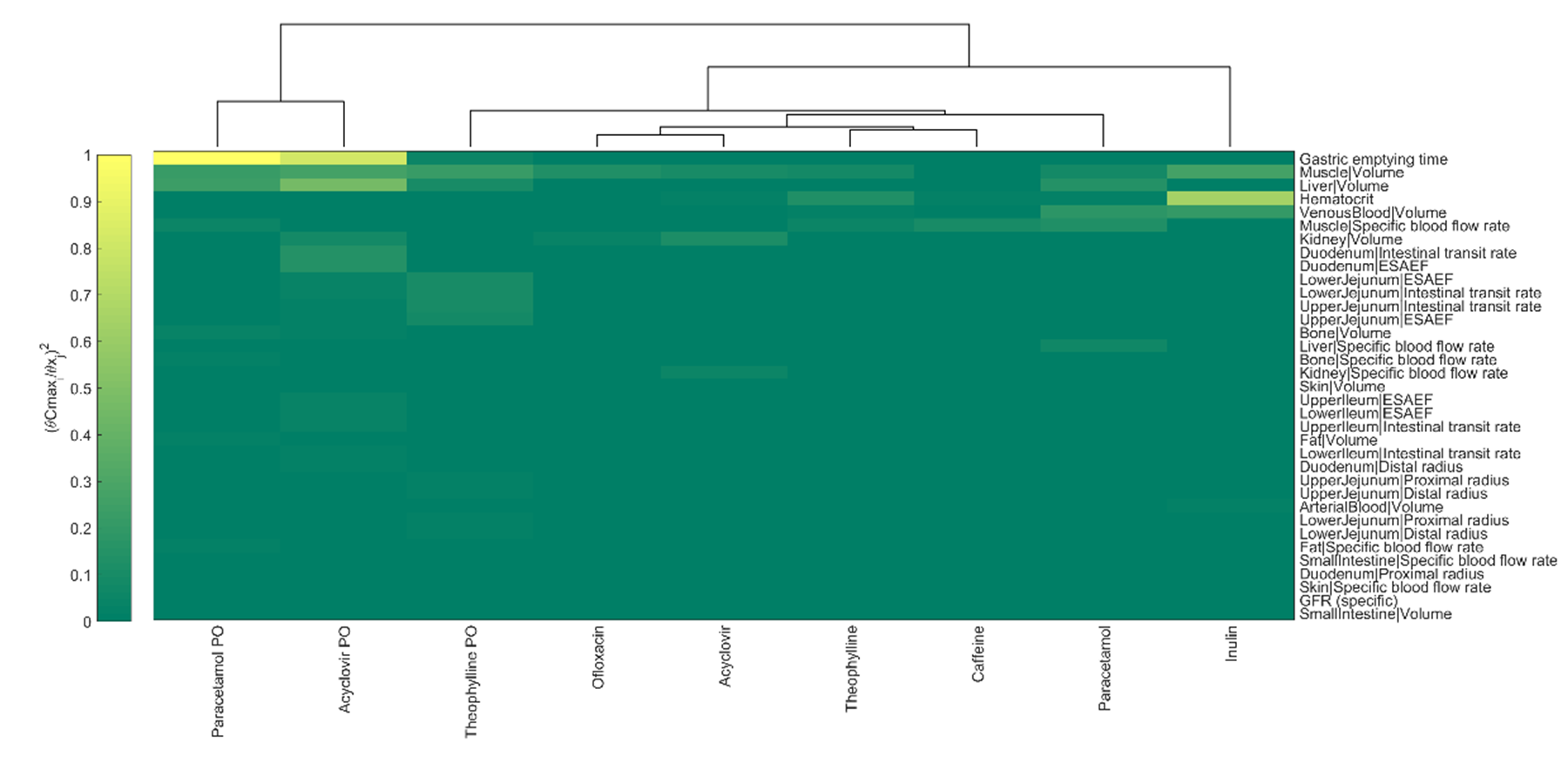

Supplement: S12 Fig — The sensitivity index is defined in Eq 1 of the Materials and Methods section in the main text. ESAEF stands for Effective Surface Area Enhancement Factor. (TIF) [file pone.0194294.s012.tif]
