# Supplementary material for: Development and validation of a physiology-based model for the prediction of pharmacokinetics/toxicokinetics in rabbits
Source: PLoS One. 2018 Mar 21;13(3):e0194294. doi: 10.1371/journal.pone.0194294 (PMC5862475; doi:10.1371/journal.pone.0194294)
Supplement: S1 Appendix — Rabbit PBPK model validation with Inulin, Caffeine, Ofloxacin, Theophylline, Paracetamol, and Acyclovir. (DOCX) [file pone.0194294.s013.docx]

Supporting Information

# Rabbit physiology (parameterization)

## Organ volumes

The rabbit organ volumes were extracted from the literature and compared in Table A. For the weight-volume conversion, a density of 1 g/cm^3^ was used. This assumption is adequate for almost all organs. All parameters selected and applied in the rabbit model are in bold and underlined in Table A.

**Table A:** **Organ volumes in litres: comparison of the values found for rabbits in the literature.** The values used in the rabbit model are in bold and underlined (* testes).

| Literature / Organ | Davies, B. & Morris, T., 1993[1] | Sweeney, L. M. et al*.*, 2009[2] | Skelton, H., 1927[3] | Crile, G. & Quiring, D. P., 1940[4] | Sweeney, L. M. et al*.*, 2009[2] | Lebas, F., 1997 [5] | Debray, L. et al., 2003[6] | Brown, W. H. et al., 1925 [7] | Scaled by BW |
| --- | --- | --- | --- | --- | --- | --- | --- | --- | --- |
| Arterial Blood | **0.050** | 0.0370 | 0.051 |  |  |  |  |  |  |
| Bone |  |  | **0.310** |  |  |  |  |  |  |
| Brain | **0.014** | 0.005 | 0.015 | 0.0101 |  |  |  | 0.0105 |  |
| Fat | **0.120** | 0.200 |  |  |  |  |  |  |  |
| Gonads* |  |  |  |  | 0.0045 |  |  | **0.005** |  |
| Heart | **0.006** |  | 0.022 | 0.009 |  |  |  | 0.006 |  |
| Kidney | **0.015** | 0.018 | 0.018 | 0.018 |  |  |  | 0.014 |  |
| Large Intestine (LI) |  |  |  |  |  | **0.030** |  |  |  |
| Liver | **0.100** | 0.088 | 0.130 | 0.083 |  |  |  | 0.096 |  |
| Lung | **0.017** | 0.031 |  | 0.014 |  |  |  |  |  |
| Muscle | **1.350** |  | 1.305 |  |  |  |  |  |  |
| Pancreas |  |  |  |  |  |  | **0.0036** |  |  |
| *Portal Vein* |  |  |  |  |  |  |  |  | **0.027** |
| *Skin* | **~~0.110~~ 0.2534^§^** | 0.425 | 0.330 |  |  |  |  |  |  |
| Small Intestine (SI) |  |  |  |  |  | **0.06** |  |  |  |
| Spleen | **0.001** |  | 0.0025 |  |  |  |  | 0.0011 |  |
| Stomach |  | 0.025 |  |  |  | **0.020** |  |  |  |
| Venous Blood | **0.1150** | 0.1106 | 0.1176 |  |  |  |  |  |  |

**^§^** The missing weights in the model (weight=sum of used organ volumes and blood pools) are compensated for by increasing the skin volume from 0.110 to 0.2534 kg, which is still within the reported range (Table A).

The total volumes of the organs and the blood pools are summed to the total weight of the rabbit by assuming a density of 1 g/cm^3^. The reported organ volumes are linearly scaled to a unified rabbit body weight of 2.5 kg [1].

The Portal Vein volume could not be found in the literature and was estimated as follows: The mean ratio of portal vein volume of beagle, cat, dog, mouse, rat, and minipig to BW is r=0.011 (S.D. +/-0.0033). This ratio was applied to the bodyweight of the rabbit to estimate a portal vein volume.

## Haematocrit-Glomerular Filtration Rate (GFR) specific

The rabbit haematocrit and Glomerular Filtration Rate (GFR) specific values are listed in Table B.

Table B: Haematocrit and GFR (specific) of adult New Zealand white rabbits.

| Parameter | Value | Source |
| --- | --- | --- |
| Haematocrit (%) | 41 | Houdebine, L.-M., 2009[8] |
| GFR (specific) (l/min/kg kidney) | 0.6 | Davies, B. & Morris, 1993[9] |

## Specific blood flow rates

Table C summarizes the specific blood flow rates for rabbits extracted from the literature. Similar to the organ volumes (Table A), the literature source that contained information for more tissues was preferably used [9]. When tissue data were not available, additional values were chosen from the literature sources that contained data as complete as possible.

Table C: Specific blood flow rates (l/min/kg organ) in rabbits. The values used in the presented rabbit model are in bold and underlined. *Our modelling approach does not require a specific blood flow rate for the lungs; the blood flow rate to the lungs is calculated as the sum of all other flow rates.

| Organ | Davies, B. & Morris, 1993[1] | Davis, T. et al., 1990[10] | Bill, A., 1979[11] | Cianci, T. et al., 1990[12] | Lifson, N. et al., 1980[13] | Sweeney, L. M. et al*., 2009*[14] | Sweeney, L. M. et al*.*, 2009[2] | Ünlüer, S. et al., 1984[15] |
| --- | --- | --- | --- | --- | --- | --- | --- | --- |
| Bone |  | **0.15** |  |  |  |  |  |  |
| Brain |  |  | 1.24 |  |  | **0.678** |  |  |
| Fat | **0.267** |  |  |  |  | 0.137 |  |  |
| Gonads |  |  |  |  |  |  |  | **0.358** |
| Heart | **3.2** |  | 0.0582 |  |  |  |  |  |
| Kidney | **6.154** |  | 6.35 |  |  | 2.326 |  |  |
| Large intestine (LI) |  |  |  | **0.47** |  |  |  |  |
| Liver | **2.299** |  | 0.13 | 0.1 |  | 0.035 |  |  |
| Muscle | **0.115** |  |  |  |  |  |  |  |
| Lung* |  |  |  |  |  |  |  |  |
| Pancreas |  |  |  |  | **1** |  |  |  |
| Skin |  |  |  |  |  | 0.072 | **0.158** |  |
| Small intestine (SI) |  |  | 1.26 | **0.92** | 5.85 |  |  |  |
| *Spleen* | **9** |  | *3.88* | 5.85 |  |  |  |  |
| Stomach |  |  | 1.64 | **1.05** |  | 0.705 |  |  |

## Gastrointestinal tract (GIT)-related parameters

Rabbits are considered to have a monogastric digestive system. However, the rabbit has a much larger caecum than humans and most other monogastric species, and the proximal colon exhibits different behaviour depending on the time of day [5]. In the rabbit model developed in this work, changes were made to the GIT-specific parameters when the respective literature was available. The basic structure of the model remained the same as the species already included in the PK-Sim^®^ (i.e., human, monkey, beagle, dog, minipig, rat, and mouse).

### GIT-pH

The pH values from the literature for the GIT sections are listed in Table D. Similar to the organ volumes and the specific blood flow rates, the literature source that contains the most complete data set was used [16].

Table D: Literature based rabbit pH values for PK-Sim^®^ gastrointestinal sections. The values used in the rabbit model are in bold and underlined.

| Organ | Davies, B. & Morris, 1993[1] | Lebas, F. et al., 1997[5] | Kararli, T., 1995 [16] | K Rozman, K., 1988 [16, 17] |
| --- | --- | --- | --- | --- |
| Stomach | 1.9 | 1.5 to 2.0 | **1.9** | 1.9 |
| Duodenum | 6.0 | 7.2 | **6.0** |  |
| Lower Jejunum |  | 7.2 | **6.8** | 7.5 |
| Upper Jejunum |  | 7.2 | **6.8** | 7.5 |
| Lower Ileum | 8.0 | 7.2 | **7.5** |  |
| Upper Ileum | 8.0 | 7.2 | **8.0** |  |
| Caecum | 6.6 | 6.0 | **6.6** | 6.6 |
| Colon | 7.2 | 6.5 | **7.2** | 7.2 |
| Rectum | 7.2 |  | **7.2** | 7.2 |

### GIT-dimensions/intestinal transit rates

The bibliographic data available for the GIT dimensions are shown in Table E. The literature source that contained data for the most tissues [5] was used for the rabbit PBPK model. The data from the other sources were used to supplement the absent measurements. Some dimensions could not be found in the literature, and best guesses were used. For example, as the generic PBPK model structure contains 5 small intestine (SI) compartments, the total length of the small intestine (SI) was divided by the number of compartments to give a length per compartment of 30/5=6 dm (values not shown in Table E). The radius (i.e., distal and proximal) was assumed to be constant at 0.05 dm throughout. The length of the rectum was estimated from [18] to be 0.1 dm; the radius was assumed to be the same as that of the colon.

Table E: Literature values for the rabbit GI section dimensions in decimetres (dm). The values used in the rabbit model are in bold and underlined. The compartments of the small intestine are the duodenum, upper/lower ileum, and upper/lower jejunum. The compartments of the large intestine are the caecum, colon ascendens/descendens/transversum, and rectum.

| Organ | Parameters (dm) | Lebas, F. et al., 1997[5] | Schulze-Delrieu, K. & Wall, J. P., 1983[19] | Kararli, T., 1995[16] | Hatton, G. B. et al., 2015[18] | de Zwart, L., 1999[20] | Sibly, R. et al., 1990[21] | Rees Davies, R. et al., 2003[22] | DOROTEA, S. B. et al., 2016[23] |
| --- | --- | --- | --- | --- | --- | --- | --- | --- | --- |
| Stomach | Proximal radius |  | **0.15** |  |  |  |  |  |  |
|  | Distal radius |  | **0.15** |  |  |  |  |  |  |
|  | Length |  | **1.4** |  |  |  |  |  |  |
| Duodenum | Proximal radius | **0.04** |  |  |  |  |  |  |  |
|  | Distal radius | **0.05** |  |  |  |  |  |  |  |
|  | Length |  |  |  |  |  |  |  |  |
| Lower Ileum | Proximal radius |  |  |  |  |  |  |  |  |
|  | Distal radius |  |  |  |  |  |  |  |  |
|  | Length |  |  |  |  |  |  |  |  |
| Lower Jejunum | Proximal radius |  |  |  |  |  |  |  |  |
|  | Distal radius |  |  |  |  |  |  |  |  |
|  | Length |  |  |  |  |  |  |  |  |
| Upper Ileum | Proximal radius |  |  |  |  |  |  |  |  |
|  | Distal radius |  |  |  |  |  |  |  |  |
|  | Length |  |  |  |  |  |  |  |  |
| Upper Jejunum | Proximal radius |  |  |  |  |  |  |  |  |
|  | Distal radius |  |  |  |  |  |  |  |  |
|  | Length |  |  |  |  |  |  |  |  |
| Small Intestine | Total Length | **30** |  | 35.6 | 15.1 | 15.1 | 23-46 |  |  |
|  | Average radius |  |  |  |  |  | ≈0.03^$^ |  |  |
| Caecum | Proximal radius | **0.15** |  |  |  |  |  |  |  |
|  | Distal radius | **0.2** |  |  |  |  | ≈0.1^$^ |  |  |
|  | Length | **4.5** |  | 6.1 | 4.4 | 4.4-6.1 | 2.4-4.1 | 4 |  |
| Colon Ascendens | Proximal radius |  |  |  |  |  |  |  |  |
|  | Distal radius |  |  |  |  |  |  |  |  |
|  | Length |  |  |  |  |  |  | **1** |  |
| Colon Transversum | Proximal radius |  |  |  |  |  |  |  |  |
|  | Distal radius |  |  |  |  |  |  |  |  |
|  | Length |  |  |  |  |  |  | **2** |  |
| Colon Descendens | Proximal radius |  |  |  |  |  |  |  |  |
|  | Distal radius |  |  |  |  |  |  |  | **0.05** |
|  | Length |  |  |  |  |  |  | **0.4** |  |
| Colon Sigmoid | Proximal radius |  |  |  |  |  |  |  |  |
|  | Distal radius |  |  |  |  |  |  |  |  |
|  | Length |  |  |  |  |  |  | **10** |  |
| Rectum | Proximal radius |  |  |  |  |  |  |  |  |
|  | Distal radius |  |  |  |  |  |  |  |  |
|  | Length |  |  |  | **0.1** |  |  |  |  |
| Colon Total | Length | 15 (5 “proximal”*) |  | 16.5 | 12.3 | 12.3-16.5 | 1.9-4.3 |  |  |
| GI tract | Total Length | 45 to 50 |  |  |  |  |  |  |  |

For a certain GIT compartment in PK-Sim^®^, the intestinal transit rate (ITT) is generally defined as the inverse of the fractional length of the compartment. For the rabbit, using the current GIT parameterization for an adult rabbit of 2.5 kg, we calculated and used the values given in Table F.

Table F: Literature values for the rabbit GI section dimensions in decimetres (dm) and their forward calculations to intestinal transit rates (ITT). The compartments of the small intestine (SI) are the duodenum, upper/lower ileum, and upper/lower jejunum (marked with an asterisk). The compartments of the large intestine (LI) are the caecum, colon ascendens/descendens/sigmoid/transversum, and rectum (marked with a dollar symbol).

| Compartment | Length of compartment (Table E)  (dm) | Length of compartment/Length of SI or LI as appropriate | 1/ fractional length  i.e., ITT value |
| --- | --- | --- | --- |
| Duodenum* | 6 | 0.2 | 5 |
| UpperJejunum* | 6 | 0.2 | 5 |
| LowerJejunum* | 6 | 0.2 | 5 |
| UpperIleum* | 6 | 0.2 | 5 |
| LowerIleum* | 6 | 0.2 | 5 |
| Caecum^$^ | 4.5 | 0.241 | 4.148 |
| ColonAscendens^$^ | 1 | 0.054 | 18.667 |
| ColonTransversum^$^ | 2 | 0.143 | 7 |
| ColonDescendens^$^ | 0.4 | 0.021 | 46.667 |
| ColonSigmoid^$^ | 10 | 0.536 | 1.867 |
| Rectum^$^ | 0.1 | 0.005 | 186.667 |

### GIT-transit/emptying times

Table G shows the literature values for the GIT emptying and transit times.

Table G: Literature values for rabbit GI times. In PK-Sim®, the small and large intestinal transit times are defined as the times when 63% of the compound has passed through the small and large intestines, respectively.

| PK-Sim^®^ parameter | Lebas, F. et al., 1997[5] | Sohn, J. & Couto, M. A., 2012[24] | Hatton, G. B., 2015[18] | de Zwart, L., 1999 [20] | Rees Davies, R. & Rees Davies, J. A., 2003  [22] |
| --- | --- | --- | --- | --- | --- |
| Gastric emptying time (GET) | 3 to 6 hrs | 3-6 hrs | 30 mins, fasted, solution; 3 – 6 hrs |  | 3-6 hrs; cecotrophs 6-8 hrs |
| Small intestinal transit time (SITT) | 1.5 hrs | 10 to 20 mins (jejunum) plus 30 to 60 mins (ileum) | 10 to 20 mins (jejunum) plus 30 to 60 mins (ileum) |  | 10 to 20 mins (jejunum) plus 30 to 60 mins (ileum) |
| Large intestinal transit time (LITT) | =20-(5+1.5)  =13.5 hrs |  |  | 3.8 hrs |  |

In PK-Sim^®^, the small and large intestinal transit times (SITT/LITT) are indirectly inferred by calculating the time until the 90% of the compound passes through the small or the large intestine respectively. The transit times (Table G) are transformed to transit rates by an optimized factor (Table H) in the model. This transformation allows the transit times to be changed to compound specific values.

Table H: The optimized values of the small/large intestinal transit time factor slope. The 95% confidence intervals are shown in parentheses.

| Parameter Name | Value |
| --- | --- |
| Small Intestinal Transit Time Factor Slope (1/min) | 0.29 (+/- 0.16) |
| Large Intestinal Transit Time Factor Slope(1/min) | 0.58 (+/- 0.03) |

### GIT-Effective Surface Areas enhancement factor

The absorption of compounds is mainly driven by the surface of the intestine. The surface area is increased substantially by circular folds and villi [25, 26]. In the PBPK model, the GIT segments are approximated by a tube, and the larger surface of the intestine is accounted for by the effective surface area enhancement factor (ESAEF) parameters. The available literature for a rabbit’s ESAEF is shown in Table I.

Table I: Rabbit effective surface area enhancement factors *Rabbit weight 3418.33 ± 640.12 g.

| References | Duodenum | UpperJejunum | lowerJejunum | UpperIleum | LowerIleum | Caecum | ColonAscendens | ColonTransversum | ColonDescendens | ColonSigmoid | Rectum |
| --- | --- | --- | --- | --- | --- | --- | --- | --- | --- | --- | --- |
| Snipes, R. L., 1997[25] | SI: 3.5 | | | | | 2.18 | Colon: 1.80 | | | | |
| Snipes, R. L., 1997[25]* | SI: 2.8366 ± 0.6244 | | | | | 2.0266 ± 0.4248 | 1.9470 ± 0.2385 | | | | |
| Thomson, A. et al., 1987 [27] |  | 12.3 ± 0.8 | 12.3 ± 0.8 | 25.4 ± 1.8 | 25.4 ± 1.8 |  |  |  |  |  |  |
| Values from Thomson, A. et al., 1987 [27] multiplied by 15 to include effect of microvilli |  | 184.5 | 184.5 | 381 | 381 |  |  |  |  |  |  |
| Westergaard, H. & Dietschy, J. M., 1974[28]$ |  | 592 (24.7) | 592 (24.7) |  |  |  |  |  |  |  |  |

The effective surface area enhancement factors of the small intestinal (SI) luminal compartments used in the rabbit model were optimized based on i) the available literature boundaries (Table I) and ii) the PK of the compounds administered orally. As described below, for the rabbit, data from three compounds p.o. were considered: acyclovir, paracetamol, and theophylline. To fit the available p.o. data for the three compounds, the effective surface area enhancement factors were globally optimized among all compounds (Table J), and the administration related parameters were changed individually (Results/Materials and Methods sections).

Table J: The optimized values of a rabbit’s effective surface area enhancement factor as well as additional parameters. The 95% confidence intervals are shown inside the parentheses, i.e., except for the lower and upper jejunum the parameters were not sensitive.

| Parameter Name | Acyclovir/Paracetamol/Theophylline PO |
| --- | --- |
| Effective Surface Area Enhancement Factor\|Duodenum | 584. 97 (+/- 2603.93) |
| Effective Surface Area Enhancement Factor\|UpperJejunum | 568.64 (+/- 451.78) |
| Effective Surface Area Enhancement Factor\|LowerJejunum | 592 (+/- 397.51) |
| Effective Surface Area Enhancement Factor\|UpperIleum | 584.36 (+/- 1342.73) |
| Effective Surface Area Enhancement Factor\|LowerIleum | 581.36 (+/- 1445,03) |

## Tested compounds and their physicochemical properties

Table K lists the tested compounds along with pathways involved in their clearance.

Table K: Compound selection used to validate the rabbit model. GFR=glomerular filtration rate, TS=tubular secretion. An X signifies the existence of rabbit data, based on which model was validated; i.v. stands for intravenous administration and p.o. for oral.

| Compounds | Inulin | Caffeine | Ofloxacin | Paracetamol | Theophylline | Acyclovir |
| --- | --- | --- | --- | --- | --- | --- |
| *Clearance Processes* | GFR | Hepatic | GFR/TS, Hepatic | GFR, Hepatic | TS, Hepatic | GFR/TS,  Hepatic |
| i.v. | **X** | **X** | **X** | **X** | **X** | **X** |
| p.o. |  |  |  | **X** | **X** | **X** |

Table L shows the physicochemical properties of the compounds chosen. The information was gained both from in-house data as well as publicly available sources (i.e., DrugBank)

Table L: Physicochemical properties of the compounds chosen to validate the rabbit model. Source: DB (DrugBank), CS (Chemspider), or IHD (in house data).

| Compound | LogP | Solubility (mg/L) | Fu (%) | MW | pKa |
| --- | --- | --- | --- | --- | --- |
| *Inulin* | -10 (IHD) | 280 (DB) | 100(DB) | 6179.36(DB) | 11.27 (DB) |
| *Caffeine* | 0.07 (DB) | 21600 (DB) | 70 (DB) | 194.2 (CS) | 0.8 |
| *Ofloxacin* | 0 (IHD) | 28300 (DB) | 68 (DB) | 361.37 (DB) | - |
| *Paracetamol* | 1.25 (IHD) | 17314 (CS) | 82 (IHD) | 151.16 (DB) | 9.41(DB) |
| *Theophylline* | -0.02 (DB) | 7360 (DB) | 60 (DB) | 180.17 (DB) | 7.82 (CS) |
| *Acyclovir* | 0.1 (IHD) | 33990 | 82 (DB) | 225.21 (DB) | 9.2 (DB) |

## Pharmacokinetic profiles/Model predictions

The details of the model development and scaling can be found in the main text. An overview for all active processes’ parameters are shown in Table M.

### Inulin

S1 Fig shows the simulation and observation of venous blood plasma concentration of inulin, based on the experiment of [29]. New Zealand white rabbits with a mean weight of 2.85 kg received two intravenous doses of inulin, 40 mg/kg (S1 Fig, blue dots) and 60 mg/kg (S1 Fig, green dots).

The rabbit inulin PBPK model predicts a PK close to the observations of both doses (blue and green solid lines). Later time points (approximately 2 hr) may hint towards an overprediction of the clearance, which could be captured by a lower GFR_specific_. Overall, the model prediction is acceptable without any parameter changes.

S1 Fig: Simulation of two intravenous administrations of inulin (40 mg/kg blue, 60 mg/kg green) in rabbits weighing 2.85 kg. The solid lines show the simulated venous blood concentration profile and the dots represent the experimental data [29]. No parameter optimization was performed in order to capture inulin’s pharmacokinetic profiles in both doses.

### Caffeine

Caffeine plasma clearance is dominated by metabolism that primarily occurs in the liver [30]. As such, an overall liver clearance was hypothesized, and the kinetic parameters were adopted from our previous work [31]. S2 Fig shows the simulation and observation of 3.5 kg New Zealand white rabbits that were administered 4 mg/kg caffeine intravenously [32]. The dotted line is the rabbit PBPK simulation when the parameters remained unchanged from our previous work [31]. The solid lines show the simulated profiles as fraction unbound (f_u_) and K_m_, which were adjusted to 0.8 (from 0.7) and 300 (from 400) μmol/L to better fit the experimental data. The extent of the parameter calibration was small and within the range reported in the literature. The rabbit PBPK model successfully describes the available data.

S2 Fig: Simulations of venous blood plasma for 4 mg/kg intravenous administration of caffeine in rabbits weighing 3.5 kg. The dotted line shows the simulated profile when no parameters were changed. The solid line shows the simulated profile when the fraction unbound (f_u_) and K_m_ were adjusted to 0.8 (from 0.7) and 300 (from 400) μmol/L respectively, and the green dots are data adopted from the work of [32].

### Ofloxacin

Another compound used for the validation of the rabbit PBPK model is ofloxacin. The compound is cleared by GFR and tubular secretion and undergoes a limited degree of metabolism [33]. Similar to caffeine, metabolism has been hypothesized to be localized in the liver. S3 Fig represents the experiment of [34] where New Zealand white rabbits weighing 2.5 kg was administered 20 and 40 mg/kg of ofloxacin intravenously. The solid lines present the rabbit PBPK simulations for the two different doses (20 mg/kg, blue, and 40 mg/kg, green) in venous blood, along with the corresponding experimental data. The dark grey line shows the fraction excreted in the urine for the 20 mg/kg dose. To describe the data, tubular secretion and hepatic clearance were re-estimated while the GFR_fraction_ was kept at 1 to further denote that 100% of the filtrated compound was cleared. The ratio of the hepatic clearance to tubular secretion was calibrated by considering reports in the literature of the urine excretion of ofloxacin (70-90%) [35] (S3 Fig).

S3 Fig: Simulations of 20 and 40 mg/kg i.v. administration of ofloxacin in rabbits: Venous blood plasma vs. time. The solid lines are the simulations of 20 mg/kg (green) and 40 mg/kg (blue). The dark grey line depicts the fraction excreted in the urine for the small dose. The dots are observed data [34]. Hepatic clearance and tubular secretion were calibrated to 0.5 and 0.1 L/min respectively in order to capture the experimentally observed fraction of ofloxacin excreted in the urine (grey line, 70-90%). The concentration vs time data were then simulated (blue, green lines).

### Theophylline

Based on our previous work with theophylline and data from the literature [36], the clearance pathways were known to involve tubular secretion as well as metabolic clearance. For theophylline, there were two experimental data sets available on which the PBPK model was tested. First, in the work of [37], 12 mg/kg of theophylline was administered intravenously in New Zealand white rabbits weighing 2.5 kg. S4 Fig shows the simulated plasma profile from the experiment of [37] with slight calibrations of the fraction unbound (0.8 instead of 0.5) and the liver clearance (0.07 instead of 0.04 1/min).

S4 Fig: Simulation of venous blood plasma for 12 mg/kg intravenous administration of theophylline in rabbits weighing 2.5 kg. The green dots are the observed data [37]. In order to capture the PK data, fraction unbound was calibrated from 0.8 to 0.5, and liver clearance from 0.07 to 0.04 l/min.

The second experimental data set was from the work of [38], where 15 mg/kg of theophylline was administered intravenously in New Zealand white rabbits weighing 3.15 kg. S5 Fig shows the simulated profiles for four different rabbits. The observed data indicate differences in the clearance in the individuals. This difference was captured by varying the hepatic clearance in the model, while the tubular secretion rate was kept constant to the value observed in the literature [36]. To capture the observed data, the liver clearance ranged from 0.05 to 0.15 1/min.

S5 Fig: Simulation of venous blood plasma for 15 mg/kg intravenous administration of theophylline in New Zealand white rabbits weighing 3.15 kg and with increasing hepatic clearances from green to grey. The dots are the observed data from the work of [38] for four individual rabbits. In order to capture the experimental data liver clearance ranged from 0.05 to 0.15 l/min.

In the same work of [38], an oral administration of 200 mg was given to the rabbits, and S6 Fig shows the simulation as well as the experimental data. The model was able to explain the observed data by identifying the gastric emptying time (GET=1.66 hr), and dissolution time (10 min) within the range of the literature data (Table F).

S6 Fig: Simulation of venous blood plasma for 200 mg oral administration of theophylline in New Zealand white rabbits weighing 3.15 kg. The dots represent the observed data from the work of [38]. To describe the experimental data the gastric emptying time (GET) was set to 1.66 hr and dissolution time to 10 min which are values inside the ranges found in literature (Table G).

### Paracetamol

Paracetamol is a compound included in the PK-Sim^®^ database that includes physicochemical properties and clearance processes (i.e., GFR, metabolism). Similar to the case of theophylline, our model was able to simulate the plasma concentration profile of two different experiments of paracetamol administration [39, 40]. S7 Fig shows the observations of 35 mg/kg paracetamol i.v. administration to a 3.1 kg rabbit [39], as well as the respective simulated PK profile. To describe the data, the kinetics of the active metabolic process in the liver (Vmax, K_m_) were adjusted.

S7 Fig: Simulation of the venous blood plasma for 35 mg/kg intravenous administration of paracetamol in rabbits weighing 3.17 kg. The green dots are the data adopted from the work of [39]. CYP protein mediated hepatic clearance was parameterised with Vmax = 40 µmol/L/min, Km = 10 µmol/L and considering a liver concentration of the enzyme of 1µmol/L.

Without any changes to the parameters calculated from the previous experiment of [39], the rabbit PBPK model was able to predict the PK-data from the experiment of [40], where 35 mg/kg was administered in New Zealand white rabbits weighing 3.61 kg. S8 Fig shows the simulated plasma profile along with the observed data [40].

S8 Fig: Simulation of venous blood plasma for 35 mg/kg intravenous administration of paracetamol in rabbits weighing 3.61 kg. Green dots: observed data [40]. Similar to previous experiment shown in S7 Fig, CYP protein mediated hepatic clearance was parameterised with Vmax = 40 µmol/L/min, Km = 10 µmol/L and considering a liver concentration of the enzyme of 1µmol/L

### Acyclovir

Acyclovir is included in the PK-Sim^®^ database, and the information adopted from other species was applied in the rabbit model. Processes include GFR, tubular secretion, and liver clearance [41]. S9 Fig shows the simulated and observed [42] plasma profiles of acyclovir as 60 mg/kg acyclovir administration to 3.6 kg New Zealand white rabbits. Similar to ofloxacin, the relative weights of the renal and hepatic clearances were adjusted to describe the fraction excreted in the urine of 70%, as reported in [43].

S9 Fig: PK-Sim® simulation (blue line) and the observed data [42] (green dots) of venous blood plasma profiles of acyclovir, 60 mg/kg i.v. The grey line is the simulated fraction excreted in urine. The relative weights of the renal and hepatic clearances were adjusted to 0.3 L/min to describe the fraction excreted in the urine of 70%. The concentration vs time profile (blue line) was then simulated.

In the same work [42], 300 mg/kg oral administration of acyclovir in 3.6 kg rabbits is described. S10 Fig shows the observed data and the simulated plasma profiles. To describe the data, the gastric emptying time was increased to 4.91 hr, which is inside the bounds observed in the literature (Table F).

S10 Fig: PK-Sim® simulation of venous blood plasma profiles for 300 mg/kg oral administration of acyclovir in rabbits weighing 3.6 kg. The green dots are data adopted from the work of [42]. To describe the data, the gastric emptying time was increased to 4.91 hr from 0.5 hr, which is inside the bounds observed in the literature (Table F).

**Table M: Overview of active processes’ parameters for the simulation of PK data of inulin, caffeine, ofloxacin, theophylline, paracetamol, and acyclovir (S1-S10 Figs).** References near the values indicate the source of PK data as basis of parameter identification. “--“ indicates no relevance of the particular process for a compound (e.g. no active processes for Inulin), or use of a default value (e.g. Vmax = 1 µmol/L).

| Compounds | f_u_ | V_max_  (μmol/L) | K_m_ (μmol/L) | Hepatic clearance (L/min) | Renal clearance (L/min) | Tubular secretion  (L/min) | Gastric Emptying Time (GET) (hr) | Dissolution time (min) |
| --- | --- | --- | --- | --- | --- | --- | --- | --- |
| Inulin | -- | -- | -- | -- | -- | -- | -- | -- |
| Caffeine | 0.8 [32] | -- | 300 [32] | -- | -- | -- | -- | -- |
| Ofloxacin | -- | -- | -- | 0.5 [34] | -- | 0.1 [34] | -- | -- |
| Theophylline | 0.5 [37] | -- | -- | 0.04 [37]  0.05-0.15 [38] | -- | -- | 1.66 [38] | 10 [38] |
| Paracetamol | -- | 40 [39] | 10 [39] | -- | -- | -- | -- | -- |
| Acyclovir | -- | -- | -- | 0.3 [42] | 0.3 [42] | -- | -- | -- |

### Summary table

Table N shows a summary of the observed versus predicted and optimized Cmax values for the experiments. The prediction error is based on the base rabbit model plus compound-specific information (e.g., clearance pathways, solubility, logP, etc.) derived in vitro or from other species. Furthermore, those models were optimized to adopt the biological processes of the rabbit species. The physiological parameters that were fitted in the optimization are substance specific and are reported in the previous sections of the Supplementary material.

Table N: Review table comparing the observed Cmax with the predicted Cmax and with values from the optimized models.

| Compounds | Cmax observed (μmol/L) | | Cmax Predicted (μmol/L) | | Cmax Optimized (μmol/L) | | % Cmax Error (Predicted) | | | % Cmax Error (Optimized) | | Tissue/Administration | | Literature | |
| --- | --- | --- | --- | --- | --- | --- | --- | --- | --- | --- | --- | --- | --- | --- | --- |
| Inulin | | 16.31 | | 17.09 | | - | | 4.56 | - | | Venous Blood  (40 mg/kg)/i.v. | | Michigoshi, Y. et al., 2011 [29] | |  |
|  | | 27.16 | | 25.63 | | - | | 5.97 | - | | Venous Blood  (60 mg/kg) /i.v. | | Michigoshi, Y. et al., 2011 [29] | |  |
|  | | 532.56 | | 381.47 | | 367.59 | | 39.61 | 44.88 | | Plasma/i.v. | | Tsuji, A. et al., 1985 [44] | |  |
|  | | 25.30 | | 85.30 | | 28.45 | | 70.34 | 11.07 | | Lung/i.v. | | Tsuji, A. et al., 1985 [44] | |  |
|  | | 19.89 | | 9.36 | | 30.19 | | 112.50 | 34.12 | | Skin/i.v. | | Tsuji, A. et al., 1985 [44] | |  |
|  | | 11.82 | | 18.08 | | 16.11 | | 34.62 | 26.63 | | Bone/i.v. | | Tsuji, A. et al., 1985 [44] | |  |
|  | | 23.16 | | 26.23 | | 21.91 | | 11.70 | 5.71 | | Heart/i.v. | | Tsuji, A. et al., 1985 [44] | |  |
|  | | 7.60 | | 12.74 | | 10.79 | | 40.35 | 29.56 | | Muscle/i.v. | | Tsuji, A. et al., 1985 [44] | |  |
|  | |  | |  | |  | |  |  | |  | |  | |  |
| Caffeine | | 36.06 | | 50.28 | | 45.82 | | 28.29 | 21.31 | | Venous Blood/i.v. | | Beach, C. A. et al., 1985 [32] | |  |
|  | |  | |  | |  | |  |  | |  | |  | |  |
| Theophylline | | 97.22 | | 193.66 | | 143.70 | | 49.80 | 32.35 | | Venous Blood/i.v. | | Celardo, A., 1985 [37] | |  |
|  | | 272.68 | | 267.59 | | 273.75 | | 1.90 | 0.39 | | Venous Blood/i.v. | | El-Yazigi, A. & Sawchuk, R. J., 1981 [38] | |  |
|  | |  | |  | |  | |  |  | |  | |  | |  |
| Theophylline (PO) | | 248.04 | | 126.17 | | 253.68 | | 96.59 | 2.22 | | Venous Blood/Typical Tablet | | El-Yazigi, A. & Sawchuk, R. J., 1981 [38] | |  |
|  | |  | |  | |  | |  |  | |  | |  | |  |
| Paracetamol (PO) | | 47.46 | | 26.32 | | 47.86 | | 80.32 | 0.84 | | Venous Blood/Solution | | Ishikawa, T. et al., 2001 [45] | |  |
|  | | 31.64 | | 26.30 | | 30.40 | | 20.30 | 4.08 | | Venous Blood/Rapid Tablet | | Ishikawa, T. et al., 2001 [45] | |  |
|  | | 17.23 | | 0.47 | | 13.41 | | 3565.96 | 28.49 | | Venous Blood/Typical Tablet | | Ishikawa, T. et al., 2001 [45] | |  |
|  | |  | |  | |  | |  |  | |  | |  | |  |
| Acyclovir (PO) | | 21.55 | | 90.66 | | 23.64 | | 76.23 | 8.84 | | Venous Blood/Solution | | van Jaarsveld, M. F. et al., 2007 [42] | |  |

## Sensitivity analysis

In S11 and S12 Figs, the squared sensitivity index, as defined in Equation 1 of the Materials and Methods section, is shown for the 35 most sensitive physiological parameters for AUC and Cmax changes, respectively. The other, less sensitive, parameters are not shown.

S11 Fig: Sensitivity analysis for i.v. and p.o. (PO) administrations regarding the change in AUC. The sensitivity index is defined in Equation 1 of the Materials and Methods section in the main text. ESAEF stands for Effective Surface Area Enhancement Factor.

S12 Fig: Sensitivity analysis for i.v. and p.o. (PO) administrations regarding the change in Cmax. The sensitivity index is defined in Equation 1 of the Materials and Methods section in the main text. ESAEF stands for Effective Surface Area Enhancement Factor.

## Complete list of rabbit PBPK model parameters

Table O: List of the Rabbit PBPK model parameters (n=977). The type classification indicates if the parameter value is specific for the rabbit (n=61), specific for all species (n=60), transferred from the mouse PBPK model (n=53), shared between all species (of a selection of 7 pre-clinical species, n=713), shared between several species (>3 species, n=90).

| Parameter Name | Value in Rabbit PBPK Model | Unit | type |
| --- | --- | --- | --- |
| Organism\|Fat\|Specific blood flow rate | 0.267 | l/min/kg organ | rabbit specific |
| Organism\|Gonads\|Specific blood flow rate | 0.358 | l/min/kg organ | rabbit specific |
| Organism\|Gonads\|Volume | 0.005 | l | rabbit specific |
| Organism\|Hematocrit | 0.41 |  | rabbit specific |
| Organism\|Kidney\|GFR (specific) | 0.6 | l/min/kg organ | rabbit specific |
| Organism\|LargeIntestine\|Large intestinal transit time | 810 | min | rabbit specific |
| Organism\|LargeIntestine\|Mucosa\|Caecum\|Fraction mucosa | 0.589 |  | rabbit specific |
| Organism\|LargeIntestine\|Mucosa\|ColonAscendens\|Fraction mucosa | 0.589 |  | rabbit specific |
| Organism\|LargeIntestine\|Mucosa\|ColonDescendens\|Fraction mucosa | 0.589 |  | rabbit specific |
| Organism\|LargeIntestine\|Mucosa\|ColonSigmoid\|Fraction mucosa | 0.589 |  | rabbit specific |
| Organism\|LargeIntestine\|Mucosa\|ColonTransversum\|Fraction mucosa | 0.589 |  | rabbit specific |
| Organism\|LargeIntestine\|Mucosa\|Rectum\|Fraction mucosa | 0.589 |  | rabbit specific |
| Organism\|Liver\|Microsomal protein mass/g tissue | 0 | kg/kg | rabbit specific |
| Organism\|Lumen\|Caecum\|Distal radius | 0.2 | dm | rabbit specific |
| Organism\|Lumen\|Caecum\|Effective surface area enhancement factor | 2.0266 |  | rabbit specific |
| Organism\|Lumen\|Caecum\|Proximal radius | 0.15 | dm | rabbit specific |
| Organism\|Lumen\|ColonAscendens\|Distal radius | 0.05 | dm | rabbit specific |
| Organism\|Lumen\|ColonAscendens\|Effective surface area enhancement factor | 1.947 |  | rabbit specific |
| Organism\|Lumen\|ColonAscendens\|Length | 1 | dm | rabbit specific |
| Organism\|Lumen\|ColonAscendens\|pH | 7.2 |  | rabbit specific |
| Organism\|Lumen\|ColonAscendens\|Proximal radius | 0.05 | dm | rabbit specific |
| Organism\|Lumen\|ColonDescendens\|Distal radius | 0.05 | dm | rabbit specific |
| Organism\|Lumen\|ColonDescendens\|Effective surface area enhancement factor | 1.947 |  | rabbit specific |
| Organism\|Lumen\|ColonDescendens\|Length | 0.4 | dm | rabbit specific |
| Organism\|Lumen\|ColonDescendens\|pH | 7.2 |  | rabbit specific |
| Organism\|Lumen\|ColonDescendens\|Proximal radius | 0.05 | dm | rabbit specific |
| Organism\|Lumen\|ColonSigmoid\|Distal radius | 0.05 | dm | rabbit specific |
| Organism\|Lumen\|ColonSigmoid\|Effective surface area enhancement factor | 1.947 |  | rabbit specific |
| Organism\|Lumen\|ColonSigmoid\|Length | 10 | dm | rabbit specific |
| Organism\|Lumen\|ColonSigmoid\|pH | 7.2 |  | rabbit specific |
| Organism\|Lumen\|ColonSigmoid\|Proximal radius | 0.05 | dm | rabbit specific |
| Organism\|Lumen\|ColonTransversum\|Distal radius | 0.05 | dm | rabbit specific |
| Organism\|Lumen\|ColonTransversum\|Effective surface area enhancement factor | 1.947 |  | rabbit specific |
| Organism\|Lumen\|ColonTransversum\|Length | 2 | dm | rabbit specific |
| Organism\|Lumen\|ColonTransversum\|pH | 7.2 |  | rabbit specific |
| Organism\|Lumen\|ColonTransversum\|Proximal radius | 0.05 | dm | rabbit specific |
| Organism\|Lumen\|Duodenum\|Distal radius | 0.05 | dm | rabbit specific |
| Organism\|Lumen\|Duodenum\|Length | 6 | dm | rabbit specific |
| Organism\|Lumen\|Duodenum\|Microvilli factor | 1 |  | rabbit specific |
| Organism\|Lumen\|LowerIleum\|Distal radius | 0.05 | dm | rabbit specific |
| Organism\|Lumen\|LowerIleum\|Microvilli factor | 1 |  | rabbit specific |
| Organism\|Lumen\|LowerIleum\|pH | 7.5 |  | rabbit specific |
| Organism\|Lumen\|LowerIleum\|Proximal radius | 0.05 | dm | rabbit specific |
| Organism\|Lumen\|LowerJejunum\|Distal radius | 0.05 | dm | rabbit specific |
| Organism\|Lumen\|LowerJejunum\|Microvilli factor | 1 |  | rabbit specific |
| Organism\|Lumen\|LowerJejunum\|Proximal radius | 0.05 | dm | rabbit specific |
| Organism\|Lumen\|Rectum\|Distal radius | 0.05 | dm | rabbit specific |
| Organism\|Lumen\|Rectum\|Effective surface area enhancement factor | 1.947 |  | rabbit specific |
| Organism\|Lumen\|Rectum\|Length | 0.1 | dm | rabbit specific |
| Organism\|Lumen\|Rectum\|pH | 7.2 |  | rabbit specific |
| Organism\|Lumen\|Rectum\|Proximal radius | 0.05 | dm | rabbit specific |
| Organism\|Lumen\|Stomach\|Gastric emptying time | 30 | min | rabbit specific |
| Organism\|Lumen\|Stomach\|pH in fasted state | 1.9 |  | rabbit specific |
| Organism\|Lumen\|UpperIleum\|Distal radius | 0.05 | dm | rabbit specific |
| Organism\|Lumen\|UpperIleum\|Microvilli factor | 1 |  | rabbit specific |
| Organism\|Lumen\|UpperIleum\|Proximal radius | 0.05 | dm | rabbit specific |
| Organism\|Lumen\|UpperJejunum\|Distal radius | 0.05 | dm | rabbit specific |
| Organism\|Lumen\|UpperJejunum\|Microvilli factor | 1 |  | rabbit specific |
| Organism\|Lumen\|UpperJejunum\|Proximal radius | 0.05 | dm | rabbit specific |
| Organism\|Skin\|Volume | 0.2534 | l | rabbit specific |
| Organism\|Surface/Volume ratio (blood cells) | 200000 | 1/dm | rabbit specific |
| Organism\|ArterialBlood\|Volume | 0.05 | l | rabbit specific / distinct in each (of 7) species |
| Organism\|Bone\|Specific blood flow rate | 0.15 | l/min/kg organ | rabbit specific / distinct in each (of 7) species |
| Organism\|Bone\|Volume | 0.31 | l | rabbit specific / distinct in each (of 7) species |
| Organism\|Brain\|Specific blood flow rate | 0.678 | l/min/kg organ | rabbit specific / distinct in each (of 7) species |
| Organism\|Brain\|Volume | 0.014 | l | rabbit specific / distinct in each (of 7) species |
| Organism\|Fat\|Volume | 0.12 | l | rabbit specific / distinct in each (of 7) species |
| Organism\|Heart\|Specific blood flow rate | 3.2 | l/min/kg organ | rabbit specific / distinct in each (of 7) species |
| Organism\|Heart\|Volume | 0.006 | l | rabbit specific / distinct in each (of 7) species |
| Organism\|Kidney\|Specific blood flow rate | 6.154 | l/min/kg organ | rabbit specific / distinct in each (of 7) species |
| Organism\|Kidney\|Volume | 0.015 | l | rabbit specific / distinct in each (of 7) species |
| Organism\|LargeIntestine\|Specific blood flow rate | 0.47 | l/min/kg organ | rabbit specific / distinct in each (of 7) species |
| Organism\|LargeIntestine\|Volume | 0.03 | l | rabbit specific / distinct in each (of 7) species |
| Organism\|Liver\|Specific blood flow rate | 2.299 | l/min/kg organ | rabbit specific / distinct in each (of 7) species |
| Organism\|Liver\|Volume | 0.1 | l | rabbit specific / distinct in each (of 7) species |
| Organism\|Lumen\|Caecum\|Intestinal transit rate | 4.148 | 1/min | rabbit specific / distinct in each (of 7) species |
| Organism\|Lumen\|Caecum\|Length | 4.5 | dm | rabbit specific / distinct in each (of 7) species |
| Organism\|Lumen\|Caecum\|pH | 6.6 |  | rabbit specific / distinct in each (of 7) species |
| Organism\|Lumen\|ColonAscendens\|Intestinal transit rate | 18.667 | 1/min | rabbit specific / distinct in each (of 7) species |
| Organism\|Lumen\|ColonDescendens\|Intestinal transit rate | 46.667 | 1/min | rabbit specific / distinct in each (of 7) species |
| Organism\|Lumen\|ColonSigmoid\|Intestinal transit rate | 1.867 | 1/min | rabbit specific / distinct in each (of 7) species |
| Organism\|Lumen\|ColonTransversum\|Intestinal transit rate | 7 | 1/min | rabbit specific / distinct in each (of 7) species |
| Organism\|Lumen\|Duodenum\|Effective surface area enhancement factor | 584.97 |  | rabbit specific / distinct in each (of 7) species |
| Organism\|Lumen\|Duodenum\|Intestinal transit rate | 5 | 1/min | rabbit specific / distinct in each (of 7) species |
| Organism\|Lumen\|Duodenum\|pH | 6 |  | rabbit specific / distinct in each (of 7) species |
| Organism\|Lumen\|Duodenum\|Proximal radius | 0.04 | dm | rabbit specific / distinct in each (of 7) species |
| Organism\|Lumen\|LowerIleum\|Effective surface area enhancement factor | 581.36 |  | rabbit specific / distinct in each (of 7) species |
| Organism\|Lumen\|LowerIleum\|Intestinal transit rate | 5 | 1/min | rabbit specific / distinct in each (of 7) species |
| Organism\|Lumen\|LowerIleum\|Length | 6 | dm | rabbit specific / distinct in each (of 7) species |
| Organism\|Lumen\|LowerJejunum\|Effective surface area enhancement factor | 592 |  | rabbit specific / distinct in each (of 7) species |
| Organism\|Lumen\|LowerJejunum\|Intestinal transit rate | 5 | 1/min | rabbit specific / distinct in each (of 7) species |
| Organism\|Lumen\|LowerJejunum\|Length | 6 | dm | rabbit specific / distinct in each (of 7) species |
| Organism\|Lumen\|LowerJejunum\|pH | 6.8 |  | rabbit specific / distinct in each (of 7) species |
| Organism\|Lumen\|Rectum\|Intestinal transit rate | 186.667 | 1/min | rabbit specific / distinct in each (of 7) species |
| Organism\|Lumen\|Stomach\|Distal radius | 0.15 | dm | rabbit specific / distinct in each (of 7) species |
| Organism\|Lumen\|Stomach\|Length | 1.4 | dm | rabbit specific / distinct in each (of 7) species |
| Organism\|Lumen\|Stomach\|Proximal radius | 0.15 | dm | rabbit specific / distinct in each (of 7) species |
| Organism\|Lumen\|UpperIleum\|Effective surface area enhancement factor | 584.36 |  | rabbit specific / distinct in each (of 7) species |
| Organism\|Lumen\|UpperIleum\|Intestinal transit rate | 5 | 1/min | rabbit specific / distinct in each (of 7) species |
| Organism\|Lumen\|UpperIleum\|Length | 6 | dm | rabbit specific / distinct in each (of 7) species |
| Organism\|Lumen\|UpperIleum\|pH | 8 |  | rabbit specific / distinct in each (of 7) species |
| Organism\|Lumen\|UpperJejunum\|Effective surface area enhancement factor | 568.64 |  | rabbit specific / distinct in each (of 7) species |
| Organism\|Lumen\|UpperJejunum\|Intestinal transit rate | 5 | 1/min | rabbit specific / distinct in each (of 7) species |
| Organism\|Lumen\|UpperJejunum\|Length | 6 | dm | rabbit specific / distinct in each (of 7) species |
| Organism\|Lumen\|UpperJejunum\|pH | 6.8 |  | rabbit specific / distinct in each (of 7) species |
| Organism\|Lung\|Volume | 0.017 | l | rabbit specific / distinct in each (of 7) species |
| Organism\|MeanBW | 2.497 | kg | rabbit specific / distinct in each (of 7) species |
| Organism\|Muscle\|Specific blood flow rate | 0.115 | l/min/kg organ | rabbit specific / distinct in each (of 7) species |
| Organism\|Muscle\|Volume | 1.35 | l | rabbit specific / distinct in each (of 7) species |
| Organism\|Pancreas\|Specific blood flow rate | 1 | l/min/kg organ | rabbit specific / distinct in each (of 7) species |
| Organism\|Pancreas\|Volume | 0.0036 | l | rabbit specific / distinct in each (of 7) species |
| Organism\|PortalVein\|Volume | 0.027 | l | rabbit specific / distinct in each (of 7) species |
| Organism\|Skin\|Specific blood flow rate | 0.158 | l/min/kg organ | rabbit specific / distinct in each (of 7) species |
| Organism\|SmallIntestine\|Small intestinal transit time | 90 | min | rabbit specific / distinct in each (of 7) species |
| Organism\|SmallIntestine\|Specific blood flow rate | 0.92 | l/min/kg organ | rabbit specific / distinct in each (of 7) species |
| Organism\|SmallIntestine\|Volume | 0.06 | l | rabbit specific / distinct in each (of 7) species |
| Organism\|Spleen\|Specific blood flow rate | 9 | l/min/kg organ | rabbit specific / distinct in each (of 7) species |
| Organism\|Spleen\|Volume | 0.001 | l | rabbit specific / distinct in each (of 7) species |
| Organism\|Stomach\|Specific blood flow rate | 1.05 | l/min/kg organ | rabbit specific / distinct in each (of 7) species |
| Organism\|Stomach\|Volume | 0.02 | l | rabbit specific / distinct in each (of 7) species |
| Organism\|VenousBlood\|Volume | 0.115 | l | rabbit specific / distinct in each (of 7) species |
| Organism\|Acidic phospholipids (blood cells) [mg/g] - RR | 0.5 |  | shared between all (7) species |
| Organism\|ArterialBlood\|Density (tissue) | 1 | kg/dm³ | shared between all (7) species |
| Organism\|ArterialBlood\|Fraction vascular | 1 |  | shared between all (7) species |
| Organism\|ArterialBlood\|Peripheral blood flow fraction | 0 |  | shared between all (7) species |
| Organism\|Bone\|Acidic phospholipids [mg/g] - RR | 0.67 |  | shared between all (7) species |
| Organism\|Bone\|Albumin ratio (tissue/plasma) | 0.1 |  | shared between all (7) species |
| Organism\|Bone\|Albumin ratio (tissue/plasma)-PT | 0.5 |  | shared between all (7) species |
| Organism\|Bone\|Allometric scale factor | 0.75 |  | shared between all (7) species |
| Organism\|Bone\|Density (tissue) | 1 | kg/dm³ | shared between all (7) species |
| Organism\|Bone\|Flow fraction via large pores | 0.8 |  | shared between all (7) species |
| Organism\|Bone\|Fraction interstitial | 0.1 |  | shared between all (7) species |
| Organism\|Bone\|Fraction of blood for sampling | 1 |  | shared between all (7) species |
| Organism\|Bone\|Fraction vascular | 0.041 |  | shared between all (7) species |
| Organism\|Bone\|Hydraulic conductivity | 3.89E-11 | l/min/(kg*dm/min²) | shared between all (7) species |
| Organism\|Bone\|Intracellular\|pH | 7 |  | shared between all (7) species |
| Organism\|Bone\|Lipoprotein ratio (tissue/plasma) | 0.05 |  | shared between all (7) species |
| Organism\|Bone\|Peripheral blood flow fraction | 0 |  | shared between all (7) species |
| Organism\|Bone\|Radius (large pores) | 3.3E-07 | dm | shared between all (7) species |
| Organism\|Bone\|Radius (small pores) | 9E-08 | dm | shared between all (7) species |
| Organism\|Bone\|Vf (acidic phospholipids)-WS | 0.0008 |  | shared between all (7) species |
| Organism\|Bone\|Vf (extracellular water)-RR | 0.1 |  | shared between all (7) species |
| Organism\|Bone\|Vf (intracellular water)-RR | 0.317 |  | shared between all (7) species |
| Organism\|Bone\|Vf (lipid) | 0.268 |  | shared between all (7) species |
| Organism\|Bone\|Vf (neutral lipid)-RR | 0.0174 |  | shared between all (7) species |
| Organism\|Bone\|Vf (neutral lipid)-WS | 0.017 |  | shared between all (7) species |
| Organism\|Bone\|Vf (neutral phospholipid)-RR | 0.0016 |  | shared between all (7) species |
| Organism\|Bone\|Vf (neutral phospholipid, plasma)-WS | 0.0022 |  | shared between all (7) species |
| Organism\|Bone\|Vf (protein) | 0.268 |  | shared between all (7) species |
| Organism\|Bone\|Vf (protein)-WS | 0.21 |  | shared between all (7) species |
| Organism\|Bone\|Vf (water) | 0.465 |  | shared between all (7) species |
| Organism\|Bone\|Vf (water)-WS | 0.26 |  | shared between all (7) species |
| Organism\|Brain\|Acidic phospholipids [mg/g] - RR | 0.4 |  | shared between all (7) species |
| Organism\|Brain\|Albumin ratio (tissue/plasma) | 0.048 |  | shared between all (7) species |
| Organism\|Brain\|Albumin ratio (tissue/plasma)-PT | 0.5 |  | shared between all (7) species |
| Organism\|Brain\|Allometric scale factor | 0.75 |  | shared between all (7) species |
| Organism\|Brain\|Density (tissue) | 1 | kg/dm³ | shared between all (7) species |
| Organism\|Brain\|Flow fraction via large pores | 0.05 |  | shared between all (7) species |
| Organism\|Brain\|Fraction interstitial | 0.004 |  | shared between all (7) species |
| Organism\|Brain\|Fraction of blood for sampling | 1 |  | shared between all (7) species |
| Organism\|Brain\|Fraction vascular | 0.037 |  | shared between all (7) species |
| Organism\|Brain\|Hydraulic conductivity | 5E-14 | l/min/(kg*dm/min²) | shared between all (7) species |
| Organism\|Brain\|Intracellular\|pH | 7.1 |  | shared between all (7) species |
| Organism\|Brain\|Lipoprotein ratio (tissue/plasma) | 0.041 |  | shared between all (7) species |
| Organism\|Brain\|Radius (large pores) | 2.5E-07 | dm | shared between all (7) species |
| Organism\|Brain\|Radius (small pores) | 4.5E-08 | dm | shared between all (7) species |
| Organism\|Brain\|Vf (acidic phospholipids)-WS | 0.0143 |  | shared between all (7) species |
| Organism\|Brain\|Vf (extracellular water)-RR | 0.162 |  | shared between all (7) species |
| Organism\|Brain\|Vf (intracellular water)-RR | 0.591 |  | shared between all (7) species |
| Organism\|Brain\|Vf (lipid) | 0.11 |  | shared between all (7) species |
| Organism\|Brain\|Vf (neutral lipid)-RR | 0.0391 |  | shared between all (7) species |
| Organism\|Brain\|Vf (neutral lipid)-WS | 0.0429 |  | shared between all (7) species |
| Organism\|Brain\|Vf (neutral phospholipid)-RR | 0.0015 |  | shared between all (7) species |
| Organism\|Brain\|Vf (neutral phospholipid, plasma)-WS | 0.0528 |  | shared between all (7) species |
| Organism\|Brain\|Vf (protein) | 0.081 |  | shared between all (7) species |
| Organism\|Brain\|Vf (protein)-WS | 0.08 |  | shared between all (7) species |
| Organism\|Brain\|Vf (water) | 0.808 |  | shared between all (7) species |
| Organism\|Brain\|Vf (water)-WS | 0.79 |  | shared between all (7) species |
| Organism\|Fat\|Acidic phospholipids [mg/g] - RR | 0.4 |  | shared between all (7) species |
| Organism\|Fat\|Albumin ratio (tissue/plasma) | 0.049 |  | shared between all (7) species |
| Organism\|Fat\|Albumin ratio (tissue/plasma)-PT | 0 |  | shared between all (7) species |
| Organism\|Fat\|Allometric scale factor | 0.75 |  | shared between all (7) species |
| Organism\|Fat\|Density (tissue) | 1 | kg/dm³ | shared between all (7) species |
| Organism\|Fat\|Flow fraction via large pores | 0.05 |  | shared between all (7) species |
| Organism\|Fat\|Fraction interstitial | 0.135 |  | shared between all (7) species |
| Organism\|Fat\|Fraction of blood for sampling | 1 |  | shared between all (7) species |
| Organism\|Fat\|Fraction vascular | 0.01 |  | shared between all (7) species |
| Organism\|Fat\|Hydraulic conductivity | 4.17E-12 | l/min/(kg*dm/min²) | shared between all (7) species |
| Organism\|Fat\|Intracellular\|pH | 7.1 |  | shared between all (7) species |
| Organism\|Fat\|Lipoprotein ratio (tissue/plasma) | 0.068 |  | shared between all (7) species |
| Organism\|Fat\|Peripheral blood flow fraction | 0 |  | shared between all (7) species |
| Organism\|Fat\|Radius (large pores) | 2.5E-07 | dm | shared between all (7) species |
| Organism\|Fat\|Radius (small pores) | 4.5E-08 | dm | shared between all (7) species |
| Organism\|Fat\|Vf (acidic phospholipids)-WS | 0.000552 |  | shared between all (7) species |
| Organism\|Fat\|Vf (extracellular water)-RR | 0.135 |  | shared between all (7) species |
| Organism\|Fat\|Vf (lipid) | 0.8 |  | shared between all (7) species |
| Organism\|Fat\|Vf (neutral lipid)-PT | 0.853 |  | shared between all (7) species |
| Organism\|Fat\|Vf (neutral lipid)-RR | 0.853 |  | shared between all (7) species |
| Organism\|Fat\|Vf (neutral lipid)-WS | 0.92 |  | shared between all (7) species |
| Organism\|Fat\|Vf (neutral phospholipid)-RR | 0.0016 |  | shared between all (7) species |
| Organism\|Fat\|Vf (neutral phospholipid, plasma)-WS | 0.002024 |  | shared between all (7) species |
| Organism\|Fat\|Vf (phospholipid)-PT | 0.002 |  | shared between all (7) species |
| Organism\|Fat\|Vf (protein) | 0.05 |  | shared between all (7) species |
| Organism\|Fat\|Vf (protein)-WS | 0.06 |  | shared between all (7) species |
| Organism\|Fat\|Vf (water) | 0.15 |  | shared between all (7) species |
| Organism\|Fat\|Vf (water)-WS | 0.03 |  | shared between all (7) species |
| Organism\|Fraction endosomal (global) | 0.2 |  | shared between all (7) species |
| Organism\|Fraction of endosomal uptake from plasma (global) | 0.5 |  | shared between all (7) species |
| Organism\|Fraction recycled to plasma (global) | 0.5 |  | shared between all (7) species |
| Organism\|Gonads\|Acidic phospholipids [mg/g] - RR | 2.45 |  | shared between all (7) species |
| Organism\|Gonads\|Albumin ratio (tissue/plasma) | 0.048 |  | shared between all (7) species |
| Organism\|Gonads\|Albumin ratio (tissue/plasma)-PT | 0.5 |  | shared between all (7) species |
| Organism\|Gonads\|Allometric scale factor | 0.75 |  | shared between all (7) species |
| Organism\|Gonads\|Density (tissue) | 1 | kg/dm³ | shared between all (7) species |
| Organism\|Gonads\|Flow fraction via large pores | 0.05 |  | shared between all (7) species |
| Organism\|Gonads\|Fraction interstitial | 0.069 |  | shared between all (7) species |
| Organism\|Gonads\|Fraction of blood for sampling | 1 |  | shared between all (7) species |
| Organism\|Gonads\|Fraction vascular | 0.14 |  | shared between all (7) species |
| Organism\|Gonads\|Hydraulic conductivity | 1.67E-12 | l/min/(kg*dm/min²) | shared between all (7) species |
| Organism\|Gonads\|Intracellular\|pH | 7 |  | shared between all (7) species |
| Organism\|Gonads\|Lipoprotein ratio (tissue/plasma) | 0.041 |  | shared between all (7) species |
| Organism\|Gonads\|Radius (large pores) | 2.5E-07 | dm | shared between all (7) species |
| Organism\|Gonads\|Radius (small pores) | 4.5E-08 | dm | shared between all (7) species |
| Organism\|Gonads\|Vf (acidic phospholipids)-WS | 0.0054 |  | shared between all (7) species |
| Organism\|Gonads\|Vf (extracellular water)-RR | 0.03 |  | shared between all (7) species |
| Organism\|Gonads\|Vf (intracellular water)-RR | 0.83 |  | shared between all (7) species |
| Organism\|Gonads\|Vf (lipid) | 0.031 |  | shared between all (7) species |
| Organism\|Gonads\|Vf (neutral lipid)-PT | 0.0048 |  | shared between all (7) species |
| Organism\|Gonads\|Vf (neutral lipid)-RR | 0.0048 |  | shared between all (7) species |
| Organism\|Gonads\|Vf (neutral lipid)-WS | 0 |  | shared between all (7) species |
| Organism\|Gonads\|Vf (neutral phospholipid)-RR | 0.0116 |  | shared between all (7) species |
| Organism\|Gonads\|Vf (neutral phospholipid, plasma)-WS | 0.0249 |  | shared between all (7) species |
| Organism\|Gonads\|Vf (phospholipid)-PT | 0.01405 |  | shared between all (7) species |
| Organism\|Gonads\|Vf (protein) | 0.12 |  | shared between all (7) species |
| Organism\|Gonads\|Vf (protein)-WS | 0.13 |  | shared between all (7) species |
| Organism\|Gonads\|Vf (water) | 0.8 |  | shared between all (7) species |
| Organism\|Gonads\|Vf (water)-PT | 0.8 |  | shared between all (7) species |
| Organism\|Gonads\|Vf (water)-WS | 0.78 |  | shared between all (7) species |
| Organism\|Heart\|Acidic phospholipids [mg/g] - RR | 2.25 |  | shared between all (7) species |
| Organism\|Heart\|Albumin ratio (tissue/plasma) | 0.157 |  | shared between all (7) species |
| Organism\|Heart\|Albumin ratio (tissue/plasma)-PT | 0.5 |  | shared between all (7) species |
| Organism\|Heart\|Allometric scale factor | 0.75 |  | shared between all (7) species |
| Organism\|Heart\|Density (tissue) | 1 | kg/dm³ | shared between all (7) species |
| Organism\|Heart\|Flow fraction via large pores | 0.05 |  | shared between all (7) species |
| Organism\|Heart\|Fraction interstitial | 0.1 |  | shared between all (7) species |
| Organism\|Heart\|Fraction of blood for sampling | 1 |  | shared between all (7) species |
| Organism\|Heart\|Fraction vascular | 0.262 |  | shared between all (7) species |
| Organism\|Heart\|Hydraulic conductivity | 1.43E-11 | l/min/(kg*dm/min²) | shared between all (7) species |
| Organism\|Heart\|Intracellular\|pH | 7.1 |  | shared between all (7) species |
| Organism\|Heart\|Lipoprotein ratio (tissue/plasma) | 0.16 |  | shared between all (7) species |
| Organism\|Heart\|Radius (large pores) | 2.5E-07 | dm | shared between all (7) species |
| Organism\|Heart\|Radius (small pores) | 4.5E-08 | dm | shared between all (7) species |
| Organism\|Heart\|Vf (acidic phospholipids)-WS | 0.0099 |  | shared between all (7) species |
| Organism\|Heart\|Vf (extracellular water)-RR | 0.32 |  | shared between all (7) species |
| Organism\|Heart\|Vf (intracellular water)-RR | 0.248 |  | shared between all (7) species |
| Organism\|Heart\|Vf (lipid) | 0.1 |  | shared between all (7) species |
| Organism\|Heart\|Vf (neutral lipid)-RR | 0.0135 |  | shared between all (7) species |
| Organism\|Heart\|Vf (neutral lipid)-WS | 0.0528 |  | shared between all (7) species |
| Organism\|Heart\|Vf (neutral phospholipid)-RR | 0.0106 |  | shared between all (7) species |
| Organism\|Heart\|Vf (neutral phospholipid, plasma)-WS | 0.0473 |  | shared between all (7) species |
| Organism\|Heart\|Vf (protein) | 0.168 |  | shared between all (7) species |
| Organism\|Heart\|Vf (protein)-WS | 0.19 |  | shared between all (7) species |
| Organism\|Heart\|Vf (water) | 0.731 |  | shared between all (7) species |
| Organism\|Heart\|Vf (water)-WS | 0.7 |  | shared between all (7) species |
| Organism\|Kidney\|Acidic phospholipids [mg/g] - RR | 5.03 |  | shared between all (7) species |
| Organism\|Kidney\|Albumin ratio (tissue/plasma) | 0.13 |  | shared between all (7) species |
| Organism\|Kidney\|Albumin ratio (tissue/plasma)-PT | 0.5 |  | shared between all (7) species |
| Organism\|Kidney\|Allometric scale factor | 0.75 |  | shared between all (7) species |
| Organism\|Kidney\|Density (tissue) | 1 | kg/dm³ | shared between all (7) species |
| Organism\|Kidney\|fGFRpremat | 0.258 |  | shared between all (7) species |
| Organism\|Kidney\|Flow fraction via large pores | 0.05 |  | shared between all (7) species |
| Organism\|Kidney\|Fraction interstitial | 0.2 |  | shared between all (7) species |
| Organism\|Kidney\|Fraction of blood for sampling | 1 |  | shared between all (7) species |
| Organism\|Kidney\|Fraction vascular | 0.105 |  | shared between all (7) species |
| Organism\|Kidney\|Hydraulic conductivity | 1.54E-10 | l/min/(kg*dm/min²) | shared between all (7) species |
| Organism\|Kidney\|Intracellular\|pH | 7.22 |  | shared between all (7) species |
| Organism\|Kidney\|Lipoprotein ratio (tissue/plasma) | 0.137 |  | shared between all (7) species |
| Organism\|Kidney\|Radius (large pores) | 2.5E-07 | dm | shared between all (7) species |
| Organism\|Kidney\|Radius (small pores) | 4.5E-08 | dm | shared between all (7) species |
| Organism\|Kidney\|Urine\|Volume | 1 | l | shared between all (7) species |
| Organism\|Kidney\|Vf (acidic phospholipids)-WS | 0.0078 |  | shared between all (7) species |
| Organism\|Kidney\|Vf (extracellular water)-RR | 0.273 |  | shared between all (7) species |
| Organism\|Kidney\|Vf (intracellular water)-RR | 0.399 |  | shared between all (7) species |
| Organism\|Kidney\|Vf (lipid) | 0.052 |  | shared between all (7) species |
| Organism\|Kidney\|Vf (neutral lipid)-RR | 0.0121 |  | shared between all (7) species |
| Organism\|Kidney\|Vf (neutral lipid)-WS | 0.0156 |  | shared between all (7) species |
| Organism\|Kidney\|Vf (neutral phospholipid)-RR | 0.024 |  | shared between all (7) species |
| Organism\|Kidney\|Vf (neutral phospholipid, plasma)-WS | 0.0366 |  | shared between all (7) species |
| Organism\|Kidney\|Vf (protein) | 0.171 |  | shared between all (7) species |
| Organism\|Kidney\|Vf (protein)-WS | 0.21 |  | shared between all (7) species |
| Organism\|Kidney\|Vf (water) | 0.774 |  | shared between all (7) species |
| Organism\|Kidney\|Vf (water)-WS | 0.73 |  | shared between all (7) species |
| Organism\|LargeIntestine\|Acidic phospholipids [mg/g] - RR | 2.41 |  | shared between all (7) species |
| Organism\|LargeIntestine\|Albumin ratio (tissue/plasma) | 0.158 |  | shared between all (7) species |
| Organism\|LargeIntestine\|Albumin ratio (tissue/plasma)-PT | 0.5 |  | shared between all (7) species |
| Organism\|LargeIntestine\|Allometric scale factor | 0.75 |  | shared between all (7) species |
| Organism\|LargeIntestine\|Density (tissue) | 1 | kg/dm³ | shared between all (7) species |
| Organism\|LargeIntestine\|Flow fraction via large pores | 0.05 |  | shared between all (7) species |
| Organism\|LargeIntestine\|Fraction interstitial | 0.094 |  | shared between all (7) species |
| Organism\|LargeIntestine\|Fraction of blood for sampling | 1 |  | shared between all (7) species |
| Organism\|LargeIntestine\|Fraction vascular | 0.024 |  | shared between all (7) species |
| Organism\|LargeIntestine\|Hydraulic conductivity | 1.87E-10 | l/min/(kg*dm/min²) | shared between all (7) species |
| Organism\|LargeIntestine\|Intracellular\|pH | 7.4 |  | shared between all (7) species |
| Organism\|LargeIntestine\|Lipoprotein ratio (tissue/plasma) | 0.141 |  | shared between all (7) species |
| Organism\|LargeIntestine\|Mucosa blood flow fraction | 0.75 |  | shared between all (7) species |
| Organism\|LargeIntestine\|Mucosa\|Caecum\|Acidic phospholipids [mg/g] - RR | 2.41 |  | shared between all (7) species |
| Organism\|LargeIntestine\|Mucosa\|Caecum\|Albumin ratio (tissue/plasma) | 0.158 |  | shared between all (7) species |
| Organism\|LargeIntestine\|Mucosa\|Caecum\|Albumin ratio (tissue/plasma)-PT | 0.5 |  | shared between all (7) species |
| Organism\|LargeIntestine\|Mucosa\|Caecum\|Density (tissue) | 1 | kg/dm³ | shared between all (7) species |
| Organism\|LargeIntestine\|Mucosa\|Caecum\|Flow fraction via large pores | 0.05 |  | shared between all (7) species |
| Organism\|LargeIntestine\|Mucosa\|Caecum\|Fraction of blood for sampling | 1 |  | shared between all (7) species |
| Organism\|LargeIntestine\|Mucosa\|Caecum\|Hydraulic conductivity | 1.87E-10 | l/min/(kg*dm/min²) | shared between all (7) species |
| Organism\|LargeIntestine\|Mucosa\|Caecum\|Intracellular\|pH | 7.3 |  | shared between all (7) species |
| Organism\|LargeIntestine\|Mucosa\|Caecum\|Lipoprotein ratio (tissue/plasma) | 0.141 |  | shared between all (7) species |
| Organism\|LargeIntestine\|Mucosa\|Caecum\|Radius (large pores) | 2.5E-07 | dm | shared between all (7) species |
| Organism\|LargeIntestine\|Mucosa\|Caecum\|Radius (small pores) | 4.5E-08 | dm | shared between all (7) species |
| Organism\|LargeIntestine\|Mucosa\|Caecum\|Vf (acidic phospholipids)-WS | 0.0035 |  | shared between all (7) species |
| Organism\|LargeIntestine\|Mucosa\|Caecum\|Vf (extracellular water)-RR | 0.282 |  | shared between all (7) species |
| Organism\|LargeIntestine\|Mucosa\|Caecum\|Vf (intracellular water)-RR | 0.456 |  | shared between all (7) species |
| Organism\|LargeIntestine\|Mucosa\|Caecum\|Vf (lipid) | 0.06 |  | shared between all (7) species |
| Organism\|LargeIntestine\|Mucosa\|Caecum\|Vf (neutral lipid)-RR | 0.0375 |  | shared between all (7) species |
| Organism\|LargeIntestine\|Mucosa\|Caecum\|Vf (neutral lipid)-WS | 0.0483 |  | shared between all (7) species |
| Organism\|LargeIntestine\|Mucosa\|Caecum\|Vf (neutral phospholipid)-RR | 0.0124 |  | shared between all (7) species |
| Organism\|LargeIntestine\|Mucosa\|Caecum\|Vf (neutral phospholipid, plasma)-WS | 0.0182 |  | shared between all (7) species |
| Organism\|LargeIntestine\|Mucosa\|Caecum\|Vf (protein) | 0.08 |  | shared between all (7) species |
| Organism\|LargeIntestine\|Mucosa\|Caecum\|Vf (protein)-WS | 0.15 |  | shared between all (7) species |
| Organism\|LargeIntestine\|Mucosa\|Caecum\|Vf (water) | 0.8 |  | shared between all (7) species |
| Organism\|LargeIntestine\|Mucosa\|Caecum\|Vf (water)-WS | 0.78 |  | shared between all (7) species |
| Organism\|LargeIntestine\|Mucosa\|ColonAscendens\|Acidic phospholipids [mg/g] - RR | 2.41 |  | shared between all (7) species |
| Organism\|LargeIntestine\|Mucosa\|ColonAscendens\|Albumin ratio (tissue/plasma) | 0.158 |  | shared between all (7) species |
| Organism\|LargeIntestine\|Mucosa\|ColonAscendens\|Albumin ratio (tissue/plasma)-PT | 0.5 |  | shared between all (7) species |
| Organism\|LargeIntestine\|Mucosa\|ColonAscendens\|Density (tissue) | 1 | kg/dm³ | shared between all (7) species |
| Organism\|LargeIntestine\|Mucosa\|ColonAscendens\|Flow fraction via large pores | 0.05 |  | shared between all (7) species |
| Organism\|LargeIntestine\|Mucosa\|ColonAscendens\|Fraction of blood for sampling | 1 |  | shared between all (7) species |
| Organism\|LargeIntestine\|Mucosa\|ColonAscendens\|Hydraulic conductivity | 1.87E-10 | l/min/(kg*dm/min²) | shared between all (7) species |
| Organism\|LargeIntestine\|Mucosa\|ColonAscendens\|Intracellular\|pH | 7.3 |  | shared between all (7) species |
| Organism\|LargeIntestine\|Mucosa\|ColonAscendens\|Lipoprotein ratio (tissue/plasma) | 0.141 |  | shared between all (7) species |
| Organism\|LargeIntestine\|Mucosa\|ColonAscendens\|Radius (large pores) | 2.5E-07 | dm | shared between all (7) species |
| Organism\|LargeIntestine\|Mucosa\|ColonAscendens\|Radius (small pores) | 4.5E-08 | dm | shared between all (7) species |
| Organism\|LargeIntestine\|Mucosa\|ColonAscendens\|Vf (acidic phospholipids)-WS | 0.0035 |  | shared between all (7) species |
| Organism\|LargeIntestine\|Mucosa\|ColonAscendens\|Vf (extracellular water)-RR | 0.282 |  | shared between all (7) species |
| Organism\|LargeIntestine\|Mucosa\|ColonAscendens\|Vf (intracellular water)-RR | 0.456 |  | shared between all (7) species |
| Organism\|LargeIntestine\|Mucosa\|ColonAscendens\|Vf (lipid) | 0.06 |  | shared between all (7) species |
| Organism\|LargeIntestine\|Mucosa\|ColonAscendens\|Vf (neutral lipid)-RR | 0.0375 |  | shared between all (7) species |
| Organism\|LargeIntestine\|Mucosa\|ColonAscendens\|Vf (neutral lipid)-WS | 0.0483 |  | shared between all (7) species |
| Organism\|LargeIntestine\|Mucosa\|ColonAscendens\|Vf (neutral phospholipid)-RR | 0.0124 |  | shared between all (7) species |
| Organism\|LargeIntestine\|Mucosa\|ColonAscendens\|Vf (neutral phospholipid, plasma)-WS | 0.0182 |  | shared between all (7) species |
| Organism\|LargeIntestine\|Mucosa\|ColonAscendens\|Vf (protein) | 0.08 |  | shared between all (7) species |
| Organism\|LargeIntestine\|Mucosa\|ColonAscendens\|Vf (protein)-WS | 0.15 |  | shared between all (7) species |
| Organism\|LargeIntestine\|Mucosa\|ColonAscendens\|Vf (water) | 0.8 |  | shared between all (7) species |
| Organism\|LargeIntestine\|Mucosa\|ColonAscendens\|Vf (water)-WS | 0.78 |  | shared between all (7) species |
| Organism\|LargeIntestine\|Mucosa\|ColonDescendens\|Acidic phospholipids [mg/g] - RR | 2.41 |  | shared between all (7) species |
| Organism\|LargeIntestine\|Mucosa\|ColonDescendens\|Albumin ratio (tissue/plasma) | 0.158 |  | shared between all (7) species |
| Organism\|LargeIntestine\|Mucosa\|ColonDescendens\|Albumin ratio (tissue/plasma)-PT | 0.5 |  | shared between all (7) species |
| Organism\|LargeIntestine\|Mucosa\|ColonDescendens\|Density (tissue) | 1 | kg/dm³ | shared between all (7) species |
| Organism\|LargeIntestine\|Mucosa\|ColonDescendens\|Flow fraction via large pores | 0.05 |  | shared between all (7) species |
| Organism\|LargeIntestine\|Mucosa\|ColonDescendens\|Fraction of blood for sampling | 1 |  | shared between all (7) species |
| Organism\|LargeIntestine\|Mucosa\|ColonDescendens\|Hydraulic conductivity | 1.87E-10 | l/min/(kg*dm/min²) | shared between all (7) species |
| Organism\|LargeIntestine\|Mucosa\|ColonDescendens\|Intracellular\|pH | 7.3 |  | shared between all (7) species |
| Organism\|LargeIntestine\|Mucosa\|ColonDescendens\|Lipoprotein ratio (tissue/plasma) | 0.141 |  | shared between all (7) species |
| Organism\|LargeIntestine\|Mucosa\|ColonDescendens\|Radius (large pores) | 2.5E-07 | dm | shared between all (7) species |
| Organism\|LargeIntestine\|Mucosa\|ColonDescendens\|Radius (small pores) | 4.5E-08 | dm | shared between all (7) species |
| Organism\|LargeIntestine\|Mucosa\|ColonDescendens\|Vf (acidic phospholipids)-WS | 0.0035 |  | shared between all (7) species |
| Organism\|LargeIntestine\|Mucosa\|ColonDescendens\|Vf (extracellular water)-RR | 0.282 |  | shared between all (7) species |
| Organism\|LargeIntestine\|Mucosa\|ColonDescendens\|Vf (intracellular water)-RR | 0.456 |  | shared between all (7) species |
| Organism\|LargeIntestine\|Mucosa\|ColonDescendens\|Vf (lipid) | 0.06 |  | shared between all (7) species |
| Organism\|LargeIntestine\|Mucosa\|ColonDescendens\|Vf (neutral lipid)-RR | 0.0375 |  | shared between all (7) species |
| Organism\|LargeIntestine\|Mucosa\|ColonDescendens\|Vf (neutral lipid)-WS | 0.0483 |  | shared between all (7) species |
| Organism\|LargeIntestine\|Mucosa\|ColonDescendens\|Vf (neutral phospholipid)-RR | 0.0124 |  | shared between all (7) species |
| Organism\|LargeIntestine\|Mucosa\|ColonDescendens\|Vf (neutral phospholipid, plasma)-WS | 0.0182 |  | shared between all (7) species |
| Organism\|LargeIntestine\|Mucosa\|ColonDescendens\|Vf (protein) | 0.08 |  | shared between all (7) species |
| Organism\|LargeIntestine\|Mucosa\|ColonDescendens\|Vf (protein)-WS | 0.15 |  | shared between all (7) species |
| Organism\|LargeIntestine\|Mucosa\|ColonDescendens\|Vf (water) | 0.8 |  | shared between all (7) species |
| Organism\|LargeIntestine\|Mucosa\|ColonDescendens\|Vf (water)-WS | 0.78 |  | shared between all (7) species |
| Organism\|LargeIntestine\|Mucosa\|ColonSigmoid\|Acidic phospholipids [mg/g] - RR | 2.41 |  | shared between all (7) species |
| Organism\|LargeIntestine\|Mucosa\|ColonSigmoid\|Albumin ratio (tissue/plasma) | 0.158 |  | shared between all (7) species |
| Organism\|LargeIntestine\|Mucosa\|ColonSigmoid\|Albumin ratio (tissue/plasma)-PT | 0.5 |  | shared between all (7) species |
| Organism\|LargeIntestine\|Mucosa\|ColonSigmoid\|Density (tissue) | 1 | kg/dm³ | shared between all (7) species |
| Organism\|LargeIntestine\|Mucosa\|ColonSigmoid\|Flow fraction via large pores | 0.05 |  | shared between all (7) species |
| Organism\|LargeIntestine\|Mucosa\|ColonSigmoid\|Fraction of blood for sampling | 1 |  | shared between all (7) species |
| Organism\|LargeIntestine\|Mucosa\|ColonSigmoid\|Hydraulic conductivity | 1.87E-10 | l/min/(kg*dm/min²) | shared between all (7) species |
| Organism\|LargeIntestine\|Mucosa\|ColonSigmoid\|Intracellular\|pH | 7.3 |  | shared between all (7) species |
| Organism\|LargeIntestine\|Mucosa\|ColonSigmoid\|Lipoprotein ratio (tissue/plasma) | 0.141 |  | shared between all (7) species |
| Organism\|LargeIntestine\|Mucosa\|ColonSigmoid\|Radius (large pores) | 2.5E-07 | dm | shared between all (7) species |
| Organism\|LargeIntestine\|Mucosa\|ColonSigmoid\|Radius (small pores) | 4.5E-08 | dm | shared between all (7) species |
| Organism\|LargeIntestine\|Mucosa\|ColonSigmoid\|Vf (acidic phospholipids)-WS | 0.0035 |  | shared between all (7) species |
| Organism\|LargeIntestine\|Mucosa\|ColonSigmoid\|Vf (extracellular water)-RR | 0.282 |  | shared between all (7) species |
| Organism\|LargeIntestine\|Mucosa\|ColonSigmoid\|Vf (intracellular water)-RR | 0.456 |  | shared between all (7) species |
| Organism\|LargeIntestine\|Mucosa\|ColonSigmoid\|Vf (lipid) | 0.06 |  | shared between all (7) species |
| Organism\|LargeIntestine\|Mucosa\|ColonSigmoid\|Vf (neutral lipid)-RR | 0.0375 |  | shared between all (7) species |
| Organism\|LargeIntestine\|Mucosa\|ColonSigmoid\|Vf (neutral lipid)-WS | 0.0483 |  | shared between all (7) species |
| Organism\|LargeIntestine\|Mucosa\|ColonSigmoid\|Vf (neutral phospholipid)-RR | 0.0124 |  | shared between all (7) species |
| Organism\|LargeIntestine\|Mucosa\|ColonSigmoid\|Vf (neutral phospholipid, plasma)-WS | 0.0182 |  | shared between all (7) species |
| Organism\|LargeIntestine\|Mucosa\|ColonSigmoid\|Vf (protein) | 0.08 |  | shared between all (7) species |
| Organism\|LargeIntestine\|Mucosa\|ColonSigmoid\|Vf (protein)-WS | 0.15 |  | shared between all (7) species |
| Organism\|LargeIntestine\|Mucosa\|ColonSigmoid\|Vf (water) | 0.8 |  | shared between all (7) species |
| Organism\|LargeIntestine\|Mucosa\|ColonSigmoid\|Vf (water)-WS | 0.78 |  | shared between all (7) species |
| Organism\|LargeIntestine\|Mucosa\|ColonTransversum\|Acidic phospholipids [mg/g] - RR | 2.41 |  | shared between all (7) species |
| Organism\|LargeIntestine\|Mucosa\|ColonTransversum\|Albumin ratio (tissue/plasma) | 0.158 |  | shared between all (7) species |
| Organism\|LargeIntestine\|Mucosa\|ColonTransversum\|Albumin ratio (tissue/plasma)-PT | 0.5 |  | shared between all (7) species |
| Organism\|LargeIntestine\|Mucosa\|ColonTransversum\|Density (tissue) | 1 | kg/dm³ | shared between all (7) species |
| Organism\|LargeIntestine\|Mucosa\|ColonTransversum\|Flow fraction via large pores | 0.05 |  | shared between all (7) species |
| Organism\|LargeIntestine\|Mucosa\|ColonTransversum\|Fraction of blood for sampling | 1 |  | shared between all (7) species |
| Organism\|LargeIntestine\|Mucosa\|ColonTransversum\|Hydraulic conductivity | 1.87E-10 | l/min/(kg*dm/min²) | shared between all (7) species |
| Organism\|LargeIntestine\|Mucosa\|ColonTransversum\|Intracellular\|pH | 7.3 |  | shared between all (7) species |
| Organism\|LargeIntestine\|Mucosa\|ColonTransversum\|Lipoprotein ratio (tissue/plasma) | 0.141 |  | shared between all (7) species |
| Organism\|LargeIntestine\|Mucosa\|ColonTransversum\|Radius (large pores) | 2.5E-07 | dm | shared between all (7) species |
| Organism\|LargeIntestine\|Mucosa\|ColonTransversum\|Radius (small pores) | 4.5E-08 | dm | shared between all (7) species |
| Organism\|LargeIntestine\|Mucosa\|ColonTransversum\|Vf (acidic phospholipids)-WS | 0.0035 |  | shared between all (7) species |
| Organism\|LargeIntestine\|Mucosa\|ColonTransversum\|Vf (extracellular water)-RR | 0.282 |  | shared between all (7) species |
| Organism\|LargeIntestine\|Mucosa\|ColonTransversum\|Vf (intracellular water)-RR | 0.456 |  | shared between all (7) species |
| Organism\|LargeIntestine\|Mucosa\|ColonTransversum\|Vf (lipid) | 0.06 |  | shared between all (7) species |
| Organism\|LargeIntestine\|Mucosa\|ColonTransversum\|Vf (neutral lipid)-RR | 0.0375 |  | shared between all (7) species |
| Organism\|LargeIntestine\|Mucosa\|ColonTransversum\|Vf (neutral lipid)-WS | 0.0483 |  | shared between all (7) species |
| Organism\|LargeIntestine\|Mucosa\|ColonTransversum\|Vf (neutral phospholipid)-RR | 0.0124 |  | shared between all (7) species |
| Organism\|LargeIntestine\|Mucosa\|ColonTransversum\|Vf (neutral phospholipid, plasma)-WS | 0.0182 |  | shared between all (7) species |
| Organism\|LargeIntestine\|Mucosa\|ColonTransversum\|Vf (protein) | 0.08 |  | shared between all (7) species |
| Organism\|LargeIntestine\|Mucosa\|ColonTransversum\|Vf (protein)-WS | 0.15 |  | shared between all (7) species |
| Organism\|LargeIntestine\|Mucosa\|ColonTransversum\|Vf (water) | 0.8 |  | shared between all (7) species |
| Organism\|LargeIntestine\|Mucosa\|ColonTransversum\|Vf (water)-WS | 0.78 |  | shared between all (7) species |
| Organism\|LargeIntestine\|Mucosa\|Rectum\|Acidic phospholipids [mg/g] - RR | 2.41 |  | shared between all (7) species |
| Organism\|LargeIntestine\|Mucosa\|Rectum\|Albumin ratio (tissue/plasma) | 0.158 |  | shared between all (7) species |
| Organism\|LargeIntestine\|Mucosa\|Rectum\|Albumin ratio (tissue/plasma)-PT | 0.5 |  | shared between all (7) species |
| Organism\|LargeIntestine\|Mucosa\|Rectum\|Density (tissue) | 1 | kg/dm³ | shared between all (7) species |
| Organism\|LargeIntestine\|Mucosa\|Rectum\|Flow fraction via large pores | 0.05 |  | shared between all (7) species |
| Organism\|LargeIntestine\|Mucosa\|Rectum\|Fraction of blood for sampling | 1 |  | shared between all (7) species |
| Organism\|LargeIntestine\|Mucosa\|Rectum\|Hydraulic conductivity | 1.87E-10 | l/min/(kg*dm/min²) | shared between all (7) species |
| Organism\|LargeIntestine\|Mucosa\|Rectum\|Intracellular\|pH | 7.3 |  | shared between all (7) species |
| Organism\|LargeIntestine\|Mucosa\|Rectum\|Lipoprotein ratio (tissue/plasma) | 0.141 |  | shared between all (7) species |
| Organism\|LargeIntestine\|Mucosa\|Rectum\|Radius (large pores) | 2.5E-07 | dm | shared between all (7) species |
| Organism\|LargeIntestine\|Mucosa\|Rectum\|Radius (small pores) | 4.5E-08 | dm | shared between all (7) species |
| Organism\|LargeIntestine\|Mucosa\|Rectum\|Vf (acidic phospholipids)-WS | 0.0035 |  | shared between all (7) species |
| Organism\|LargeIntestine\|Mucosa\|Rectum\|Vf (extracellular water)-RR | 0.282 |  | shared between all (7) species |
| Organism\|LargeIntestine\|Mucosa\|Rectum\|Vf (intracellular water)-RR | 0.456 |  | shared between all (7) species |
| Organism\|LargeIntestine\|Mucosa\|Rectum\|Vf (lipid) | 0.06 |  | shared between all (7) species |
| Organism\|LargeIntestine\|Mucosa\|Rectum\|Vf (neutral lipid)-RR | 0.0375 |  | shared between all (7) species |
| Organism\|LargeIntestine\|Mucosa\|Rectum\|Vf (neutral lipid)-WS | 0.0483 |  | shared between all (7) species |
| Organism\|LargeIntestine\|Mucosa\|Rectum\|Vf (neutral phospholipid)-RR | 0.0124 |  | shared between all (7) species |
| Organism\|LargeIntestine\|Mucosa\|Rectum\|Vf (neutral phospholipid, plasma)-WS | 0.0182 |  | shared between all (7) species |
| Organism\|LargeIntestine\|Mucosa\|Rectum\|Vf (protein) | 0.08 |  | shared between all (7) species |
| Organism\|LargeIntestine\|Mucosa\|Rectum\|Vf (protein)-WS | 0.15 |  | shared between all (7) species |
| Organism\|LargeIntestine\|Mucosa\|Rectum\|Vf (water) | 0.8 |  | shared between all (7) species |
| Organism\|LargeIntestine\|Mucosa\|Rectum\|Vf (water)-WS | 0.78 |  | shared between all (7) species |
| Organism\|LargeIntestine\|Radius (large pores) | 2.5E-07 | dm | shared between all (7) species |
| Organism\|LargeIntestine\|Radius (small pores) | 4.5E-08 | dm | shared between all (7) species |
| Organism\|LargeIntestine\|Vf (acidic phospholipids)-WS | 0.0035 |  | shared between all (7) species |
| Organism\|LargeIntestine\|Vf (extracellular water)-RR | 0.282 |  | shared between all (7) species |
| Organism\|LargeIntestine\|Vf (intracellular water)-RR | 0.456 |  | shared between all (7) species |
| Organism\|LargeIntestine\|Vf (lipid) | 0.062 |  | shared between all (7) species |
| Organism\|LargeIntestine\|Vf (neutral lipid)-RR | 0.0375 |  | shared between all (7) species |
| Organism\|LargeIntestine\|Vf (neutral lipid)-WS | 0.0483 |  | shared between all (7) species |
| Organism\|LargeIntestine\|Vf (neutral phospholipid)-RR | 0.0124 |  | shared between all (7) species |
| Organism\|LargeIntestine\|Vf (neutral phospholipid, plasma)-WS | 0.0182 |  | shared between all (7) species |
| Organism\|LargeIntestine\|Vf (protein) | 0.133 |  | shared between all (7) species |
| Organism\|LargeIntestine\|Vf (protein)-WS | 0.15 |  | shared between all (7) species |
| Organism\|LargeIntestine\|Vf (water) | 0.792 |  | shared between all (7) species |
| Organism\|LargeIntestine\|Vf (water)-WS | 0.78 |  | shared between all (7) species |
| Organism\|Liver\|Acidic phospholipids [mg/g] - RR | 4.56 |  | shared between all (7) species |
| Organism\|Liver\|Albumin ratio (tissue/plasma) | 0.086 |  | shared between all (7) species |
| Organism\|Liver\|Albumin ratio (tissue/plasma)-PT | 0.5 |  | shared between all (7) species |
| Organism\|Liver\|Allometric scale factor | 0.75 |  | shared between all (7) species |
| Organism\|Liver\|Density (tissue) | 1 | kg/dm³ | shared between all (7) species |
| Organism\|Liver\|Flow fraction via large pores | 0.8 |  | shared between all (7) species |
| Organism\|Liver\|Fraction interstitial | 0.163 |  | shared between all (7) species |
| Organism\|Liver\|Fraction of blood for sampling | 1 |  | shared between all (7) species |
| Organism\|Liver\|Fraction of periportal zone | 0.5 |  | shared between all (7) species |
| Organism\|Liver\|Fraction vascular | 0.115 |  | shared between all (7) species |
| Organism\|Liver\|Hydraulic conductivity | 3.89E-11 | l/min/(kg*dm/min²) | shared between all (7) species |
| Organism\|Liver\|Intracellular\|pH | 7.23 |  | shared between all (7) species |
| Organism\|Liver\|Is liver zonated | 0 |  | shared between all (7) species |
| Organism\|Liver\|Lipoprotein ratio (tissue/plasma) | 0.161 |  | shared between all (7) species |
| Organism\|Liver\|Radius (large pores) | 3.3E-07 | dm | shared between all (7) species |
| Organism\|Liver\|Radius (small pores) | 9E-08 | dm | shared between all (7) species |
| Organism\|Liver\|Vf (acidic phospholipids)-WS | 0.0088 |  | shared between all (7) species |
| Organism\|Liver\|Vf (extracellular water)-RR | 0.161 |  | shared between all (7) species |
| Organism\|Liver\|Vf (intracellular water)-RR | 0.481 |  | shared between all (7) species |
| Organism\|Liver\|Vf (lipid) | 0.069 |  | shared between all (7) species |
| Organism\|Liver\|Vf (neutral lipid)-RR | 0.0135 |  | shared between all (7) species |
| Organism\|Liver\|Vf (neutral lipid)-WS | 0.0232 |  | shared between all (7) species |
| Organism\|Liver\|Vf (neutral phospholipid)-RR | 0.0238 |  | shared between all (7) species |
| Organism\|Liver\|Vf (neutral phospholipid, plasma)-WS | 0.0472 |  | shared between all (7) species |
| Organism\|Liver\|Vf (protein) | 0.184 |  | shared between all (7) species |
| Organism\|Liver\|Vf (protein)-WS | 0.21 |  | shared between all (7) species |
| Organism\|Liver\|Vf (water) | 0.747 |  | shared between all (7) species |
| Organism\|Liver\|Vf (water)-WS | 0.68 |  | shared between all (7) species |
| Organism\|Lumen\|Caecum\|Microvilli factor | 1 |  | shared between all (7) species |
| Organism\|Lumen\|Caecum\|Secretion of liquid | 0 | l/min | shared between all (7) species |
| Organism\|Lumen\|ColonAscendens\|Microvilli factor | 1 |  | shared between all (7) species |
| Organism\|Lumen\|ColonDescendens\|Microvilli factor | 1 |  | shared between all (7) species |
| Organism\|Lumen\|ColonSigmoid\|Microvilli factor | 1 |  | shared between all (7) species |
| Organism\|Lumen\|ColonTransversum\|Microvilli factor | 1 |  | shared between all (7) species |
| Organism\|Lumen\|Effective surface area variability factor | 1 |  | shared between all (7) species |
| Organism\|Lumen\|Feces\|Volume | 1 | l | shared between all (7) species |
| Organism\|Lumen\|Paracellular absorption sink condition | 1 |  | shared between all (7) species |
| Organism\|Lumen\|Rectum\|Microvilli factor | 1 |  | shared between all (7) species |
| Organism\|Lumen\|Stomach\|Absorption of liquid | 0 | 1/min | shared between all (7) species |
| Organism\|Lumen\|Stomach\|GET of non-disintegrated moiety | 60 | min | shared between all (7) species |
| Organism\|Lumen\|Stomach\|GET_alpha (Weibull function) variability factor | 1 |  | shared between all (7) species |
| Organism\|Lumen\|Stomach\|GET_beta (Weibull function) variability factor | 1 |  | shared between all (7) species |
| Organism\|Lumen\|Transcellular absorption sink condition | 1 |  | shared between all (7) species |
| Organism\|Lung\|Acidic phospholipids [mg/g] - RR | 3.91 |  | shared between all (7) species |
| Organism\|Lung\|Albumin ratio (tissue/plasma) | 0.212 |  | shared between all (7) species |
| Organism\|Lung\|Albumin ratio (tissue/plasma)-PT | 0.5 |  | shared between all (7) species |
| Organism\|Lung\|Allometric scale factor | 0.75 |  | shared between all (7) species |
| Organism\|Lung\|Density (tissue) | 1 | kg/dm³ | shared between all (7) species |
| Organism\|Lung\|Flow fraction via large pores | 0.05 |  | shared between all (7) species |
| Organism\|Lung\|Fraction interstitial | 0.188 |  | shared between all (7) species |
| Organism\|Lung\|Fraction of blood for sampling | 1 |  | shared between all (7) species |
| Organism\|Lung\|Fraction vascular | 0.626 |  | shared between all (7) species |
| Organism\|Lung\|Hydraulic conductivity | 5.67E-12 | l/min/(kg*dm/min²) | shared between all (7) species |
| Organism\|Lung\|Intracellular\|pH | 6.6 |  | shared between all (7) species |
| Organism\|Lung\|Lipoprotein ratio (tissue/plasma) | 0.168 |  | shared between all (7) species |
| Organism\|Lung\|Radius (large pores) | 2.5E-07 | dm | shared between all (7) species |
| Organism\|Lung\|Radius (small pores) | 4.5E-08 | dm | shared between all (7) species |
| Organism\|Lung\|Vf (acidic phospholipids)-WS | 0.0044 |  | shared between all (7) species |
| Organism\|Lung\|Vf (extracellular water)-RR | 0.336 |  | shared between all (7) species |
| Organism\|Lung\|Vf (intracellular water)-RR | 0.238 |  | shared between all (7) species |
| Organism\|Lung\|Vf (lipid) | 0.01 |  | shared between all (7) species |
| Organism\|Lung\|Vf (neutral lipid)-RR | 0.0215 |  | shared between all (7) species |
| Organism\|Lung\|Vf (neutral lipid)-WS | 0.0204 |  | shared between all (7) species |
| Organism\|Lung\|Vf (neutral phospholipid)-RR | 0.0123 |  | shared between all (7) species |
| Organism\|Lung\|Vf (neutral phospholipid, plasma)-WS | 0.0152 |  | shared between all (7) species |
| Organism\|Lung\|Vf (protein) | 0.183 |  | shared between all (7) species |
| Organism\|Lung\|Vf (protein)-WS | 0.11 |  | shared between all (7) species |
| Organism\|Lung\|Vf (water) | 0.807 |  | shared between all (7) species |
| Organism\|Lung\|Vf (water)-WS | 0.74 |  | shared between all (7) species |
| Organism\|Muscle\|Acidic phospholipids [mg/g] - RR | 1.5 |  | shared between all (7) species |
| Organism\|Muscle\|Albumin ratio (tissue/plasma) | 0.064 |  | shared between all (7) species |
| Organism\|Muscle\|Albumin ratio (tissue/plasma)-PT | 0.5 |  | shared between all (7) species |
| Organism\|Muscle\|Allometric scale factor | 0.75 |  | shared between all (7) species |
| Organism\|Muscle\|Density (tissue) | 1 | kg/dm³ | shared between all (7) species |
| Organism\|Muscle\|Flow fraction via large pores | 0.05 |  | shared between all (7) species |
| Organism\|Muscle\|Fraction interstitial | 0.12 |  | shared between all (7) species |
| Organism\|Muscle\|Fraction of blood for sampling | 1 |  | shared between all (7) species |
| Organism\|Muscle\|Fraction vascular | 0.026 |  | shared between all (7) species |
| Organism\|Muscle\|Hydraulic conductivity | 4.17E-12 | l/min/(kg*dm/min²) | shared between all (7) species |
| Organism\|Muscle\|Intracellular\|pH | 6.81 |  | shared between all (7) species |
| Organism\|Muscle\|Lipoprotein ratio (tissue/plasma) | 0.059 |  | shared between all (7) species |
| Organism\|Muscle\|Radius (large pores) | 2.5E-07 | dm | shared between all (7) species |
| Organism\|Muscle\|Radius (small pores) | 4.5E-08 | dm | shared between all (7) species |
| Organism\|Muscle\|Vf (acidic phospholipids)-WS | 0.0009 |  | shared between all (7) species |
| Organism\|Muscle\|Vf (extracellular water)-RR | 0.118 |  | shared between all (7) species |
| Organism\|Muscle\|Vf (intracellular water)-RR | 0.608 |  | shared between all (7) species |
| Organism\|Muscle\|Vf (lipid) | 0.013 |  | shared between all (7) species |
| Organism\|Muscle\|Vf (neutral lipid)-PT | 0.01 |  | shared between all (7) species |
| Organism\|Muscle\|Vf (neutral lipid)-RR | 0.01 |  | shared between all (7) species |
| Organism\|Muscle\|Vf (neutral lipid)-WS | 0.0049 |  | shared between all (7) species |
| Organism\|Muscle\|Vf (neutral phospholipid)-RR | 0.0072 |  | shared between all (7) species |
| Organism\|Muscle\|Vf (neutral phospholipid, plasma)-WS | 0.0042 |  | shared between all (7) species |
| Organism\|Muscle\|Vf (protein) | 0.177 |  | shared between all (7) species |
| Organism\|Muscle\|Vf (protein)-WS | 0.19 |  | shared between all (7) species |
| Organism\|Muscle\|Vf (water) | 0.811 |  | shared between all (7) species |
| Organism\|Muscle\|Vf (water)-WS | 0.76 |  | shared between all (7) species |
| Organism\|Ontogeny factor (albumin) | 1 |  | shared between all (7) species |
| Organism\|Ontogeny factor (alpha1-acid glycoprotein) | 1 |  | shared between all (7) species |
| Organism\|Pancreas\|Acidic phospholipids [mg/g] - RR | 1.67 |  | shared between all (7) species |
| Organism\|Pancreas\|Albumin ratio (tissue/plasma) | 0.06 |  | shared between all (7) species |
| Organism\|Pancreas\|Albumin ratio (tissue/plasma)-PT | 0.5 |  | shared between all (7) species |
| Organism\|Pancreas\|Allometric scale factor | 0.75 |  | shared between all (7) species |
| Organism\|Pancreas\|Density (tissue) | 1 | kg/dm³ | shared between all (7) species |
| Organism\|Pancreas\|Flow fraction via large pores | 0.05 |  | shared between all (7) species |
| Organism\|Pancreas\|Fraction interstitial | 0.12 |  | shared between all (7) species |
| Organism\|Pancreas\|Fraction of blood for sampling | 1 |  | shared between all (7) species |
| Organism\|Pancreas\|Fraction vascular | 0.18 |  | shared between all (7) species |
| Organism\|Pancreas\|Hydraulic conductivity | 3.22E-11 | l/min/(kg*dm/min²) | shared between all (7) species |
| Organism\|Pancreas\|Intracellular\|pH | 7.4 |  | shared between all (7) species |
| Organism\|Pancreas\|Lipoprotein ratio (tissue/plasma) | 0.06 |  | shared between all (7) species |
| Organism\|Pancreas\|Radius (large pores) | 2.5E-07 | dm | shared between all (7) species |
| Organism\|Pancreas\|Radius (small pores) | 4.5E-08 | dm | shared between all (7) species |
| Organism\|Pancreas\|Vf (acidic phospholipids)-WS | 0.00167 |  | shared between all (7) species |
| Organism\|Pancreas\|Vf (extracellular water)-RR | 0.12 |  | shared between all (7) species |
| Organism\|Pancreas\|Vf (intracellular water)-RR | 0.521 |  | shared between all (7) species |
| Organism\|Pancreas\|Vf (lipid) | 0.08 |  | shared between all (7) species |
| Organism\|Pancreas\|Vf (neutral lipid)-PT | 0.0403 |  | shared between all (7) species |
| Organism\|Pancreas\|Vf (neutral lipid)-RR | 0.0403 |  | shared between all (7) species |
| Organism\|Pancreas\|Vf (neutral lipid)-WS | 0.0403 |  | shared between all (7) species |
| Organism\|Pancreas\|Vf (neutral phospholipid)-RR | 0.009 |  | shared between all (7) species |
| Organism\|Pancreas\|Vf (neutral phospholipid, plasma)-WS | 0.009 |  | shared between all (7) species |
| Organism\|Pancreas\|Vf (phospholipid)-PT | 0.01067 |  | shared between all (7) species |
| Organism\|Pancreas\|Vf (protein) | 0.13 |  | shared between all (7) species |
| Organism\|Pancreas\|Vf (protein)-WS | 0.13 |  | shared between all (7) species |
| Organism\|Pancreas\|Vf (water) | 0.71 |  | shared between all (7) species |
| Organism\|Pancreas\|Vf (water)-PT | 0.71 |  | shared between all (7) species |
| Organism\|Pancreas\|Vf (water)-WS | 0.71 |  | shared between all (7) species |
| Organism\|pH (blood cells) | 7.22 |  | shared between all (7) species |
| Organism\|pH (interstitial) | 7.4 |  | shared between all (7) species |
| Organism\|pH (intracellular) | 7 |  | shared between all (7) species |
| Organism\|pH (plasma) | 7.4 |  | shared between all (7) species |
| Organism\|Plasma protein scale factor | 1 |  | shared between all (7) species |
| Organism\|PortalVein\|Density (tissue) | 1 | kg/dm³ | shared between all (7) species |
| Organism\|PortalVein\|Fraction vascular | 1 |  | shared between all (7) species |
| Organism\|Protein ratio (interstial/plasma) | 0.37 |  | shared between all (7) species |
| Organism\|Rate constant for endosomal uptake (global) | 0.36 | 1/min | shared between all (7) species |
| Organism\|Rate constant for recycling from endosomal space (global) | 0.083 | 1/min | shared between all (7) species |
| Organism\|SA proportionality factor | 9500 | 1/dm | shared between all (7) species |
| Organism\|Skin\|Acidic phospholipids [mg/g] - RR | 1.32 |  | shared between all (7) species |
| Organism\|Skin\|Albumin ratio (tissue/plasma) | 0.277 |  | shared between all (7) species |
| Organism\|Skin\|Albumin ratio (tissue/plasma)-PT | 0.5 |  | shared between all (7) species |
| Organism\|Skin\|Allometric scale factor | 0.75 |  | shared between all (7) species |
| Organism\|Skin\|Density (tissue) | 1 | kg/dm³ | shared between all (7) species |
| Organism\|Skin\|Flow fraction via large pores | 0.05 |  | shared between all (7) species |
| Organism\|Skin\|Fraction interstitial | 0.302 |  | shared between all (7) species |
| Organism\|Skin\|Fraction of blood for sampling | 1 |  | shared between all (7) species |
| Organism\|Skin\|Fraction vascular | 0.019 |  | shared between all (7) species |
| Organism\|Skin\|Hydraulic conductivity | 1.67E-12 | l/min/(kg*dm/min²) | shared between all (7) species |
| Organism\|Skin\|Intracellular\|pH | 7 |  | shared between all (7) species |
| Organism\|Skin\|Lipoprotein ratio (tissue/plasma) | 0.096 |  | shared between all (7) species |
| Organism\|Skin\|Radius (large pores) | 2.5E-07 | dm | shared between all (7) species |
| Organism\|Skin\|Radius (small pores) | 4.5E-08 | dm | shared between all (7) species |
| Organism\|Skin\|Vf (acidic phospholipids)-WS | 0.0028 |  | shared between all (7) species |
| Organism\|Skin\|Vf (extracellular water)-RR | 0.382 |  | shared between all (7) species |
| Organism\|Skin\|Vf (intracellular water)-RR | 0.276 |  | shared between all (7) species |
| Organism\|Skin\|Vf (lipid) | 0.1 |  | shared between all (7) species |
| Organism\|Skin\|Vf (neutral lipid)-RR | 0.0603 |  | shared between all (7) species |
| Organism\|Skin\|Vf (neutral lipid)-WS | 0.126 |  | shared between all (7) species |
| Organism\|Skin\|Vf (neutral phospholipid)-RR | 0.0044 |  | shared between all (7) species |
| Organism\|Skin\|Vf (neutral phospholipid, plasma)-WS | 0.0112 |  | shared between all (7) species |
| Organism\|Skin\|Vf (protein) | 0.288 |  | shared between all (7) species |
| Organism\|Skin\|Vf (protein)-WS | 0.41 |  | shared between all (7) species |
| Organism\|Skin\|Vf (water) | 0.612 |  | shared between all (7) species |
| Organism\|Skin\|Vf (water)-WS | 0.47 |  | shared between all (7) species |
| Organism\|SmallIntestine\|Acidic phospholipids [mg/g] - RR | 2.41 |  | shared between all (7) species |
| Organism\|SmallIntestine\|Albumin ratio (tissue/plasma) | 0.158 |  | shared between all (7) species |
| Organism\|SmallIntestine\|Albumin ratio (tissue/plasma)-PT | 0.5 |  | shared between all (7) species |
| Organism\|SmallIntestine\|Allometric scale factor | 0.75 |  | shared between all (7) species |
| Organism\|SmallIntestine\|Density (tissue) | 1 | kg/dm³ | shared between all (7) species |
| Organism\|SmallIntestine\|Flow fraction via large pores | 0.05 |  | shared between all (7) species |
| Organism\|SmallIntestine\|Fraction interstitial | 0.094 |  | shared between all (7) species |
| Organism\|SmallIntestine\|Fraction of blood for sampling | 1 |  | shared between all (7) species |
| Organism\|SmallIntestine\|Fraction vascular | 0.024 |  | shared between all (7) species |
| Organism\|SmallIntestine\|Hydraulic conductivity | 1.54E-10 | l/min/(kg*dm/min²) | shared between all (7) species |
| Organism\|SmallIntestine\|Intracellular\|pH | 7.4 |  | shared between all (7) species |
| Organism\|SmallIntestine\|Lipoprotein ratio (tissue/plasma) | 0.141 |  | shared between all (7) species |
| Organism\|SmallIntestine\|Mucosa blood flow fraction | 0.75 |  | shared between all (7) species |
| Organism\|SmallIntestine\|Mucosa\|Duodenum\|Acidic phospholipids [mg/g] - RR | 2.41 |  | shared between all (7) species |
| Organism\|SmallIntestine\|Mucosa\|Duodenum\|Albumin ratio (tissue/plasma) | 0.158 |  | shared between all (7) species |
| Organism\|SmallIntestine\|Mucosa\|Duodenum\|Albumin ratio (tissue/plasma)-PT | 0.5 |  | shared between all (7) species |
| Organism\|SmallIntestine\|Mucosa\|Duodenum\|Density (tissue) | 1 | kg/dm³ | shared between all (7) species |
| Organism\|SmallIntestine\|Mucosa\|Duodenum\|Flow fraction via large pores | 0.05 |  | shared between all (7) species |
| Organism\|SmallIntestine\|Mucosa\|Duodenum\|Fraction of blood for sampling | 1 |  | shared between all (7) species |
| Organism\|SmallIntestine\|Mucosa\|Duodenum\|Hydraulic conductivity | 1.54E-10 | l/min/(kg*dm/min²) | shared between all (7) species |
| Organism\|SmallIntestine\|Mucosa\|Duodenum\|Intracellular\|pH | 7.3 |  | shared between all (7) species |
| Organism\|SmallIntestine\|Mucosa\|Duodenum\|Lipoprotein ratio (tissue/plasma) | 0.141 |  | shared between all (7) species |
| Organism\|SmallIntestine\|Mucosa\|Duodenum\|Radius (large pores) | 2.5E-07 | dm | shared between all (7) species |
| Organism\|SmallIntestine\|Mucosa\|Duodenum\|Radius (small pores) | 4.5E-08 | dm | shared between all (7) species |
| Organism\|SmallIntestine\|Mucosa\|Duodenum\|Vf (acidic phospholipids)-WS | 0.0035 |  | shared between all (7) species |
| Organism\|SmallIntestine\|Mucosa\|Duodenum\|Vf (extracellular water)-RR | 0.282 |  | shared between all (7) species |
| Organism\|SmallIntestine\|Mucosa\|Duodenum\|Vf (intracellular water)-RR | 0.456 |  | shared between all (7) species |
| Organism\|SmallIntestine\|Mucosa\|Duodenum\|Vf (lipid) | 0.06 |  | shared between all (7) species |
| Organism\|SmallIntestine\|Mucosa\|Duodenum\|Vf (neutral lipid)-RR | 0.0375 |  | shared between all (7) species |
| Organism\|SmallIntestine\|Mucosa\|Duodenum\|Vf (neutral lipid)-WS | 0.0483 |  | shared between all (7) species |
| Organism\|SmallIntestine\|Mucosa\|Duodenum\|Vf (neutral phospholipid)-RR | 0.0124 |  | shared between all (7) species |
| Organism\|SmallIntestine\|Mucosa\|Duodenum\|Vf (neutral phospholipid, plasma)-WS | 0.0182 |  | shared between all (7) species |
| Organism\|SmallIntestine\|Mucosa\|Duodenum\|Vf (protein) | 0.08 |  | shared between all (7) species |
| Organism\|SmallIntestine\|Mucosa\|Duodenum\|Vf (protein)-WS | 0.15 |  | shared between all (7) species |
| Organism\|SmallIntestine\|Mucosa\|Duodenum\|Vf (water) | 0.8 |  | shared between all (7) species |
| Organism\|SmallIntestine\|Mucosa\|Duodenum\|Vf (water)-WS | 0.78 |  | shared between all (7) species |
| Organism\|SmallIntestine\|Mucosa\|LowerIleum\|Acidic phospholipids [mg/g] - RR | 2.41 |  | shared between all (7) species |
| Organism\|SmallIntestine\|Mucosa\|LowerIleum\|Albumin ratio (tissue/plasma) | 0.158 |  | shared between all (7) species |
| Organism\|SmallIntestine\|Mucosa\|LowerIleum\|Albumin ratio (tissue/plasma)-PT | 0.5 |  | shared between all (7) species |
| Organism\|SmallIntestine\|Mucosa\|LowerIleum\|Density (tissue) | 1 | kg/dm³ | shared between all (7) species |
| Organism\|SmallIntestine\|Mucosa\|LowerIleum\|Flow fraction via large pores | 0.05 |  | shared between all (7) species |
| Organism\|SmallIntestine\|Mucosa\|LowerIleum\|Fraction of blood for sampling | 1 |  | shared between all (7) species |
| Organism\|SmallIntestine\|Mucosa\|LowerIleum\|Hydraulic conductivity | 1.54E-10 | l/min/(kg*dm/min²) | shared between all (7) species |
| Organism\|SmallIntestine\|Mucosa\|LowerIleum\|Intracellular\|pH | 7.3 |  | shared between all (7) species |
| Organism\|SmallIntestine\|Mucosa\|LowerIleum\|Lipoprotein ratio (tissue/plasma) | 0.141 |  | shared between all (7) species |
| Organism\|SmallIntestine\|Mucosa\|LowerIleum\|Radius (large pores) | 2.5E-07 | dm | shared between all (7) species |
| Organism\|SmallIntestine\|Mucosa\|LowerIleum\|Radius (small pores) | 4.5E-08 | dm | shared between all (7) species |
| Organism\|SmallIntestine\|Mucosa\|LowerIleum\|Vf (acidic phospholipids)-WS | 0.0035 |  | shared between all (7) species |
| Organism\|SmallIntestine\|Mucosa\|LowerIleum\|Vf (extracellular water)-RR | 0.282 |  | shared between all (7) species |
| Organism\|SmallIntestine\|Mucosa\|LowerIleum\|Vf (intracellular water)-RR | 0.456 |  | shared between all (7) species |
| Organism\|SmallIntestine\|Mucosa\|LowerIleum\|Vf (lipid) | 0.06 |  | shared between all (7) species |
| Organism\|SmallIntestine\|Mucosa\|LowerIleum\|Vf (neutral lipid)-RR | 0.0375 |  | shared between all (7) species |
| Organism\|SmallIntestine\|Mucosa\|LowerIleum\|Vf (neutral lipid)-WS | 0.0483 |  | shared between all (7) species |
| Organism\|SmallIntestine\|Mucosa\|LowerIleum\|Vf (neutral phospholipid)-RR | 0.0124 |  | shared between all (7) species |
| Organism\|SmallIntestine\|Mucosa\|LowerIleum\|Vf (neutral phospholipid, plasma)-WS | 0.0182 |  | shared between all (7) species |
| Organism\|SmallIntestine\|Mucosa\|LowerIleum\|Vf (protein) | 0.08 |  | shared between all (7) species |
| Organism\|SmallIntestine\|Mucosa\|LowerIleum\|Vf (protein)-WS | 0.15 |  | shared between all (7) species |
| Organism\|SmallIntestine\|Mucosa\|LowerIleum\|Vf (water) | 0.8 |  | shared between all (7) species |
| Organism\|SmallIntestine\|Mucosa\|LowerIleum\|Vf (water)-WS | 0.78 |  | shared between all (7) species |
| Organism\|SmallIntestine\|Mucosa\|LowerJejunum\|Acidic phospholipids [mg/g] - RR | 2.41 |  | shared between all (7) species |
| Organism\|SmallIntestine\|Mucosa\|LowerJejunum\|Albumin ratio (tissue/plasma) | 0.158 |  | shared between all (7) species |
| Organism\|SmallIntestine\|Mucosa\|LowerJejunum\|Albumin ratio (tissue/plasma)-PT | 0.5 |  | shared between all (7) species |
| Organism\|SmallIntestine\|Mucosa\|LowerJejunum\|Density (tissue) | 1 | kg/dm³ | shared between all (7) species |
| Organism\|SmallIntestine\|Mucosa\|LowerJejunum\|Flow fraction via large pores | 0.05 |  | shared between all (7) species |
| Organism\|SmallIntestine\|Mucosa\|LowerJejunum\|Fraction of blood for sampling | 1 |  | shared between all (7) species |
| Organism\|SmallIntestine\|Mucosa\|LowerJejunum\|Hydraulic conductivity | 1.54E-10 | l/min/(kg*dm/min²) | shared between all (7) species |
| Organism\|SmallIntestine\|Mucosa\|LowerJejunum\|Intracellular\|pH | 7.3 |  | shared between all (7) species |
| Organism\|SmallIntestine\|Mucosa\|LowerJejunum\|Lipoprotein ratio (tissue/plasma) | 0.141 |  | shared between all (7) species |
| Organism\|SmallIntestine\|Mucosa\|LowerJejunum\|Radius (large pores) | 2.5E-07 | dm | shared between all (7) species |
| Organism\|SmallIntestine\|Mucosa\|LowerJejunum\|Radius (small pores) | 4.5E-08 | dm | shared between all (7) species |
| Organism\|SmallIntestine\|Mucosa\|LowerJejunum\|Vf (acidic phospholipids)-WS | 0.0035 |  | shared between all (7) species |
| Organism\|SmallIntestine\|Mucosa\|LowerJejunum\|Vf (extracellular water)-RR | 0.282 |  | shared between all (7) species |
| Organism\|SmallIntestine\|Mucosa\|LowerJejunum\|Vf (intracellular water)-RR | 0.456 |  | shared between all (7) species |
| Organism\|SmallIntestine\|Mucosa\|LowerJejunum\|Vf (lipid) | 0.06 |  | shared between all (7) species |
| Organism\|SmallIntestine\|Mucosa\|LowerJejunum\|Vf (neutral lipid)-RR | 0.0375 |  | shared between all (7) species |
| Organism\|SmallIntestine\|Mucosa\|LowerJejunum\|Vf (neutral lipid)-WS | 0.0483 |  | shared between all (7) species |
| Organism\|SmallIntestine\|Mucosa\|LowerJejunum\|Vf (neutral phospholipid)-RR | 0.0124 |  | shared between all (7) species |
| Organism\|SmallIntestine\|Mucosa\|LowerJejunum\|Vf (neutral phospholipid, plasma)-WS | 0.0182 |  | shared between all (7) species |
| Organism\|SmallIntestine\|Mucosa\|LowerJejunum\|Vf (protein) | 0.08 |  | shared between all (7) species |
| Organism\|SmallIntestine\|Mucosa\|LowerJejunum\|Vf (protein)-WS | 0.15 |  | shared between all (7) species |
| Organism\|SmallIntestine\|Mucosa\|LowerJejunum\|Vf (water) | 0.8 |  | shared between all (7) species |
| Organism\|SmallIntestine\|Mucosa\|LowerJejunum\|Vf (water)-WS | 0.78 |  | shared between all (7) species |
| Organism\|SmallIntestine\|Mucosa\|UpperIleum\|Acidic phospholipids [mg/g] - RR | 2.41 |  | shared between all (7) species |
| Organism\|SmallIntestine\|Mucosa\|UpperIleum\|Albumin ratio (tissue/plasma) | 0.158 |  | shared between all (7) species |
| Organism\|SmallIntestine\|Mucosa\|UpperIleum\|Albumin ratio (tissue/plasma)-PT | 0.5 |  | shared between all (7) species |
| Organism\|SmallIntestine\|Mucosa\|UpperIleum\|Density (tissue) | 1 | kg/dm³ | shared between all (7) species |
| Organism\|SmallIntestine\|Mucosa\|UpperIleum\|Flow fraction via large pores | 0.05 |  | shared between all (7) species |
| Organism\|SmallIntestine\|Mucosa\|UpperIleum\|Fraction of blood for sampling | 1 |  | shared between all (7) species |
| Organism\|SmallIntestine\|Mucosa\|UpperIleum\|Hydraulic conductivity | 1.54E-10 | l/min/(kg*dm/min²) | shared between all (7) species |
| Organism\|SmallIntestine\|Mucosa\|UpperIleum\|Intracellular\|pH | 7.3 |  | shared between all (7) species |
| Organism\|SmallIntestine\|Mucosa\|UpperIleum\|Lipoprotein ratio (tissue/plasma) | 0.141 |  | shared between all (7) species |
| Organism\|SmallIntestine\|Mucosa\|UpperIleum\|Radius (large pores) | 2.5E-07 | dm | shared between all (7) species |
| Organism\|SmallIntestine\|Mucosa\|UpperIleum\|Radius (small pores) | 4.5E-08 | dm | shared between all (7) species |
| Organism\|SmallIntestine\|Mucosa\|UpperIleum\|Vf (acidic phospholipids)-WS | 0.0035 |  | shared between all (7) species |
| Organism\|SmallIntestine\|Mucosa\|UpperIleum\|Vf (extracellular water)-RR | 0.282 |  | shared between all (7) species |
| Organism\|SmallIntestine\|Mucosa\|UpperIleum\|Vf (intracellular water)-RR | 0.456 |  | shared between all (7) species |
| Organism\|SmallIntestine\|Mucosa\|UpperIleum\|Vf (lipid) | 0.06 |  | shared between all (7) species |
| Organism\|SmallIntestine\|Mucosa\|UpperIleum\|Vf (neutral lipid)-RR | 0.0375 |  | shared between all (7) species |
| Organism\|SmallIntestine\|Mucosa\|UpperIleum\|Vf (neutral lipid)-WS | 0.0483 |  | shared between all (7) species |
| Organism\|SmallIntestine\|Mucosa\|UpperIleum\|Vf (neutral phospholipid)-RR | 0.0124 |  | shared between all (7) species |
| Organism\|SmallIntestine\|Mucosa\|UpperIleum\|Vf (neutral phospholipid, plasma)-WS | 0.0182 |  | shared between all (7) species |
| Organism\|SmallIntestine\|Mucosa\|UpperIleum\|Vf (protein) | 0.08 |  | shared between all (7) species |
| Organism\|SmallIntestine\|Mucosa\|UpperIleum\|Vf (protein)-WS | 0.15 |  | shared between all (7) species |
| Organism\|SmallIntestine\|Mucosa\|UpperIleum\|Vf (water) | 0.8 |  | shared between all (7) species |
| Organism\|SmallIntestine\|Mucosa\|UpperIleum\|Vf (water)-WS | 0.78 |  | shared between all (7) species |
| Organism\|SmallIntestine\|Mucosa\|UpperJejunum\|Acidic phospholipids [mg/g] - RR | 2.41 |  | shared between all (7) species |
| Organism\|SmallIntestine\|Mucosa\|UpperJejunum\|Albumin ratio (tissue/plasma) | 0.158 |  | shared between all (7) species |
| Organism\|SmallIntestine\|Mucosa\|UpperJejunum\|Albumin ratio (tissue/plasma)-PT | 0.5 |  | shared between all (7) species |
| Organism\|SmallIntestine\|Mucosa\|UpperJejunum\|Density (tissue) | 1 | kg/dm³ | shared between all (7) species |
| Organism\|SmallIntestine\|Mucosa\|UpperJejunum\|Flow fraction via large pores | 0.05 |  | shared between all (7) species |
| Organism\|SmallIntestine\|Mucosa\|UpperJejunum\|Fraction of blood for sampling | 1 |  | shared between all (7) species |
| Organism\|SmallIntestine\|Mucosa\|UpperJejunum\|Hydraulic conductivity | 1.54E-10 | l/min/(kg*dm/min²) | shared between all (7) species |
| Organism\|SmallIntestine\|Mucosa\|UpperJejunum\|Intracellular\|pH | 7.3 |  | shared between all (7) species |
| Organism\|SmallIntestine\|Mucosa\|UpperJejunum\|Lipoprotein ratio (tissue/plasma) | 0.141 |  | shared between all (7) species |
| Organism\|SmallIntestine\|Mucosa\|UpperJejunum\|Radius (large pores) | 2.5E-07 | dm | shared between all (7) species |
| Organism\|SmallIntestine\|Mucosa\|UpperJejunum\|Radius (small pores) | 4.5E-08 | dm | shared between all (7) species |
| Organism\|SmallIntestine\|Mucosa\|UpperJejunum\|Vf (acidic phospholipids)-WS | 0.0035 |  | shared between all (7) species |
| Organism\|SmallIntestine\|Mucosa\|UpperJejunum\|Vf (extracellular water)-RR | 0.282 |  | shared between all (7) species |
| Organism\|SmallIntestine\|Mucosa\|UpperJejunum\|Vf (intracellular water)-RR | 0.456 |  | shared between all (7) species |
| Organism\|SmallIntestine\|Mucosa\|UpperJejunum\|Vf (lipid) | 0.06 |  | shared between all (7) species |
| Organism\|SmallIntestine\|Mucosa\|UpperJejunum\|Vf (neutral lipid)-RR | 0.0375 |  | shared between all (7) species |
| Organism\|SmallIntestine\|Mucosa\|UpperJejunum\|Vf (neutral lipid)-WS | 0.0483 |  | shared between all (7) species |
| Organism\|SmallIntestine\|Mucosa\|UpperJejunum\|Vf (neutral phospholipid)-RR | 0.0124 |  | shared between all (7) species |
| Organism\|SmallIntestine\|Mucosa\|UpperJejunum\|Vf (neutral phospholipid, plasma)-WS | 0.0182 |  | shared between all (7) species |
| Organism\|SmallIntestine\|Mucosa\|UpperJejunum\|Vf (protein) | 0.08 |  | shared between all (7) species |
| Organism\|SmallIntestine\|Mucosa\|UpperJejunum\|Vf (protein)-WS | 0.15 |  | shared between all (7) species |
| Organism\|SmallIntestine\|Mucosa\|UpperJejunum\|Vf (water) | 0.8 |  | shared between all (7) species |
| Organism\|SmallIntestine\|Mucosa\|UpperJejunum\|Vf (water)-WS | 0.78 |  | shared between all (7) species |
| Organism\|SmallIntestine\|Radius (large pores) | 2.5E-07 | dm | shared between all (7) species |
| Organism\|SmallIntestine\|Radius (small pores) | 4.5E-08 | dm | shared between all (7) species |
| Organism\|SmallIntestine\|Vf (acidic phospholipids)-WS | 0.0035 |  | shared between all (7) species |
| Organism\|SmallIntestine\|Vf (extracellular water)-RR | 0.282 |  | shared between all (7) species |
| Organism\|SmallIntestine\|Vf (intracellular water)-RR | 0.456 |  | shared between all (7) species |
| Organism\|SmallIntestine\|Vf (lipid) | 0.062 |  | shared between all (7) species |
| Organism\|SmallIntestine\|Vf (neutral lipid)-RR | 0.0375 |  | shared between all (7) species |
| Organism\|SmallIntestine\|Vf (neutral lipid)-WS | 0.0483 |  | shared between all (7) species |
| Organism\|SmallIntestine\|Vf (neutral phospholipid)-RR | 0.0124 |  | shared between all (7) species |
| Organism\|SmallIntestine\|Vf (neutral phospholipid, plasma)-WS | 0.0182 |  | shared between all (7) species |
| Organism\|SmallIntestine\|Vf (protein) | 0.133 |  | shared between all (7) species |
| Organism\|SmallIntestine\|Vf (protein)-WS | 0.15 |  | shared between all (7) species |
| Organism\|SmallIntestine\|Vf (water) | 0.792 |  | shared between all (7) species |
| Organism\|SmallIntestine\|Vf (water)-WS | 0.78 |  | shared between all (7) species |
| Organism\|Spleen\|Acidic phospholipids [mg/g] - RR | 3.18 |  | shared between all (7) species |
| Organism\|Spleen\|Albumin ratio (tissue/plasma) | 0.097 |  | shared between all (7) species |
| Organism\|Spleen\|Albumin ratio (tissue/plasma)-PT | 0.5 |  | shared between all (7) species |
| Organism\|Spleen\|Allometric scale factor | 0.75 |  | shared between all (7) species |
| Organism\|Spleen\|Density (tissue) | 1 | kg/dm³ | shared between all (7) species |
| Organism\|Spleen\|Flow fraction via large pores | 0.8 |  | shared between all (7) species |
| Organism\|Spleen\|Fraction interstitial | 0.15 |  | shared between all (7) species |
| Organism\|Spleen\|Fraction of blood for sampling | 1 |  | shared between all (7) species |
| Organism\|Spleen\|Fraction vascular | 0.282 |  | shared between all (7) species |
| Organism\|Spleen\|Hydraulic conductivity | 3.89E-11 | l/min/(kg*dm/min²) | shared between all (7) species |
| Organism\|Spleen\|Intracellular\|pH | 7 |  | shared between all (7) species |
| Organism\|Spleen\|Lipoprotein ratio (tissue/plasma) | 0.207 |  | shared between all (7) species |
| Organism\|Spleen\|Radius (large pores) | 3.3E-07 | dm | shared between all (7) species |
| Organism\|Spleen\|Radius (small pores) | 9E-08 | dm | shared between all (7) species |
| Organism\|Spleen\|Vf (acidic phospholipids)-WS | 0.003 |  | shared between all (7) species |
| Organism\|Spleen\|Vf (extracellular water)-RR | 0.207 |  | shared between all (7) species |
| Organism\|Spleen\|Vf (intracellular water)-RR | 0.355 |  | shared between all (7) species |
| Organism\|Spleen\|Vf (lipid) | 0.016 |  | shared between all (7) species |
| Organism\|Spleen\|Vf (neutral lipid)-RR | 0.0071 |  | shared between all (7) species |
| Organism\|Spleen\|Vf (neutral lipid)-WS | 0.006 |  | shared between all (7) species |
| Organism\|Spleen\|Vf (neutral phospholipid)-RR | 0.0107 |  | shared between all (7) species |
| Organism\|Spleen\|Vf (neutral phospholipid, plasma)-WS | 0.0108 |  | shared between all (7) species |
| Organism\|Spleen\|Vf (protein) | 0.194 |  | shared between all (7) species |
| Organism\|Spleen\|Vf (protein)-WS | 0.23 |  | shared between all (7) species |
| Organism\|Spleen\|Vf (water) | 0.778 |  | shared between all (7) species |
| Organism\|Spleen\|Vf (water)-WS | 0.75 |  | shared between all (7) species |
| Organism\|Stomach\|Acidic phospholipids [mg/g] - RR | 2.41 |  | shared between all (7) species |
| Organism\|Stomach\|Albumin ratio (tissue/plasma) | 0.158 |  | shared between all (7) species |
| Organism\|Stomach\|Albumin ratio (tissue/plasma)-PT | 0.5 |  | shared between all (7) species |
| Organism\|Stomach\|Allometric scale factor | 0.75 |  | shared between all (7) species |
| Organism\|Stomach\|Density (tissue) | 1 | kg/dm³ | shared between all (7) species |
| Organism\|Stomach\|Flow fraction via large pores | 0.05 |  | shared between all (7) species |
| Organism\|Stomach\|Fraction interstitial | 0.1 |  | shared between all (7) species |
| Organism\|Stomach\|Fraction of blood for sampling | 1 |  | shared between all (7) species |
| Organism\|Stomach\|Fraction vascular | 0.032 |  | shared between all (7) species |
| Organism\|Stomach\|Hydraulic conductivity | 3.97E-11 | l/min/(kg*dm/min²) | shared between all (7) species |
| Organism\|Stomach\|Intracellular\|pH | 7.4 |  | shared between all (7) species |
| Organism\|Stomach\|Lipoprotein ratio (tissue/plasma) | 0.141 |  | shared between all (7) species |
| Organism\|Stomach\|Radius (large pores) | 2.5E-07 | dm | shared between all (7) species |
| Organism\|Stomach\|Radius (small pores) | 4.5E-08 | dm | shared between all (7) species |
| Organism\|Stomach\|Vf (acidic phospholipids)-WS | 0.0035 |  | shared between all (7) species |
| Organism\|Stomach\|Vf (extracellular water)-RR | 0.282 |  | shared between all (7) species |
| Organism\|Stomach\|Vf (intracellular water)-RR | 0.456 |  | shared between all (7) species |
| Organism\|Stomach\|Vf (lipid) | 0.062 |  | shared between all (7) species |
| Organism\|Stomach\|Vf (neutral lipid)-RR | 0.0375 |  | shared between all (7) species |
| Organism\|Stomach\|Vf (neutral lipid)-WS | 0.0483 |  | shared between all (7) species |
| Organism\|Stomach\|Vf (neutral phospholipid)-RR | 0.0124 |  | shared between all (7) species |
| Organism\|Stomach\|Vf (neutral phospholipid, plasma)-WS | 0.0182 |  | shared between all (7) species |
| Organism\|Stomach\|Vf (protein) | 0.133 |  | shared between all (7) species |
| Organism\|Stomach\|Vf (protein)-WS | 0.15 |  | shared between all (7) species |
| Organism\|Stomach\|Vf (water) | 0.792 |  | shared between all (7) species |
| Organism\|Stomach\|Vf (water)-WS | 0.78 |  | shared between all (7) species |
| Organism\|Surface area scaling exponent | 1 |  | shared between all (7) species |
| Organism\|Thickness (endothelium) | 0.000003 | dm | shared between all (7) species |
| Organism\|VenousBlood\|Density (tissue) | 1 | kg/dm³ | shared between all (7) species |
| Organism\|VenousBlood\|Fraction vascular | 1 |  | shared between all (7) species |
| Organism\|Vf (acidic phospholipids, blood cells)-WS | 0.001 |  | shared between all (7) species |
| Organism\|Vf (intracellular water, blood cells)-RR | 0.6 |  | shared between all (7) species |
| Organism\|Vf (lipid, blood cells) | 0.005 |  | shared between all (7) species |
| Organism\|Vf (lipid, plasma) | 0.007 |  | shared between all (7) species |
| Organism\|Vf (neutral lipid, blood cells)-RR | 0.0017 |  | shared between all (7) species |
| Organism\|Vf (neutral lipid, blood cells)-WS | 0.003 |  | shared between all (7) species |
| Organism\|Vf (neutral lipid, plasma)-RR | 0.0023 |  | shared between all (7) species |
| Organism\|Vf (neutral phospholipid, blood cells)-RR | 0.0029 |  | shared between all (7) species |
| Organism\|Vf (neutral phospholipid, blood cells)-WS | 0.0059 |  | shared between all (7) species |
| Organism\|Vf (neutral phospholipid, plasma)-RR | 0.0013 |  | shared between all (7) species |
| Organism\|Vf (protein,blood cells) | 0.325 |  | shared between all (7) species |
| Organism\|Vf (protein,plasma) | 0.067 |  | shared between all (7) species |
| Organism\|Vf (water,blood cells) | 0.625 |  | shared between all (7) species |
| Organism\|Vf (water,interstitial) | 0.935 |  | shared between all (7) species |
| Organism\|Vf (water,plasma) | 0.926 |  | shared between all (7) species |
| Organism\|Bone\|Vf (neutral lipid)-PT | 0.0174 |  | shared with several (>3) species |
| Organism\|Bone\|Vf (phospholipid)-PT | 0.00227 |  | shared with several (>3) species |
| Organism\|Bone\|Vf (water)-PT | 0.465 |  | shared with several (>3) species |
| Organism\|Brain\|Vf (neutral lipid)-PT | 0.0391 |  | shared with several (>3) species |
| Organism\|Brain\|Vf (phospholipid)-PT | 0.0019 |  | shared with several (>3) species |
| Organism\|Brain\|Vf (water)-PT | 0.808 |  | shared with several (>3) species |
| Organism\|Fat\|Vf (intracellular water)-RR | 0.009 |  | shared with several (>3) species |
| Organism\|Fat\|Vf (water)-PT | 0.15 |  | shared with several (>3) species |
| Organism\|Gallbladder\|Gallbladder ejection fraction | 0.65 |  | shared with several (>3) species |
| Organism\|Gallbladder\|Gallbladder ejection half-time | 19.7 | min | shared with several (>3) species |
| Organism\|Gallbladder\|Volume | 1 | l | shared with several (>3) species |
| Organism\|Heart\|Vf (neutral lipid)-PT | 0.0135 |  | shared with several (>3) species |
| Organism\|Heart\|Vf (phospholipid)-PT | 0.01285 |  | shared with several (>3) species |
| Organism\|Heart\|Vf (water)-PT | 0.731 |  | shared with several (>3) species |
| Organism\|Kidney\|Vf (neutral lipid)-PT | 0.0121 |  | shared with several (>3) species |
| Organism\|Kidney\|Vf (phospholipid)-PT | 0.02903 |  | shared with several (>3) species |
| Organism\|Kidney\|Vf (water)-PT | 0.774 |  | shared with several (>3) species |
| Organism\|LargeIntestine\|Mucosa\|Caecum\|Fraction vascular | 0.09 |  | shared with several (>3) species |
| Organism\|LargeIntestine\|Mucosa\|Caecum\|Vf (neutral lipid)-PT | 0.0375 |  | shared with several (>3) species |
| Organism\|LargeIntestine\|Mucosa\|Caecum\|Vf (phospholipid)-PT | 0.01481 |  | shared with several (>3) species |
| Organism\|LargeIntestine\|Mucosa\|Caecum\|Vf (water)-PT | 0.792 |  | shared with several (>3) species |
| Organism\|LargeIntestine\|Mucosa\|ColonAscendens\|Fraction vascular | 0.08 |  | shared with several (>3) species |
| Organism\|LargeIntestine\|Mucosa\|ColonAscendens\|Vf (neutral lipid)-PT | 0.0375 |  | shared with several (>3) species |
| Organism\|LargeIntestine\|Mucosa\|ColonAscendens\|Vf (phospholipid)-PT | 0.01481 |  | shared with several (>3) species |
| Organism\|LargeIntestine\|Mucosa\|ColonAscendens\|Vf (water)-PT | 0.792 |  | shared with several (>3) species |
| Organism\|LargeIntestine\|Mucosa\|ColonDescendens\|Fraction vascular | 0.09 |  | shared with several (>3) species |
| Organism\|LargeIntestine\|Mucosa\|ColonDescendens\|Vf (neutral lipid)-PT | 0.0375 |  | shared with several (>3) species |
| Organism\|LargeIntestine\|Mucosa\|ColonDescendens\|Vf (phospholipid)-PT | 0.01481 |  | shared with several (>3) species |
| Organism\|LargeIntestine\|Mucosa\|ColonDescendens\|Vf (water)-PT | 0.792 |  | shared with several (>3) species |
| Organism\|LargeIntestine\|Mucosa\|ColonSigmoid\|Fraction vascular | 0.09 |  | shared with several (>3) species |
| Organism\|LargeIntestine\|Mucosa\|ColonSigmoid\|Vf (neutral lipid)-PT | 0.0375 |  | shared with several (>3) species |
| Organism\|LargeIntestine\|Mucosa\|ColonSigmoid\|Vf (phospholipid)-PT | 0.01481 |  | shared with several (>3) species |
| Organism\|LargeIntestine\|Mucosa\|ColonSigmoid\|Vf (water)-PT | 0.792 |  | shared with several (>3) species |
| Organism\|LargeIntestine\|Mucosa\|ColonTransversum\|Vf (neutral lipid)-PT | 0.0375 |  | shared with several (>3) species |
| Organism\|LargeIntestine\|Mucosa\|ColonTransversum\|Vf (phospholipid)-PT | 0.01481 |  | shared with several (>3) species |
| Organism\|LargeIntestine\|Mucosa\|ColonTransversum\|Vf (water)-PT | 0.792 |  | shared with several (>3) species |
| Organism\|LargeIntestine\|Mucosa\|Rectum\|Fraction vascular | 0.09 |  | shared with several (>3) species |
| Organism\|LargeIntestine\|Mucosa\|Rectum\|Vf (neutral lipid)-PT | 0.0375 |  | shared with several (>3) species |
| Organism\|LargeIntestine\|Mucosa\|Rectum\|Vf (phospholipid)-PT | 0.01481 |  | shared with several (>3) species |
| Organism\|LargeIntestine\|Mucosa\|Rectum\|Vf (water)-PT | 0.792 |  | shared with several (>3) species |
| Organism\|LargeIntestine\|Vf (neutral lipid)-PT | 0.0375 |  | shared with several (>3) species |
| Organism\|LargeIntestine\|Vf (phospholipid)-PT | 0.01481 |  | shared with several (>3) species |
| Organism\|LargeIntestine\|Vf (water)-PT | 0.792 |  | shared with several (>3) species |
| Organism\|Liver\|EHC continuous fraction | 0 |  | shared with several (>3) species |
| Organism\|Liver\|Vf (neutral lipid)-PT | 0.0135 |  | shared with several (>3) species |
| Organism\|Liver\|Vf (phospholipid)-PT | 0.02836 |  | shared with several (>3) species |
| Organism\|Liver\|Vf (water)-PT | 0.747 |  | shared with several (>3) species |
| Organism\|Lumen\|Duodenum\|Fractional steady state fill level | 0.06 |  | shared with several (>3) species |
| Organism\|Lung\|Vf (neutral lipid)-PT | 0.0215 |  | shared with several (>3) species |
| Organism\|Lung\|Vf (phospholipid)-PT | 0.01621 |  | shared with several (>3) species |
| Organism\|Lung\|Vf (water)-PT | 0.807 |  | shared with several (>3) species |
| Organism\|Muscle\|Peripheral blood flow fraction | 0.3 |  | shared with several (>3) species |
| Organism\|Muscle\|Vf (phospholipid)-PT | 0.0087 |  | shared with several (>3) species |
| Organism\|Muscle\|Vf (water)-PT | 0.811 |  | shared with several (>3) species |
| Organism\|Skin\|Peripheral blood flow fraction | 0.7 |  | shared with several (>3) species |
| Organism\|Skin\|Vf (neutral lipid)-PT | 0.0603 |  | shared with several (>3) species |
| Organism\|Skin\|Vf (phospholipid)-PT | 0.00572 |  | shared with several (>3) species |
| Organism\|Skin\|Vf (water)-PT | 0.612 |  | shared with several (>3) species |
| Organism\|SmallIntestine\|Mucosa\|Duodenum\|Fraction vascular | 0.09 |  | shared with several (>3) species |
| Organism\|SmallIntestine\|Mucosa\|Duodenum\|Vf (neutral lipid)-PT | 0.0375 |  | shared with several (>3) species |
| Organism\|SmallIntestine\|Mucosa\|Duodenum\|Vf (phospholipid)-PT | 0.01481 |  | shared with several (>3) species |
| Organism\|SmallIntestine\|Mucosa\|Duodenum\|Vf (water)-PT | 0.792 |  | shared with several (>3) species |
| Organism\|SmallIntestine\|Mucosa\|LowerIleum\|Fraction vascular | 0.09 |  | shared with several (>3) species |
| Organism\|SmallIntestine\|Mucosa\|LowerIleum\|Vf (neutral lipid)-PT | 0.0375 |  | shared with several (>3) species |
| Organism\|SmallIntestine\|Mucosa\|LowerIleum\|Vf (phospholipid)-PT | 0.01481 |  | shared with several (>3) species |
| Organism\|SmallIntestine\|Mucosa\|LowerIleum\|Vf (water)-PT | 0.792 |  | shared with several (>3) species |
| Organism\|SmallIntestine\|Mucosa\|LowerJejunum\|Fraction vascular | 0.09 |  | shared with several (>3) species |
| Organism\|SmallIntestine\|Mucosa\|LowerJejunum\|Vf (neutral lipid)-PT | 0.0375 |  | shared with several (>3) species |
| Organism\|SmallIntestine\|Mucosa\|LowerJejunum\|Vf (phospholipid)-PT | 0.01481 |  | shared with several (>3) species |
| Organism\|SmallIntestine\|Mucosa\|LowerJejunum\|Vf (water)-PT | 0.792 |  | shared with several (>3) species |
| Organism\|SmallIntestine\|Mucosa\|UpperIleum\|Fraction vascular | 0.09 |  | shared with several (>3) species |
| Organism\|SmallIntestine\|Mucosa\|UpperIleum\|Vf (neutral lipid)-PT | 0.0375 |  | shared with several (>3) species |
| Organism\|SmallIntestine\|Mucosa\|UpperIleum\|Vf (phospholipid)-PT | 0.01481 |  | shared with several (>3) species |
| Organism\|SmallIntestine\|Mucosa\|UpperIleum\|Vf (water)-PT | 0.792 |  | shared with several (>3) species |
| Organism\|SmallIntestine\|Mucosa\|UpperJejunum\|Fraction vascular | 0.09 |  | shared with several (>3) species |
| Organism\|SmallIntestine\|Mucosa\|UpperJejunum\|Vf (neutral lipid)-PT | 0.0375 |  | shared with several (>3) species |
| Organism\|SmallIntestine\|Mucosa\|UpperJejunum\|Vf (phospholipid)-PT | 0.01481 |  | shared with several (>3) species |
| Organism\|SmallIntestine\|Mucosa\|UpperJejunum\|Vf (water)-PT | 0.792 |  | shared with several (>3) species |
| Organism\|SmallIntestine\|Vf (neutral lipid)-PT | 0.0375 |  | shared with several (>3) species |
| Organism\|SmallIntestine\|Vf (phospholipid)-PT | 0.01481 |  | shared with several (>3) species |
| Organism\|SmallIntestine\|Vf (water)-PT | 0.792 |  | shared with several (>3) species |
| Organism\|Spleen\|Vf (neutral lipid)-PT | 0.0071 |  | shared with several (>3) species |
| Organism\|Spleen\|Vf (phospholipid)-PT | 0.01388 |  | shared with several (>3) species |
| Organism\|Spleen\|Vf (water)-PT | 0.778 |  | shared with several (>3) species |
| Organism\|Stomach\|Vf (neutral lipid)-PT | 0.0375 |  | shared with several (>3) species |
| Organism\|Stomach\|Vf (phospholipid)-PT | 0.01481 |  | shared with several (>3) species |
| Organism\|Stomach\|Vf (water)-PT | 0.792 |  | shared with several (>3) species |
| Organism\|Vf (neutral lipid, plasma)-PT | 0.0023 |  | shared with several (>3) species |
| Organism\|Vf (phospholipid, plasma)-PT | 0.0013 |  | shared with several (>3) species |
| Organism\|Vf (water,plasma)-PT | 0.926 |  | shared with several (>3) species |
| Organism\|LargeIntestine\|Mucosa\|Caecum\|Fraction interstitial | 0.42 |  | transfered from mouse to rabbit |
| Organism\|LargeIntestine\|Mucosa\|Caecum\|Fraction of regional blood flow rate | 0.37919 |  | transfered from mouse to rabbit |
| Organism\|LargeIntestine\|Mucosa\|ColonAscendens\|Fraction interstitial | 0.68 |  | transfered from mouse to rabbit |
| Organism\|LargeIntestine\|Mucosa\|ColonAscendens\|Fraction of regional blood flow rate | 0.1552 |  | transfered from mouse to rabbit |
| Organism\|LargeIntestine\|Mucosa\|ColonDescendens\|Fraction interstitial | 0.67 |  | transfered from mouse to rabbit |
| Organism\|LargeIntestine\|Mucosa\|ColonDescendens\|Fraction of regional blood flow rate | 0.10347 |  | transfered from mouse to rabbit |
| Organism\|LargeIntestine\|Mucosa\|ColonSigmoid\|Fraction interstitial | 0.67 |  | transfered from mouse to rabbit |
| Organism\|LargeIntestine\|Mucosa\|ColonSigmoid\|Fraction of regional blood flow rate | 0.10347 |  | transfered from mouse to rabbit |
| Organism\|LargeIntestine\|Mucosa\|ColonTransversum\|Fraction interstitial | 0.68 |  | transfered from mouse to rabbit |
| Organism\|LargeIntestine\|Mucosa\|ColonTransversum\|Fraction of regional blood flow rate | 0.1552 |  | transfered from mouse to rabbit |
| Organism\|LargeIntestine\|Mucosa\|ColonTransversum\|Fraction vascular | 0.08 |  | transfered from mouse to rabbit |
| Organism\|LargeIntestine\|Mucosa\|Rectum\|Fraction interstitial | 0.76 |  | transfered from mouse to rabbit |
| Organism\|LargeIntestine\|Mucosa\|Rectum\|Fraction of regional blood flow rate | 0.10347 |  | transfered from mouse to rabbit |
| Organism\|Liver\|Number of cells/g tissue | 135000 | x10^6/kg | transfered from mouse to rabbit |
| Organism\|Lumen\|Caecum\|Default thickness of gut wall | 0.001512 | dm | transfered from mouse to rabbit |
| Organism\|Lumen\|Caecum\|Fractional steady state fill level | 0.14572 |  | transfered from mouse to rabbit |
| Organism\|Lumen\|ColonAscendens\|Default thickness of gut wall | 0.003748 | dm | transfered from mouse to rabbit |
| Organism\|Lumen\|ColonAscendens\|Fractional steady state fill level | 0.14572 |  | transfered from mouse to rabbit |
| Organism\|Lumen\|ColonDescendens\|Default thickness of gut wall | 0.003748 | dm | transfered from mouse to rabbit |
| Organism\|Lumen\|ColonDescendens\|Fractional steady state fill level | 0.14572 |  | transfered from mouse to rabbit |
| Organism\|Lumen\|ColonSigmoid\|Default thickness of gut wall | 0.003748 | dm | transfered from mouse to rabbit |
| Organism\|Lumen\|ColonSigmoid\|Fractional steady state fill level | 0.14572 |  | transfered from mouse to rabbit |
| Organism\|Lumen\|ColonTransversum\|Default thickness of gut wall | 0.003748 | dm | transfered from mouse to rabbit |
| Organism\|Lumen\|ColonTransversum\|Fractional steady state fill level | 0.14572 |  | transfered from mouse to rabbit |
| Organism\|Lumen\|Duodenum\|Default thickness of gut wall | 0.004852 | dm | transfered from mouse to rabbit |
| Organism\|Lumen\|LowerIleum\|Default thickness of gut wall | 0.002487 | dm | transfered from mouse to rabbit |
| Organism\|Lumen\|LowerIleum\|Fractional steady state fill level | 0.12371 |  | transfered from mouse to rabbit |
| Organism\|Lumen\|LowerJejunum\|Default thickness of gut wall | 0.003545 | dm | transfered from mouse to rabbit |
| Organism\|Lumen\|LowerJejunum\|Fractional steady state fill level | 0.12371 |  | transfered from mouse to rabbit |
| Organism\|Lumen\|Rectum\|Default thickness of gut wall | 0.003748 | dm | transfered from mouse to rabbit |
| Organism\|Lumen\|Rectum\|Fractional steady state fill level | 0.14572 |  | transfered from mouse to rabbit |
| Organism\|Lumen\|Stomach\|Default thickness of gut wall | 0.004377 | dm | transfered from mouse to rabbit |
| Organism\|Lumen\|Stomach\|Fractional steady state fill level | 0.66314 |  | transfered from mouse to rabbit |
| Organism\|Lumen\|Stomach\|Inverse rate of inflow of liquid into stomach | 20 | min | transfered from mouse to rabbit |
| Organism\|Lumen\|UpperIleum\|Default thickness of gut wall | 0.002487 | dm | transfered from mouse to rabbit |
| Organism\|Lumen\|UpperIleum\|Fractional steady state fill level | 0.12371 |  | transfered from mouse to rabbit |
| Organism\|Lumen\|UpperJejunum\|Default thickness of gut wall | 0.003545 | dm | transfered from mouse to rabbit |
| Organism\|Lumen\|UpperJejunum\|Fractional steady state fill level | 0.12371 |  | transfered from mouse to rabbit |
| Organism\|SmallIntestine\|Mucosa\|Duodenum\|Fraction interstitial | 0.15 |  | transfered from mouse to rabbit |
| Organism\|SmallIntestine\|Mucosa\|Duodenum\|Fraction mucosa | 0.7 |  | transfered from mouse to rabbit |
| Organism\|SmallIntestine\|Mucosa\|Duodenum\|Fraction of regional blood flow rate | 0.16943 |  | transfered from mouse to rabbit |
| Organism\|SmallIntestine\|Mucosa\|LowerIleum\|Fraction interstitial | 0.15 |  | transfered from mouse to rabbit |
| Organism\|SmallIntestine\|Mucosa\|LowerIleum\|Fraction mucosa | 0.7 |  | transfered from mouse to rabbit |
| Organism\|SmallIntestine\|Mucosa\|LowerIleum\|Fraction of regional blood flow rate | 0.044366 |  | transfered from mouse to rabbit |
| Organism\|SmallIntestine\|Mucosa\|LowerJejunum\|Fraction interstitial | 0.15 |  | transfered from mouse to rabbit |
| Organism\|SmallIntestine\|Mucosa\|LowerJejunum\|Fraction mucosa | 0.7 |  | transfered from mouse to rabbit |
| Organism\|SmallIntestine\|Mucosa\|LowerJejunum\|Fraction of regional blood flow rate | 0.35599 |  | transfered from mouse to rabbit |
| Organism\|SmallIntestine\|Mucosa\|UpperIleum\|Fraction interstitial | 0.15 |  | transfered from mouse to rabbit |
| Organism\|SmallIntestine\|Mucosa\|UpperIleum\|Fraction mucosa | 0.7 |  | transfered from mouse to rabbit |
| Organism\|SmallIntestine\|Mucosa\|UpperIleum\|Fraction of regional blood flow rate | 0.044895 |  | transfered from mouse to rabbit |
| Organism\|SmallIntestine\|Mucosa\|UpperJejunum\|Fraction interstitial | 0.15 |  | transfered from mouse to rabbit |
| Organism\|SmallIntestine\|Mucosa\|UpperJejunum\|Fraction mucosa | 0.7 |  | transfered from mouse to rabbit |
| Organism\|SmallIntestine\|Mucosa\|UpperJejunum\|Fraction of regional blood flow rate | 0.38532 |  | transfered from mouse to rabbit |

# References

1. Davies B, Morris T. Physiological parameters in laboratory animals and humans. Pharmaceutical research. 1993;10(7):1093-5.

2. Thorburn GD, Casey B, Molyneux GS. Distribution of blood flow within the skin of the rabbit with particular reference to hair growth. Circulation research. 1966;18:650-9.

3. Skelton H. The storage of water by various tissues of the body. Arch Intern Med (Chic). 1927;40(2):140-52.

4. Crile G, Quiring DP. A record of the body weight and certain organ and gland volumes of 3690 animals. The Ohio Journal of Science. 1940;XL(5):219-59.

5. Lebas F, Coudert P, de Rochambeau H, Thébault R. The Rabbit: Husbandry, health and production. FAO Animal Production and Health Series No 21. Rome: Food and agriculture organization of the United Nations; 1997.

6. Debray L, Le Huerou-Luron I, Gidenne T, Fortun-Lamothe L. Digestive tract development in rabbit according to the dietary energetic source: correlation between whole tract digestion, pancreatic and intestinal enzymatic activities. Comp Biochem Physiol A Mol Integr Physiol. 2003;135(3):443-55. PubMed PMID: 12829052.

7. Brown WH, Pearce L, Van Allen CM. Organ weights of normal rabbits. J Exp Med. 1925;42(1):69-82.

8. Houdebine L-M. Rabbit Biotechnology. Fan J, SpringerLink, editors. Dordrecht: Springer Netherlands; 2009.

9. Davies B, Morris T. Physiological parameters in laboratory animals and humans. Pharm Res. 1993;10(7):1093-5. PubMed PMID: 8378254.

10. Davis T, Holloway, I, Pooley J. The effect of anaesthesia on the bone blood flow of the rabbit. J Orthopaedic Research. 1990;8:479-84.

11. Bill A. Effects of indomethacin on regional blood flow in conscious rabbits - a microsphere study. Acta physiol scand. 1979;105:437-42.

12. Cianci T, Zoccoli G, Lenzi P, Franzini C. Regional splanchnic blood flow during sleep in rabbits. Pflügers Arch. 1990;415:594-7.

13. Lifson N, Kramlinger KG, Mayrand RR, Lender EJ. Blood flow to the rabbit pancreas with special reference to the islets of Langerhans. Gastroenterology. 1980;79:466-73.

14. Sweeney LM, Kirman CR, Gannon SA, Thrall KD, Gargas ML, Kinzell JH. Development of physiologically based pharmacokinetic (PBPK) model for methyl iodide in rats, rabbits and humans. Inhalation Toxicology. 2009;21(6):552-82.

15. Ünlüer S, Ercan M, Akdas A. Testicular blood flow in experimental torsion and epididymo-orchitis measured by 13Xe clearance techniques in rabbits. Urological research. 1984;12:183-6.

16. Kararli TT. Comparison of the gastrointestinal anatomy, physiology, and biochemistry of humans and commonly used laboratory animals. Biopharm Drug Dispos. 1995;16(5):351-80. PubMed PMID: 8527686.

17. Rozman K. Disposition of xenobiotics: species differences. Toxicol Pathol. 1988;16(2):123-9. doi: 10.1177/019262338801600204. PubMed PMID: 3055221.

18. Hatton GB, Yadav V, Basit AW, Merchant HA. Animal Farm: Considerations in animal gastrointestinal physiology and relevance to drug delivery in humans. J Pharmaceutical Sciences. 2015;104:2747-76.

19. Schulze-Delrieu K, Wall JP. Determinants of flow across isolated gastroduodenal junctions of cats and rabbits. Am J Physiol. 1983;245(2):G257-64. PubMed PMID: 6881349.

20. de Zwart L, Rompelberg C, Sips A, Welink J, van Engelen J. Anatomical and physiological differences between various species used in studies on the pharmacokinetics and toxicology of xenobiotics (RIVM report 623860 010). Bilthoven, NL: Rijksinstituut voor volksgezondheid en milieu, 1999.

21. Sibly R, Monk K, Johnson I, Trout R. Seasonal variation in gut morphology in wild rabbits (Oryctolagus cuniculus). J Zool Lond. 1990;221:605-19.

22. Rees Davies R, Rees Davies JA. Rabbit gastrointestinal physiology. Vet Clin Exot Anim. 2003;6:139-53.

23. DOROTEA SB, Banzato T, Bellini L, Contiero B, Zotti A. RADIOGRAPHIC ANATOMY OF DWARF RABBIT ABDOMEN WITH NORMAL MEASUREMENTS. Bulgarian Journal of Veterinary Medicine. 2016;19(2):96-107.

24. Sohn J, Couto MA. Chapter 8 - Anatomy, Physiology, and Behavior. The Laboratory Rabbit, Guinea Pig, Hamster, and Other Rodents. Boston: Academic Press; 2012. p. 195-215.

25. Snipes RL. Intestinal Absorptive Surface in Mammals of Different Sizes. Berlin: Springer; 1997.

26. Helander HF, Fandriks L. Surface area of the digestive tract - revisited. Scandinavian journal of gastroenterology. 2014;49(6):681-9. doi: 10.3109/00365521.2014.898326. PubMed PMID: 24694282.

27. Thomson A, Keelan M, Tavernini M, Luethe D, Lam T. Development of active and passive transport of bile acids in rabbit intestine. Mechanisms of ageing and development. 1987;38:277-86.

28. Westergaard H, Dietschy JM. Delineation of the dimensions and permeability characteristics of the two major diffusion barriers to passive mucosal uptake in the rabbit intestine. The Journal of Clinical Investigation. 1974;54:718-32.

29. Michigoshi Y, Yamagishi N, Satoh H, Kato M, Furuhama K. Using a single blood sample and inulin to estimate glomerular filtration rate in rabbits. Journal of the American Association for Laboratory Animal Science : JAALAS. 2011;50(5):702-7. PubMed PMID: 22330718; PubMed Central PMCID: PMC3189675.

30. Grant DM, Campbell ME, Tang BK, Kalow W. Biotransformation of caffeine by microsomes from human liver. Kinetics and inhibition studies. Biochemical pharmacology. 1987;36(8):1251-60. PubMed PMID: 3593412.

31. Thiel C, Schneckener S, Krauss M, Ghallab A, Hofmann U, Kanacher T, et al. A systematic evaluation of the use of physiologically based pharmacokinetic modeling for cross-species extrapolation. J Pharm Sci. 2015;104(1):191-206. doi: 10.1002/jps.24214. PubMed PMID: 25393841.

32. Beach CA, Mays DC, Sterman BM, Gerber N. Metabolism, distribution, seminal excretion and pharmacokinetics of caffeine in the rabbit. The Journal of pharmacology and experimental therapeutics. 1985;233(1):18-23. PubMed PMID: 3981454.

33. Lamp KC, Bailey EM, Rybak MJ. Ofloxacin clinical pharmacokinetics. Clin Pharmacokinet. 1992;22(1):32-46. doi: 10.2165/00003088-199222010-00004. PubMed PMID: 1559306.

34. Marangos MN, Zhu Z, Nicolau DP, Klepser ME, Nightingale CH. Disposition of ofloxacin in female New Zealand white rabbits. Journal of veterinary pharmacology and therapeutics. 1997;20(1):17-20. PubMed PMID: 9049944.

35. Lameire N, Rosenkranz B, Malerczyk V, Lehr KH, Veys N, Ringoir S. Ofloxacin pharmacokinetics in chronic renal failure and dialysis. Clin Pharmacokinet. 1991;21(5):357-71. doi: 10.2165/00003088-199121050-00004. PubMed PMID: 1773550.

36. Ogilvie RI. Clinical pharmacokinetics of theophylline. Clin Pharmacokinet. 1978;3(4):267-93. PubMed PMID: 354635.

37. Celardo A, Traina GL, Jankowski A, Bonati M. Pharmacokinetics of theophylline and its metabolites in rabbits. European journal of drug metabolism and pharmacokinetics. 1985;10(4):279-88. PubMed PMID: 3830715.

38. El-Yazigi A, Sawchuk RJ. Theophylline absorption and disposition in rabbits: oral, intravenous, and concentration-dependent kinetic studies. J Pharm Sci. 1981;70(4):452-6. PubMed PMID: 7229966.

39. Karbownik A, Szalek E, Sobanska K, Polom W, Grabowski T, Biczysko-Murawa A, et al. The effect of sunitinib on the plasma exposure of intravenous paracetamol and its major metabolite: paracetamol glucuronide. European journal of drug metabolism and pharmacokinetics. 2015;40(2):163-70. doi: 10.1007/s13318-014-0191-z. PubMed PMID: 24676873; PubMed Central PMCID: PMC4426134.

40. Bienert A, Kaminska A, Olszewski J, Gracz J, Grabowski T, Wolc A, et al. Pharmacokinetics and ocular disposition of paracetamol and paracetamol glucuronide in rabbits with diabetes mellitus induced by alloxan. Pharmacological reports : PR. 2012;64(2):421-7. PubMed PMID: 22661194.

41. Laskin OL. Clinical pharmacokinetics of acyclovir. Clin Pharmacokinet. 1983;8(3):187-201. PubMed PMID: 6342900.

42. van Jaarsveld MF, Walubo A, du Plessis JB. Interaction between valproic acid and acyclovir after intravenous and oral administration in a rabbit model. Basic & clinical pharmacology & toxicology. 2007;101(6):434-40. doi: 10.1111/j.1742-7843.2007.00134.x. PubMed PMID: 18028106.

43. Good SS, de Miranda P. Metabolic disposition of acyclovir in the guinea pig, rabbit, and monkey. The American journal of medicine. 1982;73(1A):91-5. PubMed PMID: 7102713.

44. Tsuji A, Nishide K, Minami H, Nakashima E, Terasaki T, Yamana T. Physiologically based pharmacokinetic model for cefazolin in rabbits and its preliminary extrapolation to man. Drug metabolism and disposition: the biological fate of chemicals. 1985;13(6):729-39. PubMed PMID: 2867880.

45. Ishikawa T, Koizumi N, Mukai B, Utoguchi N, Fujii M, Matsumoto M, et al. Pharmacokinetics of acetaminophen from rapidly disintegrating compressed tablet prepared using microcrystalline cellulose (PH-M-06) and spherical sugar granules. Chemical & pharmaceutical bulletin. 2001;49(2):230-2. PubMed PMID: 11217114.
